# The molecular architecture of the Dam1 kinetochore complex is defined by cross-linking based structural modelling

**DOI:** 10.1038/ncomms9673

**Published:** 2015-11-12

**Authors:** Alex Zelter, Massimiliano Bonomi, Jae ook Kim, Neil T. Umbreit, Michael R. Hoopmann, Richard Johnson, Michael Riffle, Daniel Jaschob, Michael J. MacCoss, Robert L. Moritz, Trisha N. Davis

**Affiliations:** 1 Department of Biochemistry, University of Washington, Seattle, Washington 98195, USA; 2 Department of Chemistry, University of Cambridge, Cambridge CB2 1EW, UK; 3 Institute for Systems Biology, Seattle, Washington 98109, USA; 4 Department of Genome Sciences, University of Washington, Seattle, Washington 98195, USA

## Abstract

Accurate segregation of chromosomes during cell division is essential. The Dam1 complex binds kinetochores to microtubules and its oligomerization is required to form strong attachments. It is a key target of Aurora B kinase, which destabilizes erroneous attachments allowing subsequent correction. Understanding the roles and regulation of the Dam1 complex requires structural information. Here we apply cross-linking/mass spectrometry and structural modelling to determine the molecular architecture of the Dam1 complex. We find microtubule attachment is accompanied by substantial conformational changes, with direct binding mediated by the carboxy termini of Dam1p and Duo1p. Aurora B phosphorylation of Dam1p C terminus weakens direct interaction with the microtubule. Furthermore, the Dam1p amino terminus forms an interaction interface between Dam1 complexes, which is also disrupted by phosphorylation. Our results demonstrate that Aurora B inhibits both direct interaction with the microtubule and oligomerization of the Dam1 complex to drive error correction during mitosis.

## Abstract (web summary)

The Dam1 complex binds kinetochores to microtubules during mitosis. Here the authors combine cross-linking/mass spectrometry with structural modelling to derive a structure for the Dam1 complex that changes when bound to microtubules; further, they provide a mechanism for regulation by Aurora B kinase.

## Graphical abstract

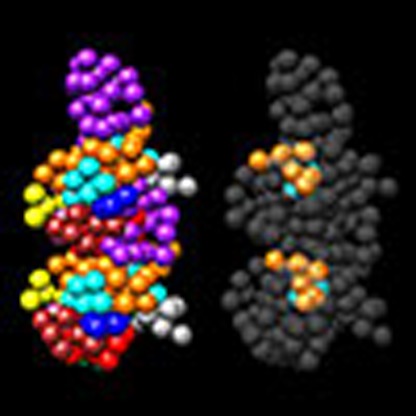

The kinetochore is a network of protein complexes that assemble on centromeric DNA and mediate the attachment of chromosomes to dynamic spindle microtubules (MTs). This attachment allows chromosomes to be equally segregated into daughter cells^1^. The yeast Dam1 complex is composed of ten proteins and is essential to attach kinetochores to MTs*. In vitro*, the Dam1 complex recapitulates many functions of the kinetochore, including the ability to couple cargo to dynamic MTs, while bearing load^2^,^3^,^4^,^5^,^6^,^7^,^8^,^9^. In solution, the Dam1 complex exists primarily as a 406-kDa dimer^10^. In the presence of MTs, however, it oligomerizes into rings encircling the MT^3^,^11^,^12^,^13^,^14^. Such oligomerization is necessary for the Dam1 complex to form MT attachments that are robust against tension^9^.

The ten Dam1 complex proteins are as follows: Ask1p (32 kDa), Dad1p (11 kDa), Dad2p (15 kDa), Dad3p (11 kDa), Dad4p (8.2 kDa), Dam1p (38 kDa), Duo1p (27 kDa), Hsk3p (8.1 kDa), Spc19p (19 kDa) and Spc34p (34 kDa). The functions of few of the subunits are known. Biochemical and genetic evidence showed that Dam1p and Duo1p are primarily responsible for MT binding^2^,^3^,^10^,^13^,^14^,^15^. In the absence of Hsk3p, the remaining nine components of the Dam1 complex split into two subcomplexes as follows: (A) Dam1p, Duo1p, Spc34p, Spc19p, Dad1p and Dad3p, which binds MTs but does not oligomerize, and (B) Ask1p, Dad2p and Dad4p, which does not bind MTs^9^,^15^.

Errors in chromosome segregation are devastating to the cell and, in multicellular organisms, are associated with a range of diseases as well as being a hallmark of cancer^16^. Intricate error-correction mechanisms exist to prevent such errors and delay cell cycle progression until correct kinetochore–MT attachments are achieved^17^. A major component of these protective pathways is the Ipl1p (Aurora B) kinase, which phosphorylates kinetochore proteins to promote detachment of incorrectly attached kinetochores from MTs^18^,^19^. As the Dam1 complex plays a central role in mediating kinetochore–MT coupling, it is an essential target of Ipl1p^20^.

The Dam1 complex is phosphorylated by Ipl1p at six different sites: Dam1p S20, S257, S265, S292, Ask1p S200 and Spc34p T199 (ref. 20). The four Dam1p sites have been shown to play a role in regulating the ability of Dam1 complex to bind MTs and mutations that block phosphorylation at all four sites are lethal. However, either the N-terminal site alone or the three C-terminal sites are sufficient for viability, suggesting functional redundancy^20^. Moreover, removal of the C terminus of Dam1p reduces the affinity of the Dam1 complex for MTs but does not abolish binding completely^13^,^14^. We previously found that phosphorylation of Dam1p S20 decreases the apparent affinity of the Dam1 complex to MTs, whereas phosphorylation of the C-terminal Ipl1 sites together has little effect^2^. Thus, both N- and C-terminal regions of Dam1p have been implicated in MT binding, although their specific roles remain elusive. Despite much work, key questions remain regarding how Ipl1 phosphorylation of Dam1p regulates the kinetochore–MT interface during mitosis.

One challenge that has prevented further progress is a lack of detailed structural information. Previous work culminated in a model for the layout of the Dam1 complex within a 30-Å electron microscopy (EM)-based structure, wherein five of the ten protein subunits were localized^3^. Subunit localization was performed in the absence of MTs, but previous studies have conflicted over whether the Dam1 complex binds MTs and can form oligomeric rings without a major conformational change^3^,^10^. In addition, the specific regions involved in the MT-binding interface have not been identified and the localization of half of the subunits remains unknown.

Here we used integrative structural modelling^21^, combining information from cross-linking/mass spectrometry (MS), previously published EM^3^ and biochemical data^15^, to determine the molecular architecture of the Dam1 complex. The work presented here is the first time a data set consisting primarily of cross-linking distance restraints has been used to produce a coarse-grained, yet biologically informative structural model of a large protein complex for which no X-ray structures nor homology models were available for any of its components. We then tested key predictions generated from this structural model, to define how Aurora B regulates the Dam1 complex during error correction.

## Results

### A simple accurate and high-yield cross-linking method

We benchmarked our cross-linking method using three protein complexes of known structure: the four-protein 300-kDa γ-TuSC complex, the two-protein 27-kDa BRCA1/BARD1 complex and the three-protein 120-kDa SCF^FBXL3^ ubiquitin ligase complex. Using our simple unlabelled method and a false discovery rate (FDR) of 1%, we obtained 129, 57 and 67 unique distance restraints (UDRs) from these 3 complexes, respectively. Agreement with available structures was excellent, with 91%, 81% and 94%, respectively, of cross-linked lysine Cα–Cα distances within the 30 Å that our cross-linker can span^22^ (Supplementary Fig. 1 and Supplementary Tables 1–4). A comparison of lysine Cα–Cα distances observed cross-linked together versus all possible lysine Cα–Cα distances from the structures showed a clear enrichment of distances <20 Å at 1% FDR, indicating our method accurately reports on the native structure (Supplementary Fig. 2). We chose this same conservative 1% FDR cutoff for modelling data. Cross-linker reactivity and MS detection were also good, as we detected 69–88% of lysines modified by the cross-linker (Supplementary Table 1). These data demonstrate our cross-linking method produces a high number of accurate distance restraints on complexes of various size and complexity. The number of unique cross-link identifications made by our method was greater than other methods (Supplementary Table 5). Our method also has key advantages: it performs well without isotopic labelling, post cross-linking fractionation or enrichment of cross-linked peptides. Data analysis using our Kojak cross-link identification algorithm is simple, fully automated and faster than existing methods^23^.

### The molecular architecture of free Dam1 complex

Our cross-linking analysis of free Dam1 complex in the absence of MTs identified 678 UDRs at 1% FDR (Supplementary Tables 1 and 6). Despite our simple method, this number (about 34 UDRs per 10 kDa of protein) compares very favourably with cross-linking analyses of other protein complexes (Supplementary Table 5)^24^,^25^,^26^,^27^. The cross-linking data were specific, because several pairs of proteins show multiple cross-links over all or part of their lengths (Supplementary Fig. 3a), whereas others exhibited little or no cross-linking to each other, even though lysines modified as ‘mono-links' were detected throughout these proteins (Supplementary Fig. 3b and Supplementary Tables 6–8). With the high information content of our cross-linking data set, we sought to expand on existing information to build a complete model of the molecular architecture of the Dam1 complex. Our modelling procedure consists of generating structural models consistent with the input experimental data plus physical and statistical considerations. This procedure has four stages: (1) gathering information, (2) selecting a suitable representation of the system and encoding experimental data into spatial restraints, (3) the generation of structural models and (4) analysis^28^. We used a coarse-grained description of the system and we tuned the resolution based on our cross-linking data. On average, we have 1 cross-linked residue every 13 amino acid residues along a protein chain. We therefore represented each Dam1 complex component as a chain of beads, each bead representing the average volume occupied by ten residues. A granularity much smaller than ten residues per bead would have resulted in modelling a large number of beads based on no information, but chain connectivity, leading to less precise models for large fragments of each component. On the other hand, a granularity much larger than ten residues per bead would not have optimally exploited the information available from the cross-linking data.

**Figure 1:**
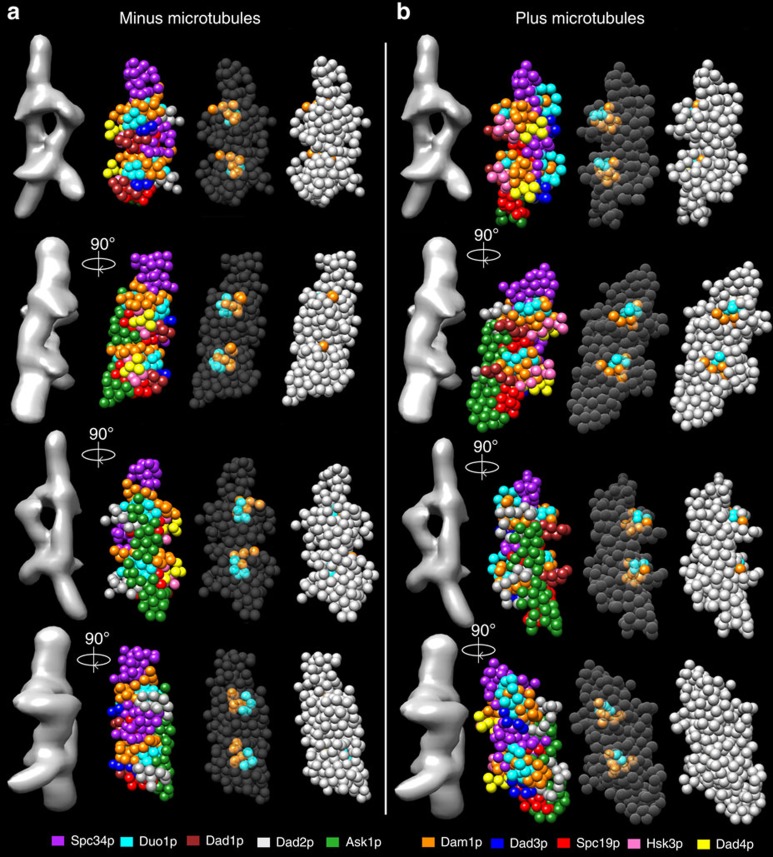
Structural model of the Dam1 complex. Structural model of the Dam1 complex in the (**a**) absence and (**b**) presence of MTs. Each model represents the centre of the single cluster found in the top 1,000 models. Four orientations are shown. Each panel shows the Dam1 EM structure EMD-1372 for reference (left) followed by 3 differently coloured versions of the Dam1 structural model. The left version shows all the beads coloured according to protein (key given at the bottom of the figure). The centre version highlights only those residues that cross-link to MTs. Transparency is used to make the MT-binding region visible. The right-hand version removes the transparency so that the proximity of the MT-binding region to the model's surface can be clearly seen. It is noteworthy that in the absence of MTs, the MT-binding region is in the interior of the structure.

**Figure 2:**
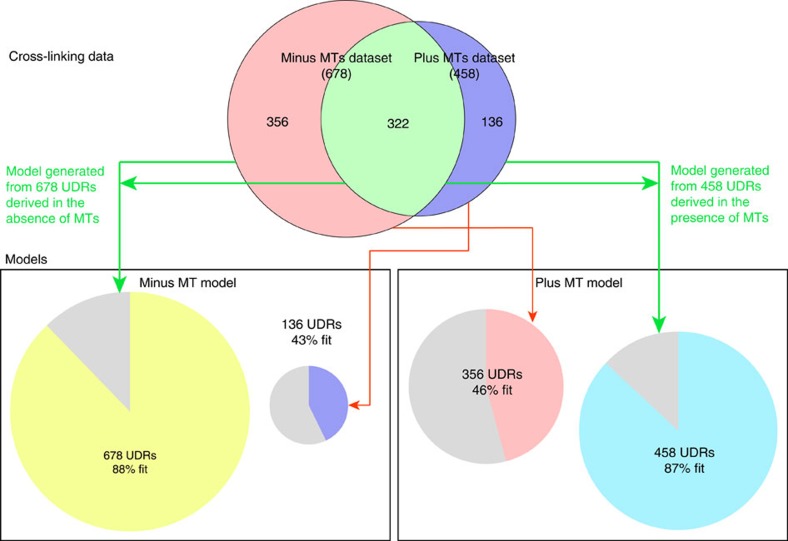
The number and overlap of distance restraints generated by cross-linking experiments on the Dam1 complex in the presence and absence of MTs. Per cent fit of distance restraint data with the minus MT and plus MT models generated is shown in boxes. Data are shown for peptides with Percolator^49^ assigned *q*-values ≤0.01. A distance restraint is considered a ‘fit' if the modelled bead surfaces of the two beads that included the cross-linked residues are within 10 Å. The minus MT model was generated using the 678 UDRs observed in the absence of MTs. The plus MT model was generated using the 458 UDRs observed in the presence of MTs. Per cent fit is shown for the data used to generate the models (green arrows) and for the data unique to the alternate condition (red arrows).

**Figure 3:**
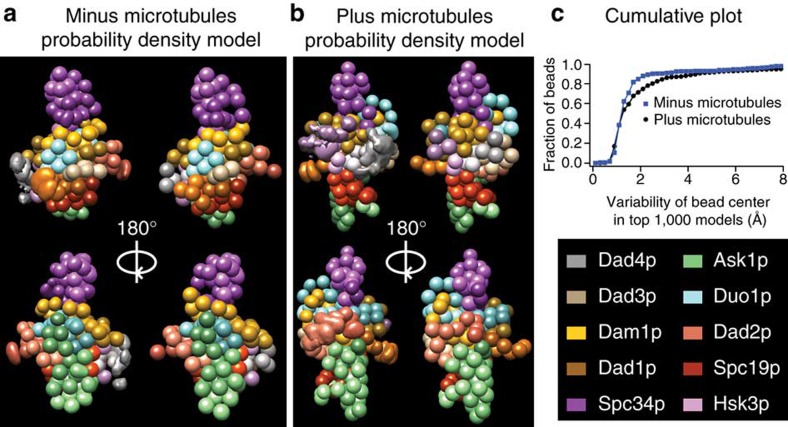
The precision of our modelling approach in determining the positions of all beads. (**a**,**b**) Density maps expressing the probability that a given region of space is occupied by a bead in the top 1,000 scoring models. The space was discretized in bins of 2 Å size. The density maps of each bead are shown at half their maximum value. It is noteworthy that almost all beads are well determined and therefore look similar in the probability density map and the representative model. An exception would be the Dad2p beads (salmon color) in the presence of MTs (bottom right image). Only a Dam1 complex monomer is shown for clarity. (**c**) A cumulative plot of bead variability.

Previous work localized single termini from five of the ten Dam1 complex protein components within the EM reconstruction of a Dam1 complex dimer^3^. We used the Integrative Modeling Platform (IMP)^21^, to model our cross-linking data combined with biochemical data^15^ plus the EM localization data (Supplementary Fig. 4; see Methods for details). We produced a complete structural model of the Dam1 complex, which localizes, for the first time, all ten components of the Dam1 complex at a resolution of ten amino acid residues per bead (Fig. 1, Supplementary Movie 1 and Supplementary File 1). This model fit 88% of the observed cross-linking data (Fig. 2, left green arrows). A probability density function representing the variability in the location of the beads in the top-scoring 1,000 models shows that the positions of 90% of the beads are tightly constrained, in that their centres vary by 3 Å or less at half maximal height (Fig. 3a). This model for the Dam1 complex in solution was used as a basis for comparison, to understand how the Dam1 complex interacts with MTs and if this is accompanied by substantial conformational changes.

### Dam1 complex–MT interactions specify a preferred orientation

**Figure 4:**
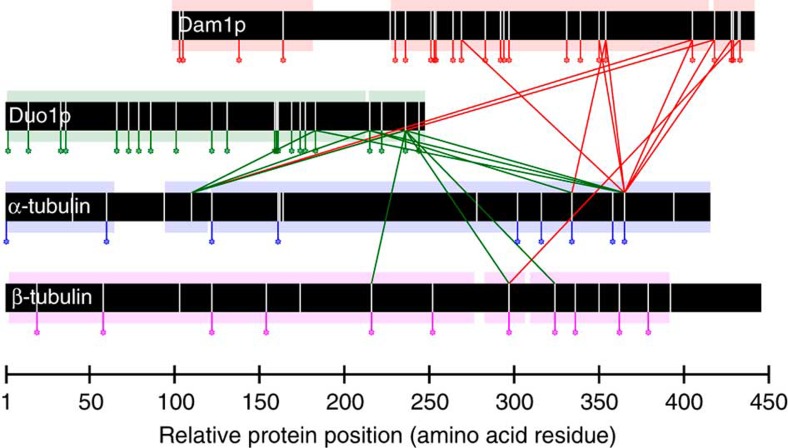
Dam1 complex to MT cross-links. Peptide sequence coverage (coloured boxes), mono-links (coloured vertical lines and circles) and lysines (vertical white lines) are also shown. Dam1p to Duo1p and tubulin to tubulin cross-links are hidden for clarity. Data are shown for peptides with Percolator^49^ assigned *q*-values ≤0.01.

We next performed cross-linking analysis on the Dam1 complex bound to MTs. Examination of the cross-linked Dam1 complex by EM showed its assembly into rings around MTs, as expected^3^,^11^,^12^,^13^,^14^ (Supplementary Fig. 5). Of the ten proteins that make up the Dam1 complex, only the C-terminal domains of Dam1p and Duo1p cross-linked to the MT (Fig. 4 and Supplementary Table 9). No other proteins formed a single cross-link to the MT, indicating our results were highly specific. We also detected cross-links between tubulin subunits, all of which agree with published structures and provide further confidence that our cross-links accurately report on the native structure (Supplementary Fig. 6 and Supplementary Table 10).

At 1% FDR, this analysis identified 458 UDRs within the Dam1 complex, with 90% of lysines modified by the cross-linker at least once (Supplementary Tables 1 and 6). As described above, we used IMP to generate a structural model for the Dam1 complex in the presence of MTs (Fig. 1b, Supplementary Movie 2 and Supplementary File 2). Eighty-seven per cent of the observed distance restraints were satisfied by this model (Fig. 2, right green arrows). The probability density function for the top-scoring 1,000 models reveals that 86% of the beads are tightly constrained (their positions vary by 3 Å or less at half maximal height), indicating this model is also well determined (Fig. 3b).

**Figure 5:**
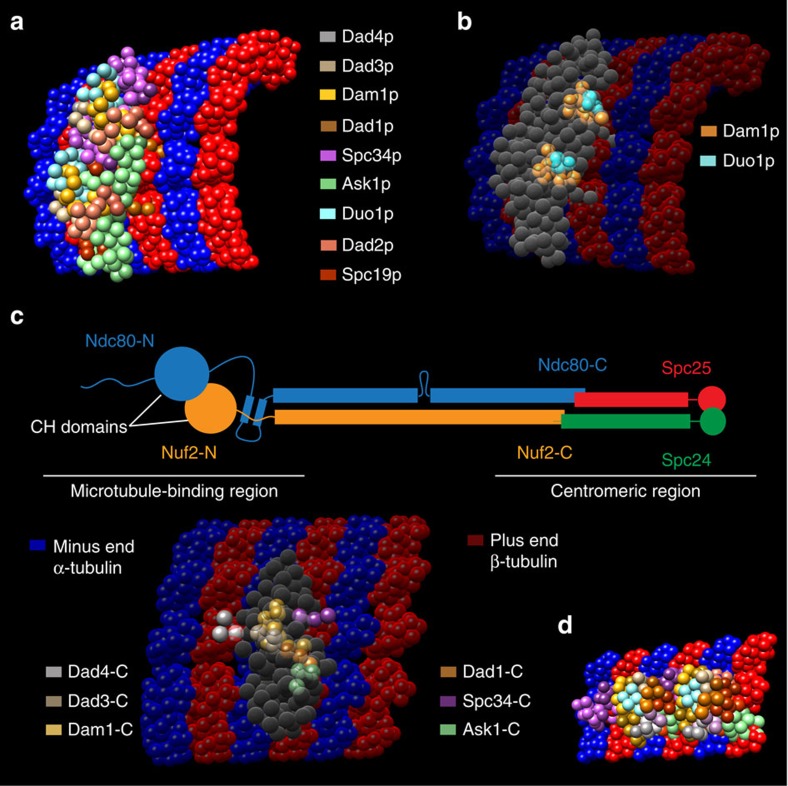
Cross-link based flexible docking of the Dam1 complex model onto the MT specifies a preferred orientation. (**a**) The model of the Dam1 complex on the MT with beads coloured by protein (Hsk1p is not visible at this angle). (**b**) Only those beads found cross-linked to the MT are highlighted. (**c**) The orientation of the Dam1 complex on the MT with respect to the orientation of the Ndc80 complex. The C-terminal MT-binding region of the Ndc80 complex is nearer the minus end of the MT. Aravamudhan *et al*.^29^ measured FRET between N-Nuf2 and six Dam1 complex C termini^29^. The proteins in the key to the left and right of the model represent these results, which indicates proximity between the C termini of Dam1p, Dad4p and Dad3p to the N terminus of Nuf2, while suggesting that the C termini of Spc34p, Ask1p and Dad1p are further away. Only a Dam1 complex monomer is shown for clarity. (**d**) Attempts to dock the model of the Dam1 complex in the minus MT conformation resulted in a poor fit and an orientation incompatible with a ring.

We then used a flexible-docking procedure to fit this model of the Dam1 complex dimer onto a MT, based on the cross-links observed between the Dam1 complex and tubulin (Figs 4,5, Supplementary Movie 3 and Supplementary File 3). A good fit was obtained, as 76% of the observed Dam1 complex to tubulin cross-links were satisfied (Supplementary Table 11). Furthermore, the top 3,264 models produced a single preferred orientation with respect to MT polarity (as depicted in Fig. 5), while alternative orientations were only found in low-scoring models. The orientation observed is compatible with ring formation, as the Dam1 dimer is arranged perpendicular to the protofilaments. Moreover, the orientation is consistent with recently published *in vivo* fluorescence resonance energy transfer (FRET) data^29^ (Fig. 5c).

### The Dam1 complex adopts a distinct MT-bound conformation

Of the 458 UDRs observed in the presence of MTs, 322 were shared with the data set obtained in the absence of MTs, suggesting that many interactions remained unchanged (Supplementary Table 7 and Supplementary Fig. 3a). However, 136 distance restraints were specific to Dam1 complex in the presence of MTs and, conversely, this data set did not contain 356 that were observed in the absence of MTs (Fig. 2). Of the 136 MT-specific cross-links, only 43% are satisfied by the model for Dam1 complex in the absence of MTs (Fig. 2, left red arrow). The reverse is also true: only 46% of the 356 cross-links found exclusively in the absence of MTs are satisfied by the model for the Dam1 complex in the presence of MTs (right red arrow). Attempts to dock the model of the Dam1 complex made in the absence of MTs onto a MT resulted in a poor fit and an orientation incompatible with a ring: just 29% of the observed Dam1 complex to tubulin cross-links were fit (Fig. 5d and Supplementary Table 11). These findings indicate that several interaction interfaces are strongly affected on MT binding and suggest that the Dam1 complex undergoes significant conformational changes (Supplementary Table 8 and Supplementary Fig. 3c).

**Figure 6:**
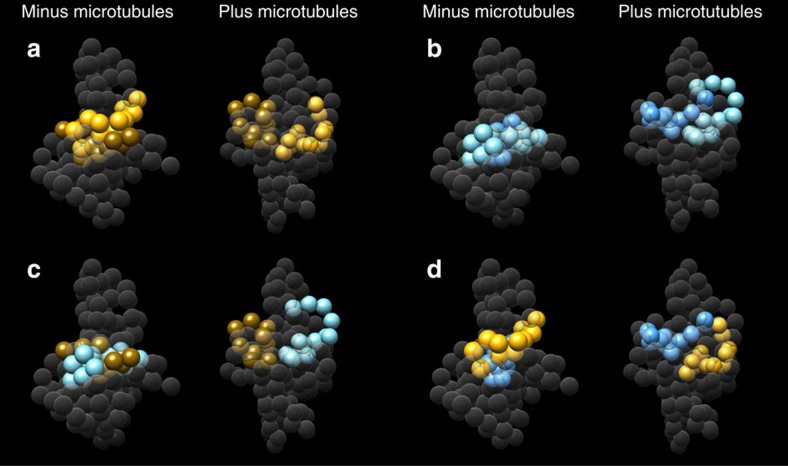
The Dam1 complex MT-binding domain. Structural models show that the C-terminal regions of Dam1p and Duo1p interact with the N-terminal regions of Dam1p and Duo1p in the absence of MTs. On binding MTs, these interactions are lost, freeing the C-terminal regions of Dam1 and Duo1, and allowing them to bind the MT. (**a**) Dam1N (light yellow) to Dam1C (dark yellow); (**b**) Duo1N (turquoise) to Duo1C (blue); (**c**) Dam1C (dark yellow) to Duo1N (turquoise); (**d**) Dam1N (light yellow) to Duo1C (blue). The Dam1 complex is shown as a monomer.

To examine these conformational changes in more detail, we analysed the interactions at a coarse structural level. We divided each protein into ‘macro-regions' of ∼100 amino acid residues (Supplementary Table 12). For example, Ask1p was divided into three macro-regions: Ask1N is the N-terminal 97 amino acids, Ask1M is the middle 97 amino acids and Ask1C is the C-terminal 98 amino acids. Proteins under 100 amino acids, such as Dad1p, Dad2p and so on consisted of just one macro-region: the whole protein. We then compared the interactions of each macro-region in the presence and absence of MTs (Supplementary Table 13). A striking difference is seen for Dam1C and Duo1C. In the absence of MTs, Dam1C and Duo1C interact with Dam1N and Duo1N. When bound to MTs, these interactions are not present (Fig. 6 and Supplementary Table 13, grey highlighting). Instead, Dam1C and Duo1C move to the surface of the complex and bind to the MT (Supplementary Movie 4).

**Figure 7:**
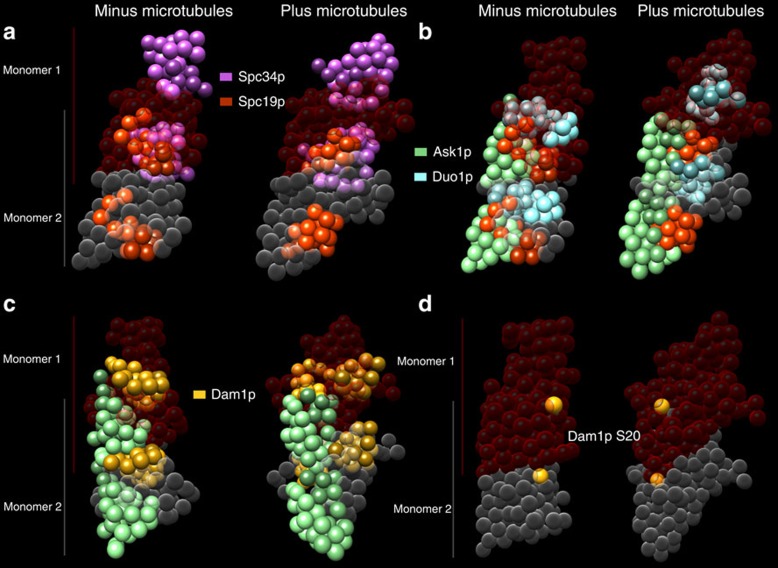
The Dam1 complex to Dam1 complex interface. In all panels, the Dam1 complex is shown as a dimer, with one monomer in red and the other in grey. (**a**) The interface between the two monomers is formed by multiple interactions between Spc19p and Spc34p in both the presence and absence of MTs. (**b**) In the presence of MTs, Duo1p more than doubles its interactions across the interface by binding to Spc19p and Ask1p. (**c**) On binding to MTs, Dam1N gains interactions with Ask1p and Dam1M, and Dam1C lose interactions with Ask1N. (**d**) The Aurora B kinase phosphorylation site Dam1p S20 lies at the interface between the two Dam1 complex monomers. Dam1p S20 beads are coloured yellow.

Binding to MTs also coincides with rearrangement of the interface between monomers of the Dam1 complex. As the Dam1 complex exists primarily as a dimer in solution, interactions between proteins within the complex can be within a monomer or between the monomers that make up the dimer. Both in the presence and absence of MTs, the interface between monomers is coordinated largely by Spc34p, which interacts with Spc19p (∼100 interactions; Supplementary Table 13, blue highlighting, Fig. 7a and Supplementary Movie 5), as well as with Dad2p and Dad3p (Supplementary Table 13, lilac highlighting). However, in the presence of MTs, Duo1p more than doubles its interactions across the interface by binding to Spc19p and Ask1p from the other monomer (Fig. 7b and Supplementary Movie 5). Dam1p interactions are also extensively remodelled in the presence of MTs. Dam1M and Dam1C both lose interactions with Ask1N in the other monomer, whereas Dam1N gains interactions with Ask1p (Fig. 7c, Supplementary Movie 5 and Supplementary Table 13, green highlighting). We hypothesize that these interactions, which cross the interface between monomers of the Dam1 complex and are remodelled in the presence of MTs, participate directly in the assembly and regulation of oligomeric rings.

### Aurora B kinase regulates Dam1 complex oligomerization

**Figure 8:**
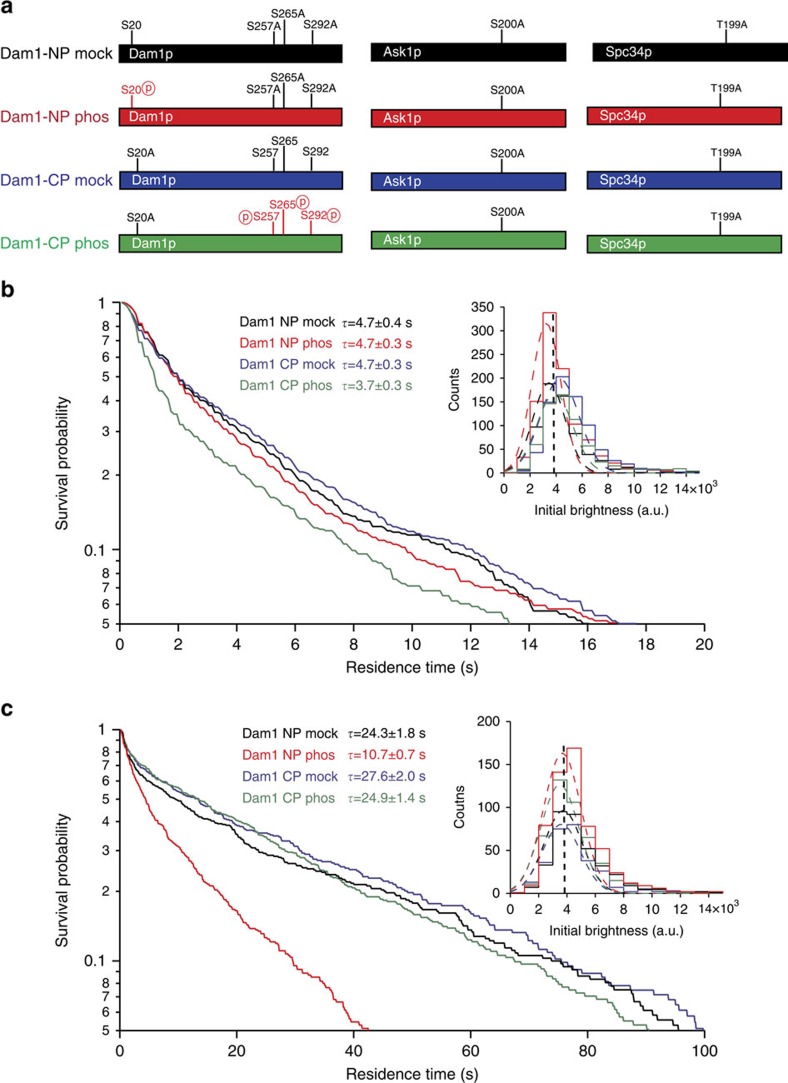
Aurora B kinase (Ipl1) phosporylation controls both MT binding and oligomerization of the Dam1 complex. (**a**) Schematic of phospho-blocking Dam1 complex constructs. Both constructs were phosphorylated by Ipl1 kinase in the presence of ATP. Mock-treated Dam1 complex had ATP substituted with distilled water. (**b**) Survival curve summary of single-molecule Dam1-GFP TIRF experiments. Dam1 NP mock (*n*=638), Dam1 NP phos (*n*=1009), Dam1 CP mock (*n*=777) and Dam1 CP phos (*n*=646) were each incubated at 40 pM for single-molecule Dam1–GFP complex imaging. Inset: GFP fluorescence (A.U.) distributions for Dam1 NP mock (3,500±1,200), Dam1 NP Phos (3,300±1,100), Dam1 CP mock (4,300±1,500) and Dam1 CP phos (3,900±1,400). (**c**) Survival curve summary of tracer Dam1-GFP TIRF experiments. GFP-tagged Dam1 NP mock (20 pM; *n*=360), Dam1 NP phos (*n*=587), Dam1 CP mock (*n*=294) and Dam1 CP phos (*n*=454) constructs were incubated with 2 nM corresponding non-tagged Dam1 constructs. Inset: GFP fluorescence (A.U.) distributions for Dam1 NP mock (3,700±1,300), Dam1 NP phos (3,700±1,300), Dam1 CP mock (3,600±1,400) and Dam1 CP phos (3,500±1,400) in tracer experiments. Inset panels show initial brightness of Dam1–GFP complex are similar across the whole TIRF data set, indicating that all data are from imaging single molecules of Dam1–GFP complexes either alone or incorporated into oligomers of unlabelled Dam1 complexes (in the tracer experiments). Vertical dashed line in inset panels represents the average height of single-step photobleach events under identical conditions (3,900±1,900 A.U., *n*=287). Average single-step photobleach duration under identical conditions is 130.9±5.7 s (*n*=287). All errors are s.d. of the mean.

The models presented here make testable predictions about the MT-binding and oligomerization interfaces of the Dam1 complex. In our models, the Dam1p S20 Aurora B phosphorylation site lies at the interface between the two Dam1 complex monomers (Fig. 7d and Supplementary Movie 5). Thus, we hypothesized that phosphorylation of Dam1p at S20 should specifically affect oligomerization of the complex rather than the interface between Dam1p and the MT. By contrast, phosphorylation of the three C-terminal sites of Dam1p should regulate the interface with the MT. We tested these predictions by measuring the binding of Dam1 complexes to MTs, both at the single-molecule level and when assembled into oligomers. First, we constructed two phospho-blocking mutants of the Dam1 complex (Fig. 8a): (1) ‘Dam1 CP', in which only the Dam1p C-terminal sites can be phosphorylated (Dam1p S20A, Ask1p S200A and Spc34p T199A) and (2) ‘Dam1 NP', in which only the Dam1p N-terminal S20 site can be phosphorylated (Dam1p S257A, S265A, S292A, Ask1p S200A, Spc34p and T199A). We used total internal reflection fluorescence (TIRF) microscopy to test the effects of phosphorylation on MT binding at the single-molecule level (40 pM Dam1–GFP)^9^. Consistent with our model, phosphorylation of the three C-terminal sites of Dam1p (Dam1 CP) decreased the residence time of single Dam1 complexes on MTs by 20%, whereas phosphorylation of the N-terminal S20 site (Dam1 NP) had no effect (Fig. 8b). To measure the effects of phosphorylation on oligomerization, we performed the TIRF assay at concentrations that support the formation of oligomeric rings (2 nM total Dam1 complex) wherein small ‘tracer' quantities of green fluorescent protein (GFP)-tagged Dam1 complex (1:100) were included to measure the behaviour of individual oligomeric particles, as previously described^9^. Relative to single molecules, the average residence time of non-phosphorylated Dam1–GFP complexes increased >5-fold, indicating their incorporation into oligomers of untagged Dam1 complexes (Fig. 8c). Tracer Dam1 CP complexes similarly exhibited increased residence time in the presence of excess untagged Dam1 CP complexes, indicating that phosphorylation of the C-terminal sites of Dam1p does not block oligomerization. However, Dam1 NP was defective in oligomerization, as indicated by a residence time increased by only twofold. These results confirm the predictions based on our model that phosphorylation of the Dam1p N terminus inhibits the ability of the Dam1 complex to form oligomers, whereas phosphorylation of the C-terminal sites regulates the interface with the MT.

## Discussion

Chemical cross-linking/MS has great potential for rapidly generating structural information and accurately defining large protein interaction networks^30^. Great progress in the cross-linking field has been made over the past few years^22^,^25^,^26^,^31^,^32^ and recent integrative work has begun to make use of such data^33^,^34^. The degree of success is dependent on the yield and accuracy of the data obtained. Given a large data set, such as presented here, the utility of the data can be dramatically increased through integrative structural modelling. This approach allowed us to combine cross-linking data with previously published EM and biochemical data, to determine the molecular architecture of the Dam1 complex. We further show that this approach produces biologically important structural models, allowing the formulation of clear and testable predictions.

Our data show that the C-terminal domains of Dam1p and Duo1p, exclusively, are cross-linked to the MT. Structural modelling shows these regions form a single compact domain in both the presence and absence of MTs. When bound to MTs, the complex adopts a distinct structural arrangement in which the C-terminal domains of Dam1p and Duo1p move to the surface of the complex and interact with the MT. A recent FRET study on the Dam1 complex in living cells placed the C-terminal region of Dam1p proximal to the MT^29^; however, two sets of EM studies conflict as to whether the C-terminal region of Dam1p lies proximal^10^ or distal^3^ to the MT. They differ in how the dimer Dam1 complex is docked into the EM reconstructions of the rings. As a C-terminal truncation of Dam1p (ΔC-Dam1) caused loss of a protrusion domain that was also missing in the reconstruction of the rings, Wang *et al*.^10^ concluded that the C- terminus of Dam1p was facing the MT. This required that dimer Dam1 complex undergo a conformational change to form a ring. Ramey and coworkers^3^ later proposed an alternative fit of the dimer Dam1 complex into the ring structure: one that did not require a conformational change for a good fit to be made but had the N-terminal domain of Dam1p facing the MT.

Our results strongly argue that the EM densities facing towards the MT are the Dam1p and Duo1p C-terminal regions in agreement with the first EM reconstruction^10^. We also observe that the conformation of the Dam1p complex changes on formation of rings around the MT. Finally, our data explain observations that deletion of (or phosphomimetic mutations in) the C-terminal domain of Dam1p decrease but do not abolish MT binding, as we show that the C-terminal region of Duo1p also interacts with the MT.

We used the cross-links observed between the Dam1 complex and tubulin, to fit our model of the Dam1 complex dimer onto a MT. During the docking procedure, Dam1 complex dimers were initialized in random orientations and thus sampled orientations incompatible with a ring (that is, parallel to the MT protofilaments). Despite this, the single preferred orientation determined was perpendicular to the MT and thus compatible with ring formation. Previous work found no evidence of a preferred orientation of Dam1 rings for facing either the plus or minus end of the MTs *in vitro*^14^. In contrast, our model predicts that the Dam1 complex does have a preferred orientation. Although our model is in agreement with previously published *in-vivo* FRET data^29^, the novel structural information presented in this paper lends weight to a different, but not contradictory, interpretation. As N-Nuf2 contains the MT-binding calponin homology domain, Aravamudhan *et al*.^29^ hypothesized that proteins showing FRET with N-Nuf2 are proximal to the MT lattice. By the same token, a lack of such FRET would indicate a protein's localization distal to the MT lattice. Although this may be true, a protein facing the minus end of the MT would have closer proximity with N-Nuf2 than a protein facing the plus end, as the Ndc80 complex is aligned along the MT with the calponin homology domains towards the minus end^35^ (Fig. 5c). The proteins that Aravamudhan *et al*.^29^ observed to have FRET with N-Nuf2 are also those that face the minus end in our model, that is, Dad4-C and Dad3-C. Moreover, the proteins that do not FRET with N-Nuf2 (Dad1-C, Spc34-C and Ask1-C) face the plus end of the MT in our model (Fig. 5c). Dam1-C takes a more central position in the complex proximal to the MT lattice. The agreement between our model and the FRET data is important, as it increases confidence in both independent studies.

Dam1p and Duo1p cross-link to multiple residues on the MT as does human Ska1, which is structurally unrelated but proposed to be functionally analogous to the Dam1 complex^31^. Similar to the Dam1 complex, the Ska1 complex forms oligomers^36^ and is regulated by Aurora B kinase^37^. Both the Dam1 complex and the Ska1 complex cross-link extensively to residues on the outside of the MT, although the interfaces do not obviously overlap. Whereas the majority of the cross-links between the Dam1 complex and the MT occur with α-tubulin (Supplementary Table 9), the majority of the Ska1 cross-links to the MT occur with β-tubulin^31^. Further work is necessary to resolve the significance of these differences.

The position of the Dam1 protein within the complex as presented in this work have important ramifications on the regulation of kinetochore attachment by Aurora B kinase. *In vivo*, mutation of the three C-terminal phosphosites in Dam1p confers mild defects such as sensitivity to benomyl, a MT depolymerizing drug, and synthetic lethality with mutations in *IPL1* (ref. 20). Here we find that phosphorylation of the C-terminal sites of Dam1p also has mild effects and decreases the affinity of the Dam1 complex for MTs, but only under conditions where the complex is a monomer. The same phosphorylation does not detectably affect the MT-binding affinity of the complex under oligomeric conditions, probably because the loss of affinity for the MT is largely overcome by the cooperativity of forming rings. Consistent with our results, the Dam1p C terminus is not required for Dam1 complex assembly nor for its accumulation at the MT tip^14^,^18^.

We have previously shown that phosphorylating the Dam1p C terminus, Ask1 S200 and Spc34 T199 together fully disrupt the interaction between the Dam1 and Ndc80 complexes^38^. We did not determine which phosphorylation site is responsible for disrupting this interaction, leaving the possibility that Dam1p C terminus may interact with the Ndc80 complex. The Dam1p C terminus was recently shown to interact with the Ndc80 complex through a yeast two-hybrid assay^18^. As phosphorylating the Dam1p C terminus mildly decreases the Dam1 complex's affinity to MTs, it is likely to be that this domain contains multiple functions that can be deciphered by further investigation.

Our previous work showed that oligomerization of the Dam1 complex is critically important to support stable bipolar attachment of sister chromatids to the mitotic spindle^9^. This is probably caused by a requirement for oligomerization of the Dam1 complex to couple cargo to disassembling MTs under tension. Oligomerization seems like an obvious target for stringent regulation of kinetochore attachments, but which site(s) was responsible for this regulation was unknown. Previous work showed that individual phospho-site mutations in the Dam1 complex have mild phenotypic consequences. However, several different phospho-site mutations confer severe phenotypes when combined with Dam1p S20A, suggesting that S20 is an important regulatory site. We previously showed that phosphorylation of Dam1p S20 reduces the apparent affinity for MTs but could not distinguish between an effect on affinity, cooperativity or both (see Supplementary Note 1). The new results presented here unite these observations. Our model of the molecular architecture of the Dam1 complex places S20 of Dam1p right at the interface between two monomers (far from the MT) and thus strongly predicts that phosphorylation of S20 should inhibit oligomerization. We show here that S20 phosphorylation has no effect on the direct interaction with the MT but a pronounced effect on oligomerization. Considering the importance of oligomerization for maintaining attachments to dynamic MTs, this presents a mechanism by which Aurora kinase B could potentially detach kinetochores by phosphorylation at a single site.

## Methods

### Reagents

Disuccinimidyl suberate (DSS) was from Pierce (Rockford, IL). Trypsin was from Promega (Madison, WI). Other reagents were from Sigma-Aldrich.

### Reference protein sequences and structures

Distance restraint location numbering is based on amino acid sequences listed in Supplementary Table 14. Reference structures for the proteins analysed were as follows: 1JM7.pdb (model 1) for BRCA1/BARD1 and 4I6J.pdb for SCF^FBXL3^. The MT structure was previously published^39^,^40^ as was the pseudo-atomic structure of the γ-TuSC^39^. For the Dam1 complex, EMDataBank file 1,372 was used. Structures generated in the study are presented in RMF format^21^ and are viewable in version 1.10.1 or higher of the UCSF Chimera^41^. Figures and movies based on these structures were made using Chimera.

### Protein expression and purification

γ**-**TuSC was expressed in Sf9 cells and purified using published methods^42^,^43^. Wild-type Spc97p and Spc98p plus Tub4p with an N-terminal 6 × His tag and tobacco etch virus cleavage site were used to produce γ-TuSC. Cell pellets from Sf9 cells grown in shaking flasks were lysed in HB100 lysis buffer: 40 mM HEPES, pH 7.5, 100 mM NaCl, 25 mM imidazole, 1% NP-40, 1 mM GTP, 0.1 mM EGTA, 1 mM phenylmethylsulfonyl fluoride. Complete mini-EDTA-free protease inhibitor cocktail tablets were added to the lysis buffer (Roche Applied Sciences, Indianapolis, IN). Purification was by nickel affinity using Bio-Scale Mini Profinity IMAC Cartridges (Bio-Rad, Hercules, CA) followed by anion exchange on a Mono Q 5/50 GL column (GE Healthcare Biosciences, Pittsburgh, PA). Peak fractions from the Mono Q eluted in 500 mM NaCl and were combined and stored at −80 °C in 40 mM HEPES, pH 7.5, 500 mM NaCl, 0.1 mM EGTA and 10% glycerol without further processing. Final γ-TuSC concentration was ∼1 mg ml^−1^. All ten Dam1 complex subunits were expressed in *Escherichia coli* (BL21 Rosetta; Novagen, Madison, WI) from a single polycistronic vector^44^. The complex was affinity purified using a C-terminal His6 × tag on Spc34p. The eluate was purified on a size-exclusion column (Superdex 200 10/300GL; Amersham Biosciences, Piscataway, NJ), equilibrated with 20 mM phosphate at pH 7.0, 500 mM NaCl. SCF^FBXL3^ ubiquitin ligase complex was a gift from Xing *et al*.^45^ BRCA1/BARD1 RING-domain heterodimer was a gift from Klevit and colleagues^46^.

### Cross-linking

For γ-TuSC, 146 μg protein was diluted with HB500 buffer (40 mM HEPES, 100 mM NaCl, pH 8) to a final volume of 331.2 μl. Cross-linker concentration was brought to 0.86 mM by adding 14.5 mM DSS. The reaction was allowed to proceed for 2 min at 25 °C before quenching with 26 μl 500 mM NH_4_HCO_3_. For BRCA1/BARD1 complex, 40 μg protein was diluted with PBS buffer to a final volume of 100 μl. Cross-linker concentration was brought to 0.86 mM by adding 14.5 mM DSS. The reaction was allowed to proceed for 2 min at 25 °C before quenching with 10 μl 500 mM NH_4_HCO_3_. For SCF^FBXL3^ ubiquitin ligase complex, protein was first buffer exchanged into HB200 buffer (40 mM HEPES, pH 7.5, 100 mM NaCl) using protein desalting spin columns (Pierce). Ten per cent glycerol was added to the desalted protein. Twenty micrograms protein was diluted with HB200 buffer to a final volume of 46.5 μl. Cross-linker concentration was brought to 0.06 mM by adding 2.9 mM DSS. The reaction was quenched with 7 μl 500 mM NH_4_HCO_3_ after 30 min at 25 °C. For Dam1 complex, 40 μg protein in 93 μl 500 mM NaCl 50 mM phosphate at pH 6.9 was brought to 0.48 mM DSS by adding 14.5 mM DSS. The reaction was quenched with 10 μl of 500 mM NH_4_HCO_3_ after 2 min cross-linking at 25 °C. For Dam1 complex on MTs, 20 μM tubulin was polymerized in BRB80 (80 mM PIPES, 1 mM MgCl2, 1 mM EGTA, adjusted pH to 6.9 using KOH) containing 1 mM GTP, 6 mM MgCl_2_, 3.8% dimethyl sulfoxide at 37 °C (total volume 100 μl). After 30 min polymerization, 100 μl BRB80 containing 10 μM taxol was added. MTs were then pelleted in a TLA100 rotor at 58,000 r.p.m. (130,000*g*) for 10 min at 37 °C. The pellet was resuspended with 200 μl warm BRB80 containing 10 μM taxol. For cross-linking, 10 μl MTs were mixed with 20 μg Dam1 complex in 100 μl BRB80 at 25 °C. The reaction was mixed by gentle flicking and was allowed to stand for 5 min. Three microlitres of 14.5 mM DSS in dimethyl sulfoxide was added and the mixture was allowed to cross-link for 2 min at 25 °C before quenching by addition of 10 μl of 0.5 M NH_4_HCO_3_. The quenched reaction was spun at 58,000 r.p.m. (130,000*g*) in a TLA100 rotor for 10 min at 37 °C and the resulting pellet was resuspended in 100 μl ice-cold HB500 buffer. After quenching, all reactions (except Dam1 on MTs) were buffer exchanged to HB500 using protein desalting spin columns (Pierce).

### Digestion

Cross-linked proteins were reduced with 10 mM dithiothreitol at 37 °C for 30 min, followed by 30 min alkylation at room temperature with 15 mM iodoacetamide. For Dam1 complex in the absence of MTs, 25% volume of heavy (H_2_^18^O) or light (unlabelled) water was added to the reactions before digestion with trypsin at a substrate-to-enzyme ratio of 60:1 overnight at room temperature, with shaking. Owing to a higher yield of cross-links obtained without isotopic labelling, only unlabelled reactions were performed for all other samples. Digested samples were acidified with 5 M HCl before being stored at −80 °C until analysis.

### Mass spectrometry

MS was performed on a Q-Exactive or Q-Exactive HF (Thermo Fisher Scientific). Sample digest (1.5 μg) was loaded by autosampler onto a 150-μm Kasil fritted trap packed with Jupiter C12 90 Å material (Phenomenex) to a bed length of 2 cm at a flow rate of 2 μl min^−1^. After loading and desalting using a total volume of 8 μl of 0.1% formic acid plus 2% acetonitrile, the trap was brought online with either a pulled fused-silica capillary tip (75-μm i.d.) or an empty Pico-Frit column (New Objective) that was self-packed with 30 cm of Reprosil-Pur C18-AQ (3-μm bead diameter, Dr Maisch) mounted in an in-house constructed microspray source and placed in line with a Waters Nanoacquity binary UPLC pump plus autosampler. Peptides were eluted off the column using a gradient of 2–35% acetonitrile in 0.1% formic acid over 120 min, followed by 35–60% acetonitrile over 10 min at a flow rate of 250 nl min^−1^.

The Q-Exactive mass spectrometer was operated using data-dependent acquisition where a maximum of six tandem MS (MS/MS) spectra were acquired per MS spectrum (scan range of *m/z* 400–1,600). The resolution for MS and MS/MS was 70,000 and 35,000, respectively, at *m/z* 200. The automatic gain control targets for both MS and MS/MS was set to 1e6 and the maximum fill times were 20 and 128 ms, respectively. The MS/MS spectra were acquired using an isolation width of 2 *m/z* and a normalized collision energy of 25. The underfill ratio was set to 0.3%, which corresponded to the requirement that the precursor ion threshold intensity be above 2.3e4, to trigger an MS/MS acquisition. MS/MS acquisitions were prevented for +1, +2 or undefined precursor charge states. Dynamic exclusion (including all isotope peaks) was set for 10 s. The Q-Exactive HF was operated similarly, except that repeat analyses employed slight variations in MS/MS resolution (30,000 and 60,000) or automatic gain control targets (2e4 and 1e5).

### Analysis of MS data

Mass spectra were converted into mzML using msconvert from ProteoWizard^47^. All proteins detectable were identified by searching high-resolution MS/MS spectra against whole-proteome databases using Comet^48^. Peptide identifications were processed with Percolator^49^ and used to create a smaller protein database for cross-link searches. These smaller databases consisted of all proteins identified at 1% FDR and contained 114, 64, 105, 302 and 159 proteins, respectively, for the search databases for γTuSC, BARD1/BRCA1, SCF^FBXL3^, free Dam1 complex and Dam1 complex on MTs. MSDaPl was used to visually inspect the Comet results^50^. Cross-linked peptides were identified using the Kojak version 1.0 cross-link identification software^23^ following the author's instructions (http://www.kojak-ms.org) and using the search parameters outlined in Supplementary Methods. Kojak results were exported to Percolator, to produce a statistically validated set of cross-linked peptide identifications at the desired FDR threshold (described in detail in Supplementary Methods). All distance restraint location numbering is based on the sequences in Supplementary Table 14.

A relational database, data processing software and a web application were developed to interrogate and visualize the data. All data presented in the current work are available at http://proxl.yeastrc.org/dam1-zelter-2015. In addition, this web application makes it possible to rapidly explore every peptide and peptide spectrum match associated with every distance restraint reported in this study. Furthermore, it is possible to view the annotated spectrum for each peptide spectrum match through an integrated spectrum viewer. Multiple other features are also available via this viewer to help the reader make a detailed exploration of our raw MS data and subsequent cross-link identifications.

### Dam1 complex phosphorylation

Expression of GST-Ipl1 was from plasmid pSB196 (Sue Biggins, Fred Hutchinson Cancer Research Center, Seattle, WA) at 23 °C for 2 h. GST-Ipl1 was purified using GSTrap HP, according to the manufacturer's instructions (GE Healthcare Biosciences), except that the elution buffer was 50 mM, 250 mM KCl, 10 mM glutathione, pH 8.0. pSB503 (Sue Biggins) was used to express GST-Sli15 (residues 554–698) at 37 °C. GST-Sli15 was purified using glutathione–Sepharose 4B resin, following the manufacturer's instructions, except that the elution buffer was 20 mM Tris buffer 200 mM NaCl, 1 mM β-mercaptoethanol, 1 mM EDTA, 10 mM glutathione, pH 8.0.

Ipl1p phosphorylation of recombinant Dam1 complex was carried out in a 50-μl reaction containing 4 μM Dam1 complex, 0.5 μM GST-Ipl1, 0.5 GST-Sli15 (residues 554-698), 200 mM NaCl, 10 mM ATP, 25 mM MgCl_2_ and 50 mM HEPES buffer, pH 7.4. Reactions were incubated for 90 min at 30 °C. Glycerol was added (5% final) before snap-freezing and storing at −80 °C. The mock/non-phosphorylated controls were carried out by substituting distilled water for the ATP. Under these conditions, we achieve nearly stoichiometric phosphorylation of the complex^2^.

### TIRF microscopy

Flow chambers were constructed using glass slides and functionalized coverslips as reported before^5^,^9^,^38^. Coverslip was adhered to a glass slide with double-sided tape, to form individual flow channels between two adjacent strips of tape. ‘Rigor' kinesin was added to each channel to nonspecifically bind to the coverslip. This allowed for the addition and immobilization of taxol-stabilized MTs. Single-molecule imaging experiments were carried out by incubating 40 pM phosphorylated or mock-treated Dam1-GFP NP (Dam1p S257A, S265A, S292A, Ask1p S200A and Spc34p T199A) or Dam1-GFP CP (Dam1p S20A, Ask1p A200A and Spc34p T199A) with Alexa-647-labelled MTs. GFP and Alexa-647 fluorescence channels were simultaneously recorded using a custom TIRF imaging system^5^.

In ‘tracer' assays, GFP-tagged and unlabelled versions of the mutant Dam1 complex were mixed to 1:100 ratio to a total concentration of 2 nM. Next, this solution was incubated with MTs. All TIRF assays were carried out in BRB80 in the presence of 0.8 mg ml^−1^ ϰ-casein, 50 mM KCl and an oxygen scavenger system (200 μg ml^−1^ glucose oxidase, 35 μg ml^−1^ catalase, 25 mM glucose and 5 mM dithiothreitol).

Single particle tracking and analysis was carried out with custom software (available on request) developed in Labview (National Instruments) and Igor Pro (Wavemetrics)^9^,^38^. Mean residence times were carried out through bootstrapping analysis^9^. Each residence time data set was randomly resampled with replacement 1,000 times. All of the data sets presented formed normal distributions. Gaussian fits to these distributions yielded estimates of mean residence time and s.d.

### Computational structural modelling

Analysis of the cross-linking data and structural modelling were carried out using IMP (http://integrativemodeling.org/)^21^,^51^. IMP is an open source platform for integrative structure determination based on EM data, FRET spectroscopy, cross-linking/MS, small angle X-ray scattering profiles and various proteomics data^33^,^34^,^52^,^53^,^54^,^55^,^56^,^57^,^58^.

### Modelling the Dam1 complex in the absence of MTs

As no crystallographic data is available for any components of the Dam1 complex, we used a coarse-grained representation in which one spherical bead was used to represent ten residues. Total volume of beads was estimated from known masses of proteins and standard assumptions about their densities^59^. Beads representing protein fragments adjacent in sequence were restrained by a harmonic function of the distance between their surfaces, with equilibrium value and spring constant equal to 0 Å and 1.0, respectively. An excluded volume restraint with intensity set at 1.0 was used to avoid steric clashes. As EM data are available only for Dam1 complex in dimeric form^3^, two identical copies of the complex were modelled. A symmetry constraint was used to lock the second copy in the symmetric position, which was determined from the EM map using UCSF Chimera's^41^ ‘Fit Map to Map' tool.

Cross-linking data were encoded as upper-bound harmonic restraints acting on the distance between surfaces of the two beads that included the cross-linked residues. We used our cross-linking analysis of the SCF^FBXL3^ complex to determine an appropriate value for this upper bound was 5 Å. This value fits a similar proportion of cross-linking data as an atomic Cα–Cα cutoff of 21 Å (Supplementary Table 15). We chose the more restrictive value, because the restraint can be marginally exceeded during modelling. As we modelled two copies of the complex, each cross-linking data point can be explained by a pair of residues belonging either to the same copy of the Dam1 complex (intra-complex) or to two different copies (inter-complex). We did not arbitrarily decide whether a cross-link should be satisfied by inter- or intra-complex pairs of beads. Instead, for each model generated, we calculated both distances and then applied the restraint to the smallest. A total of 678 UDRs were used in the modelling (Supplementary Table 6).

As the termini of half of the components were localized by previous studies^3^, the EM map was manually segmented using UCSF Chimera and the positions of Ask1p, Spc19p, Duo1p, Dam1p and Spc34p were confined into bounding boxes (Supplementary Fig. 4). The boxes used were purposefully larger than the EM localization data, to account for the location of whole proteins rather than the individual protein termini that were localized in the published study. We did not apply a hard constraint, but allowed each component to extend beyond its pre-assigned bounding box, to satisfy our cross-linking data. This was implemented by using harmonic restraints on the distance between the centre of each bead and the wall of the bounding box, acting only when a bead exited the box. In principle, each restrained Dam1 component can extend outside its bounding box by any given amount, provided that the violation of the restraint is balanced by the satisfaction of a sufficient number of cross-linking restraints. Deletion of the gene encoding Hsk3p causes the remaining nine components of the Dam1 complex to split into two subcomplexes: (A) Dam1p, Duo1p, Spc34p, Spc19p, Dad1p and Dad3p, and (B) Ask1p, Dad2p and Dad4p^15^. We therefore encoded a connectivity restraint among Ask1p, Dad2p and Dad4p, to ensure that each of the three proteins were in contact, that is, the distance between two beads was zero, with at least one of the other two. Sampling of the conformational space was carried out using a Monte Carlo algorithm coupled with the parallel tempering method^60^. To accelerate diffusion in temperature, parallel tempering was carried out in the well-tempered ensemble^61^. Thirty-four replicas were distributed in a temperature range between 1.0 and 3.0 *k*_B_*T*. Each bead was moved by a random translation of at most 1.0 Å. The initial positions of localized proteins were randomized inside the corresponding bounding box, those of the other components inside the union of all the bounding boxes. The top 1,000 scoring models obtained in 10^8^ Monte Carlo steps were clustered using the Daura *et al*.^62^ clustering algorithm, with a cutoff of 1.7 nm. To visualize the precision of our modelling approach in determining bead positions, we constructed density maps, which quantify the probability that a given region of space is occupied by a bead in the top 1,000 scoring models. The space was discretized in bins of 2 Å size. Density maps of each bead were then visualized at half their maximum value.

The models obtained were validated by data jackknifing tests. The Dam1 complex was modelled using three different cross-linking data sets, which were constructed by randomly choosing 95% of the data points. The resulting models conserved 91, 88 and 92% of the interactions of the model built with the entire data set.

### Modelling the Dam1 complex in the presence of MTs

To model the Dam1 complex in the presence of MTs, we used 458 UDRs (Supplementary Table 6) and applied the methods described above. At this stage, tubulin-to-Dam1 complex cross-links were not used in the modelling. The models obtained were validated by data jackknifing tests as before. The resulting models conserved 88, 87 and 91% of the interactions of the model built with the entire data set.

### Determining Dam1 complex–MT orientation

To identify the orientation of Dam1 complex on the MT, we used a flexible-docking procedure onto a coarse-grained (ten residues per bead) structural model of the MT based on atomic model of the MT^39^,^40^. A 6 × 6 lattice of α- and β-tubulin subunits was constructed. We added 21 Dam1 complex to MT cross-links (Supplementary Table 10) encoded as distance restraints acting on both Dam1 complex monomers and on the closest bead belonging to all α- and β-tubulin subunits. The 20 α- and β-tubulin subunits at the edge of the 6 × 6 lattice were excluded from the restraints calculation. All the other restraints used for the modelling of the Dam1 complex alone were maintained.

The flexible-docking procedure first positioned on the MT the Dam1 complex previously obtained, while optimizing positions of each bead. The coarse-grained tubulin structure was initialized in a random position and orientation 100 nm from the centre of the Dam1 complex. In a second stage, the relative orientation of the two Dam1 monomers was optimized by removing the symmetry constraints from the EM map. In this study, we discussed only the initial model of the Dam1 complex structurally aligned to the final model from the flexible-docking procedure, as it fit the data equally well. A similar approach was used to position the model of the Dam1 complex built with the cross-linking data collected in absence of MT on the MT.

## Additional information

**How to cite this article:** Zelter, A. *et al*. The molecular architecture of the Dam1 kinetochore complex is defined by cross-linking based structural modelling. *Nat. Commun.* 6:8673 doi: 10.1038/ncomms9673 (2015).

## Supplementary Material

Supplementary Information Supplementary Figures 1-6, Supplementary Tables 1-15, Supplementary Note 1, Supplementary Methods and Supplementary References

Supplementary Movie 1 Movie of structural model of the Dam1 complex derived from cross-linking data obtained in the absence of microtubules (v19). The 3 differently colored views correspond to the three views depicted in Figure 1a of the main text.

Supplementary Movie 2 Movie of structural model of the Dam1 complex derived from cross-linking data obtained in the presence of microtubules (v21). The 3 differently colored views correspond to the three views depicted in Figure 1b of the main text.

Supplementary Movie 3 Movie of structural model of the Dam1 complex (v21) docked onto the microtubule using observed tubulin to Dam1 complex crosslinks.

Supplementary Movie 4 Movie of structural models of the Dam1 complex transitioning between unbound and microtubule bound conformations. The movie shows the model derived from cross-linking data obtained in the absence of microtubules (v19) transitioning to the model of the Dam1 complex derived from cross-linking data obtained in the presence of microtubules (v21). The 3 differently colored views correspond to the three views depicted in Figure 1a of the main text. The models are docked onto the microtubule in the top scoring orientation for v21.

Supplementary Movie 5 The Dam1 complex to Dam1 complex interface (companion to Figure 7). Figure 7 should be used as a key to this movie. In all panels the Dam1 complex is shown as a dimer with one monomer in red and the other in gray. (a) The interface between the two monomersisformed by multiple interactions between Spc19p and Spc34p in both the presence and absence of microtubules. (b) In the presence of microtubules, Duo1p more than doubles its interactions across the interface by binding to Spc19p and Ask1p. (c) Upon binding to microtubules Dam1N gains interactions with Ask1p and Dam1M and Dam1C lose interactions with Ask1N. (d) The Aurora B kinase phosphorylation site Dam1p S20 lies at the interface between the two Dam1 complex monomers. Dam1p S20 beads are colored yellow.

Supplementary Data 1 Raw rmf file of structural model of the Dam1 complex derived from cross-linking data obtained in the absence of microtubules (v19). (Viewable in version 1.10.1 or higher of UCSF Chimera.)

Supplementary Data 2 Raw rmf file of structural model of the Dam1 complex derived from cross-linking data obtained in the presence of microtubules (v21). (Viewable in version 1.10.1 or higher of UCSF Chimera.)

Supplementary Data 3 Raw rmf file of structural model of the Dam1 complex derived from cross-linking data obtained in the presence of microtubules (v21) fit onto a microtubule. (Viewable in version 1.10.1 or higher of UCSF Chimera.)
