# Supplementary material for: The molecular architecture of the Dam1 kinetochore complex is defined by cross-linking based structural modelling
Source: Nat Commun. 2015 Nov 12;6:8673. doi: 10.1038/ncomms9673 (PMC4660060; doi:10.1038/ncomms9673)
Supplement: Supplementary Information — Supplementary Figures 1-6, Supplementary Tables 1-15, Supplementary Note 1, Supplementary Methods and Supplementary References [file ncomms9673-s1.pdf]

Supplementary Information:

Supplementary Figures

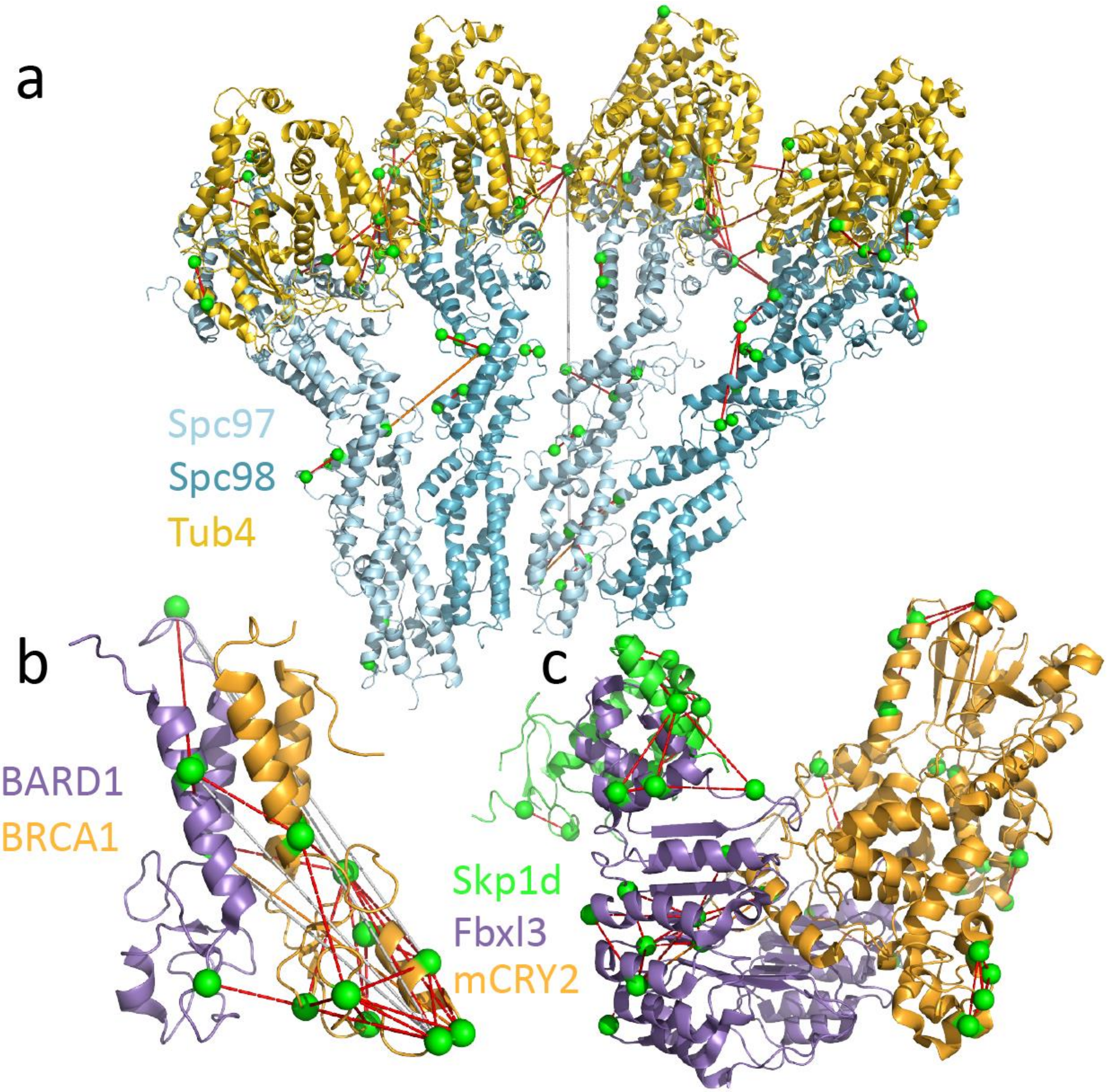

Supplementary Figure 1: Cross-linking data plotted on available structures. (a) Pseudo-atomic structure of the γ-TuSC; (b) Atomic structure of the BRCA1/BARD1 heterodimer; (c) Atomic structure of the SCF^{FBXL3} complex. Lines representing cross-links and loop-links are: red (Cα-Cα distance <30 Å); orange (Cα-Cα distance 30 to 40 Å); and grey (Cα-Cα distance >40 Å). Cross-linked lysine α-carbons are depicted as green spheres. Data is shown for peptides with Percolator[1] assigned *q* values <= 0.01.

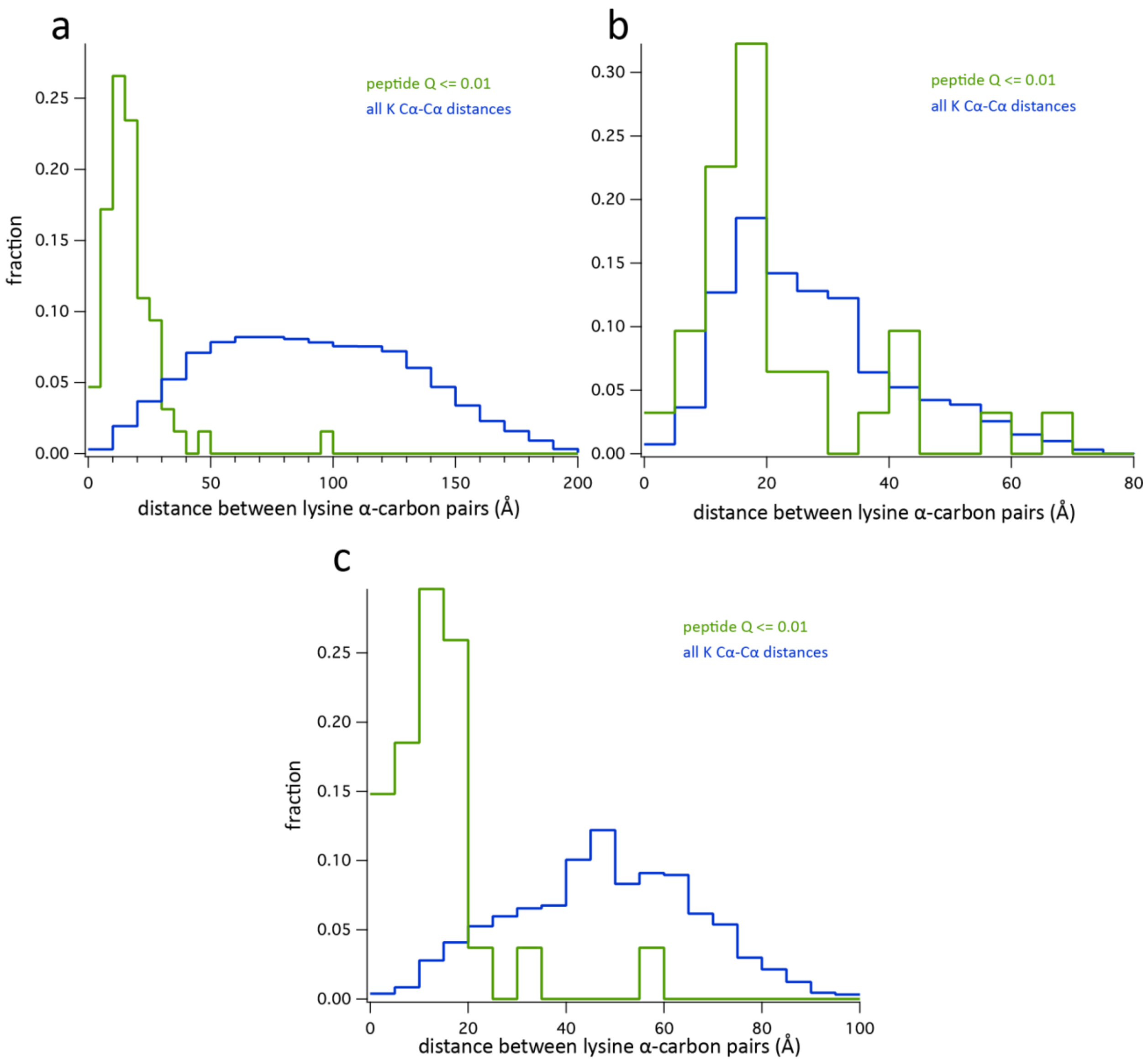

Supplementary Figure 2: Cross-linking method benchmarking. Histograms of lysine Cα-Cα distances observed cross-linked together versus all possible pairs of lysine Cα-Cα distances based on atomic structures. Data is shown for peptides with Percolator[1] assigned *q* values <= 0.01. (a) γ-TuSC; (b) BRCA1/BARD1; and (c) SCF^{FBXL3}. Hits to peptides not present in reference PDBs are not shown.

a

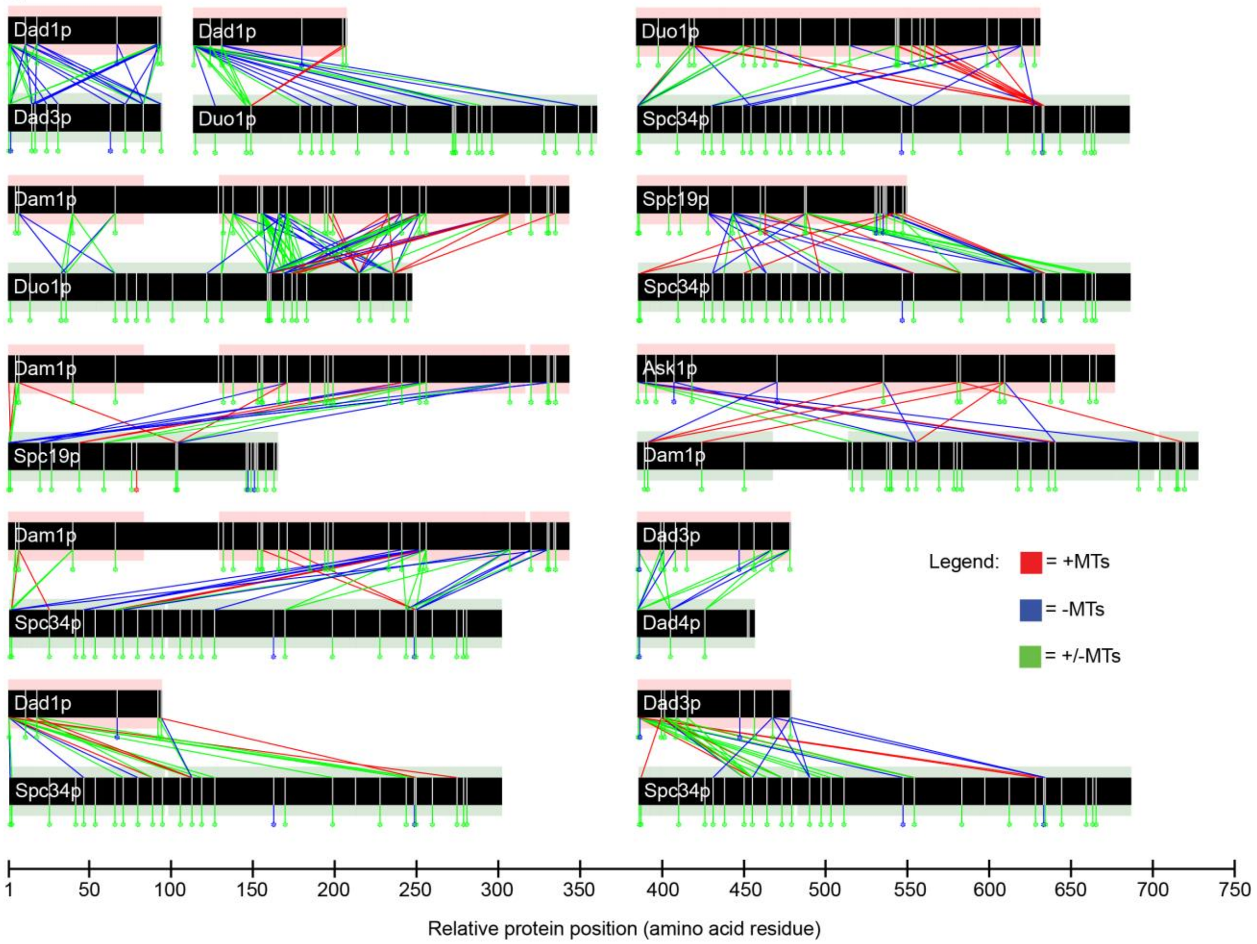

Dad1p
Dad3p
Dad1p
Duo1p
Duo1p
Spc34p
Dam1p
Duo1p
Spc19p
Spc34p
Dam1p
Spc19p
Ask1p
Dam1p
Dam1p
Spc34p
Dad3p
Dad4p
Dad1p
Spc34p
Dad3p
Spc34p
Legend:
= +MTs
= -MTs
= +/-MTs
1 50 100 150 200 250 300 350 400 450 500 550 600 650 700 750
Relative protein position (amino acid residue)

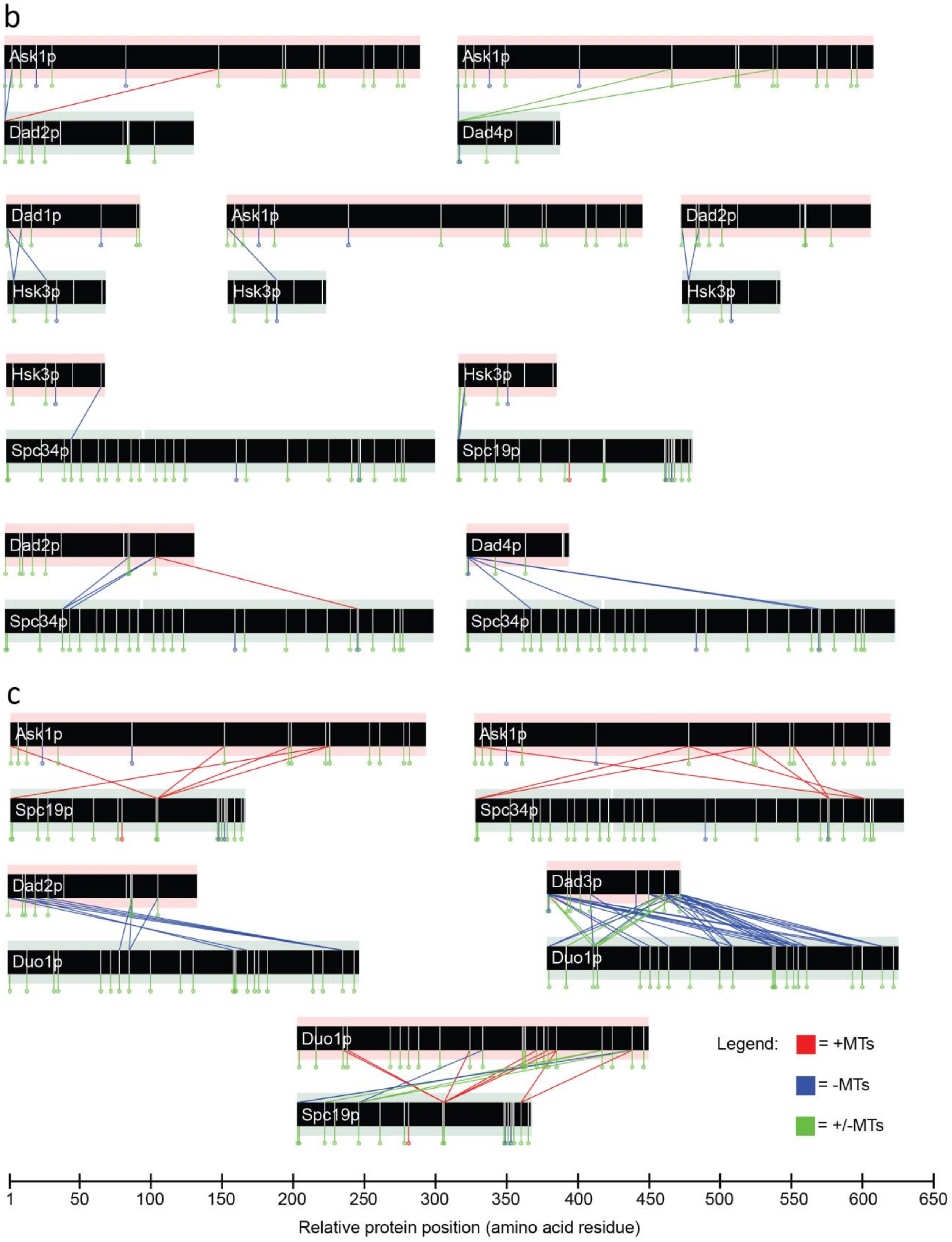
b
Ask1p
Dad2p
Ask1p
Dad4p
Dad1p
Hsk3p
Ask1p
Hsk3p
Dad2p
Hsk3p
Hsk3p
Spc34p
Hsk3p
Spc19p
Dad2p
Spc34p
Dad4p
Spc34p
c
Ask1p
Spc19p
Ask1p
Spc34p
Dad2p
Duo1p
Dad3p
Duo1p
Duo1p
Spc19p
Legend:
= +MTs
= -MTs
= +/-MTs
1 50 100 150 200 250 300 350 400 450 500 550 600 650
Relative protein position (amino acid residue)

Supplementary Figure 3: Dam1 complex cross-links observed in the presence and absence of microtubules. (a) Protein pairs with multiple cross-links detected in the presence and absence of microtubules. (b) Protein pairs exhibiting minimal cross-links. (c) Protein pairs showing different cross-links in the presence and absence of microtubules. Peptide sequence coverage (colored boxes), mono-links (colored vertical lines and circles) and lysines (vertical white lines) are also shown. Red lines represent cross-links found only in the presence of microtubules. Blue lines represent cross-links found only in the absence of microtubules. Green lines represent cross-links found both in the presence and absence of microtubules. Data is shown for peptides with Percolator[1] assigned *q* values <= 0.01.

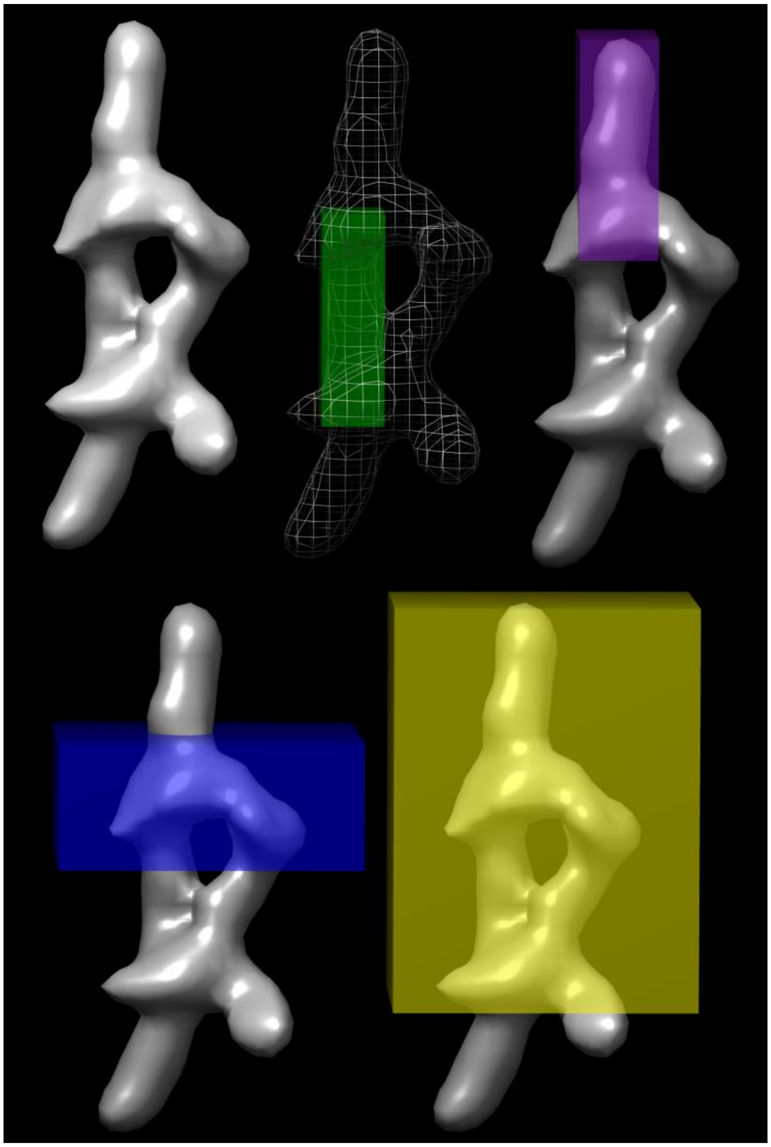

Supplementary Figure 4: Previously published EM localization data was converted into bounding boxes, which were used as restraints during modeling. For orientation, the Dam1 EM structure (EMDB #1373) is shown top left in grey (not used in the modeling). Ask1p/Spc19p (green), Dam1p/Duo1p (blue), Spc34p (purple) and "no localization" (gold) boxes are shown. Boxes apply to a single Dam1 monomer (the upper portion of the structure depicted). The boxes used were purposefully larger than the EM localization data to account for the location of whole proteins rather than the individual protein termini that were localized in the published study. Models run using localization data made use of the blue, green and purple boxes to constrain their respective proteins. All other proteins were constrained to the gold box. We did not apply a hard constraint, but allowed each component to extend beyond its pre-assigned bounding box to satisfy our cross-linking data. This was implemented by using harmonic restraints on the distance between the center of each bead and the wall of the bounding box, acting only when a bead exited the box. In principle, each restrained Dam1 component can extend outside its bounding box by any given amount, provided that the violation of the bounding box restraint is balanced by the satisfaction of a sufficient number of cross-linking restraints.

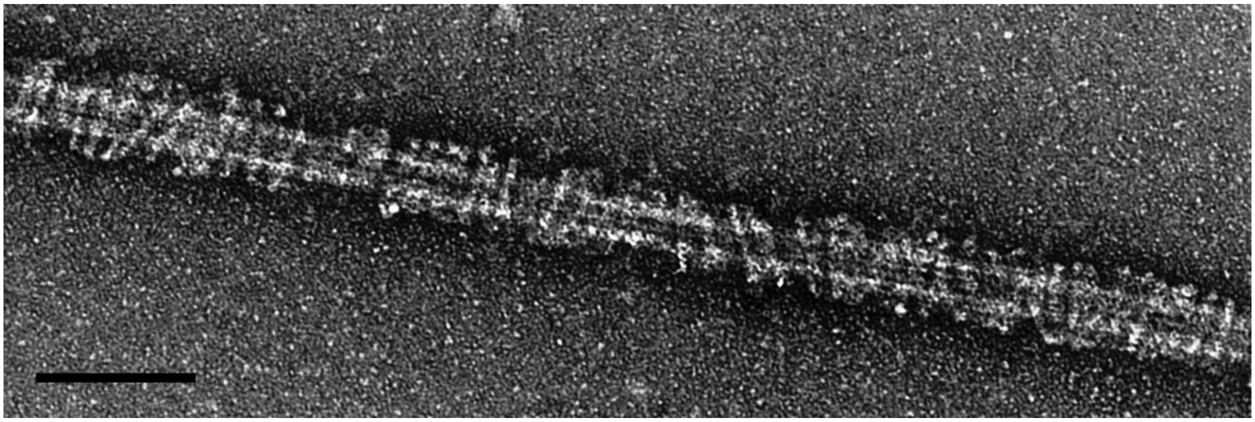

Supplementary Figure 5: Negative stain EM image of Dam1 complex cross-linked to microtubules. Scale bar is 100 nm.

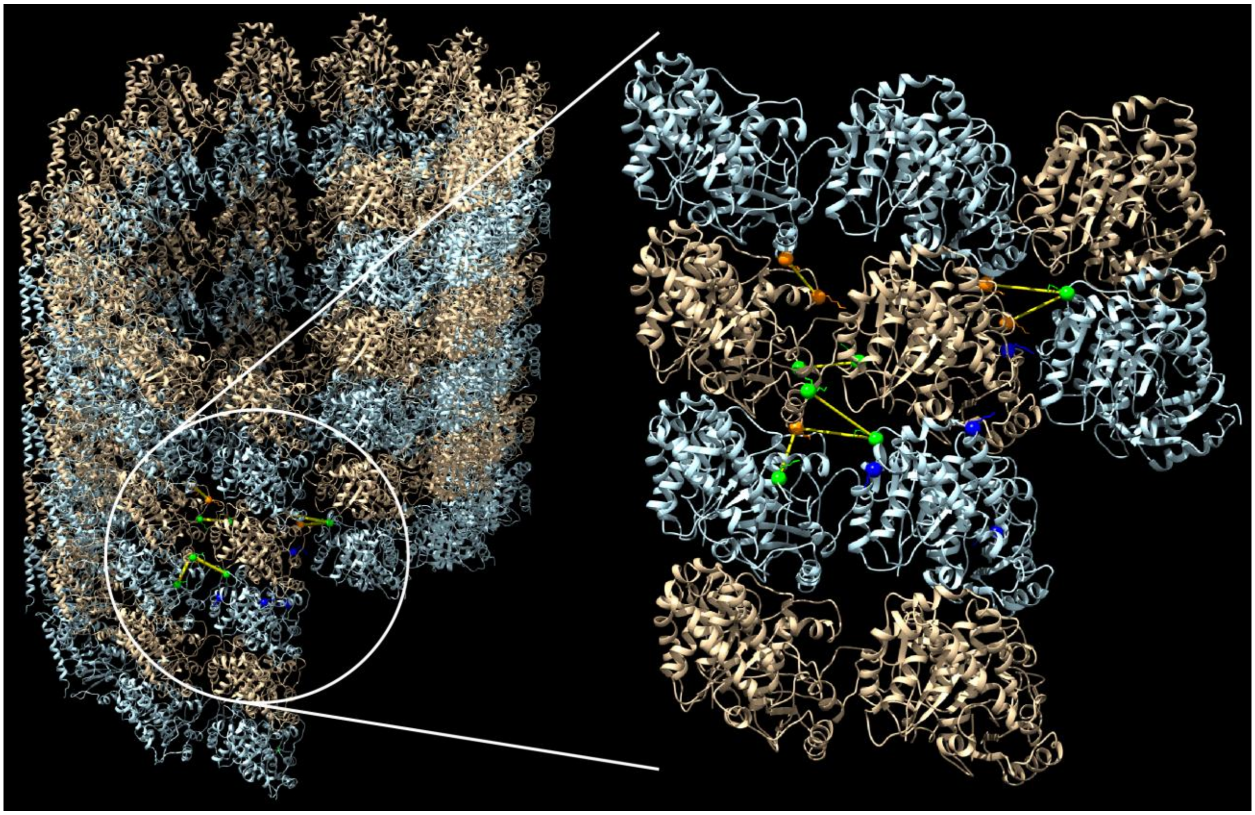

Supplementary Figure 6: Pseudo-atomic structure of a microtubule with tubulin to tubulin cross-links depicted in yellow. All cross-linked Cα-Cα distance are less than 30 Å. Cross-linked lysine α-carbons are depicted as spheres. Green spheres were observed in tubulin to tubulin cross-links only. Blue spheres were observed in tubulin to Dam1 complex cross-links only. Orange spheres were observed in both tubulin to tubulin cross-links and tubulin to Dam1 complex cross-links. Alpha-tubulin is colored light blue. Beta-tubulin is colored copper. Data is shown for peptides with Percolator[1] assigned *q* values <= 0.01.

Supplementary Tables:

Supplementary Table 1: The number and structural agreement of unique distance restraints identified in three protein complexes used to benchmark Kojak.

Complex name	~Size (kDa)	Num. of subunits in complex	Number of linkable sites*	Linkable sites observed modified by cross-linker	Number of unique distance restraint identifications	Number of identifications present in pdb structure	Number with Cα-Cα distance within 30 Å	Agreement with structure
γ-TuSC†	300	4	132	69%	129	70	64	91%
BRCA1/BARD 1†	27	2	25	88%	57	31	25	81%
SCF(FBXL3)†	120	3	66	85%	67	51	48	94%
Dam1c (minus MTs)	200	10	154	94%	678	n/a	n/a	n/a
Dam1c (plus MTs)	200	10	154	90%	458	n/a	n/a	n/a
Dam1c (plus & minus MT combined)	200	10	154	94%	814	n/a	n/a	n/a

*Theoretical linkable sites include all lysines plus the N-terminal amino acid of each protein; †Unlabeled data only; MT = microtubule; Dam1c = Dam1 complex.

Supplementary Table 2: Unique distance constraints within the γ-TuSC found at 1% FDR. Source data for Supplementary Figure 1a.

Type	Protein 1	Pos	Protein 2	Pos	Num PSMs	Num Peptides	Num Unique Peptides	Best Peptide Q-Value	Cα-Cα distance if available
XL	Spc97p	24	Spc97p	39	5	1	1	0.002	-
XL	Spc97p	24	Spc97p	125	4	1	1	0.001	-
XL	Spc97p	37	Spc97p	45	6	1	1	0.002	-
XL	Spc97p	37	Spc97p	93	3	1	1	0.002	-
XL	Spc97p	37	Spc97p	97	4	1	1	0.001	-
XL	Spc97p	39	Spc97p	93	5	1	1	0.001	-
XL	Spc97p	39	Spc97p	97	11	1	1	0.001	-
XL	Spc97p	45	Spc97p	93	3	1	1	0.002	-
XL	Spc97p	45	Spc97p	97	12	1	1	0.002	-
XL	Spc97p	45	Spc97p	103	6	1	1	0.006	-
XL	Spc97p	45	Spc98p	103	7	1	1	0.001	-
XL	Spc97p	45	Spc98p	107	30	1	1	0	-
XL	Spc97p	93	Spc97p	97	11	1	1	0.002	-
XL	Spc97p	93	Spc97p	103	9	1	1	0.002	-
XL	Spc97p	93	Spc97p	114	14	1	1	0	-
XL	Spc97p	97	Spc98p	103	2	1	1	0.009	-
XL	Spc97p	103	Spc97p	114	6	1	1	0.001	-
XL	Spc97p	103	Spc97p	125	1	1	1	0.001	-
XL	Spc97p	114	Spc97p	125	1	1	1	0.001	-
XL	Spc97p	114	Tub4p	370	3	1	1	0.005	-
XL	Spc97p	164	Spc98p	40	3	1	1	0.002	-
XL	Spc97p	164	Spc98p	71	8	1	1	0	-
XL	Spc97p	164	Spc98p	107	26	1	1	0	-
XL	Spc97p	221	Spc97p	328	7	1	1	0	-
XL	Spc97p	221	Spc97p	341	13	1	1	0	-
XL	Spc97p	221	Spc97p	346	1	1	1	0.002	-
XL	Spc97p	221	Spc97p	537	5	1	1	0.002	-
XL	Spc97p	221	Spc97p	581	2	1	1	0.002	-
XL*	Spc97p	328	Spc97p	341	16	1	1	0.001	17
XL	Spc97p	328	Spc97p	346	6	1	1	0.001	19

XL	Spc97p	328	Spc97p	355	3	1	1	0.001	20
XL	Spc97p	355	Spc98p	709	9	1	1	0.003	-
XL	Spc97p	355	Spc98p	712	9	1	1	0.002	34
XL	Spc97p	441	Spc97p	460	9	1	1	0	12
XL	Spc97p	441	Tub4p	84	3	1	1	0.001	20
XL	Spc97p	441	Tub4p	198	14	1	1	0.001	18
XL	Spc97p	441	Tub4p	370	7	1	1	0.001	17
XL	Spc97p	448	Spc97p	460	13	1	1	0	16
XL	Spc97p	448	Spc97p	537	6	1	1	0.001	-
XL	Spc97p	448	Spc97p	708	2	1	1	0.001	17
XL	Spc97p	448	Spc97p	711	2	1	1	0.009	22
XL	Spc97p	448	Spc98p	843	5	2	2	0.001	23
XL	Spc97p	448	Tub4p	370	11	1	1	0.001	28
XL	Spc97p	460	Spc97p	537	1	1	1	0.001	-
XL	Spc97p	460	Spc98p	831	5	1	1	0	9
XL	Spc97p	460	Spc98p	843	12	2	2	0	17
XL	Spc97p	460	Tub4p	198	1	1	1	0.002	29
XL	Spc97p	460	Tub4p	370	15	2	2	0	20
XL	Spc97p	537	Spc97p	554	1	1	1	0.007	-
XL	Spc97p	537	Spc97p	708	3	1	1	0.001	-
XL	Spc97p	537	Spc97p	716	9	1	1	0	-
XL	Spc97p	537	Spc97p	734	3	1	1	0.001	-
XL	Spc97p	537	Spc97p	777	3	1	1	0	-
XL	Spc97p	537	Spc98p	831	7	1	1	0.001	-
XL	Spc97p	537	Spc98p	843	4	2	2	0.001	-
XL	Spc97p	537	Tub4p	370	8	1	1	0.001	-
XL	Spc97p	635	Spc97p	790	1	1	1	0	11
XL	Spc97p	708	Spc97p	716	10	1	1	0.001	14
XL	Spc97p	711	Spc97p	716	4	1	1	0.002	11
XL	Spc97p	716	Spc97p	734	11	1	1	0	-
XL	Spc97p	727	Spc97p	734	10	1	1	0.001	-
XL	Spc97p	734	Tub4p	197	3	2	2	0	-
XL	Spc97p	734	Tub4p	198	4	1	1	0.001	-
XL	Spc97p	734	Tub4p	248	1	1	1	0	-
XL	Spc97p	734	Tub4p	370	5	1	1	0.005	-
XL	Spc97p	734	Tub4p	376	2	1	1	0.002	-

XL	Spc97p	790	Tub4p	370	5	1	1	0	25
XL	Spc98p	68	Spc98p	103	11	1	1	0	-
XL	Spc98p	71	Spc98p	103	8	1	1	0	-
XL	Spc98p	71	Spc98p	107	4	1	1	0	-
XL	Spc98p	103	Spc98p	103	1	1	1	0.001	-
XL	Spc98p	401	Spc98p	674	5	1	1	0.001	16
XL	Spc98p	401	Spc98p	704	1	1	1	0.005	-
XL	Spc98p	401	Spc98p	709	2	2	2	0.007	-
XL	Spc98p	428	Spc98p	674	2	1	1	0.001	27
XL	Spc98p	428	Spc98p	709	8	1	1	0.001	-
XL	Spc98p	495	Tub4p	198	2	1	1	0	21
XL	Spc98p	495	Tub4p	370	3	1	1	0.001	19
XL	Spc98p	511	Tub4p	370	1	1	1	0.002	23
XL	Spc98p	602	Tub4p	198	5	1	1	0.001	17
XL	Spc98p	630	Spc98p	810	6	1	1	0	12
XL	Spc98p	647	Spc98p	820	6	1	1	0.001	15
XL	Spc98p	647	Tub4p	370	2	1	1	0.002	15
XL	Spc98p	662	Spc98p	674	3	1	1	0	13
XL	Spc98p	674	Spc98p	698	6	1	1	0	-
XL	Spc98p	674	Spc98p	704	13	1	1	0	-
XL	Spc98p	674	Spc98p	709	8	2	2	0.001	-
XL	Spc98p	674	Spc98p	712	7	1	1	0.001	13
XL	Spc98p	674	Spc98p	717	6	1	1	0	5
XL	Spc98p	698	Spc98p	709	8	1	1	0.001	-
XL*	Spc98p	704	Spc98p	712	5	1	1	0.006	-
XL*	Spc98p	709	Spc98p	717	1	1	1	0.001	-
XL	Spc98p	831	Tub4p	370	1	1	1	0.002	11
XL	Spc98p	843	Tub4p	370	25	2	2	0	28
XL	Spc98p	843	Tub4p	376	13	2	2	0	33
XL	Tub4p	84	Tub4p	94	9	2	2	0.002	11
XL	Tub4p	84	Tub4p	370	5	2	2	0.005	22
XL	Tub4p	94	Tub4p	376	1	1	1	0	36
XL	Tub4p	198	Tub4p	370	4	1	1	0	19
XL	Tub4p	198	Tub4p	376	1	1	1	0.002	26
XL	Tub4p	248	Tub4p	370	1	1	1	0	30
XL	Tub4p	370	Tub4p	376	20	1	1	0	12

XL	Tub4p	370	Tub4p	433	3	1	1	0	50
LL	Spc97p	24	Spc97p	37	2	1	1	0.002	-
LL	Spc97p	37	Spc97p	39	11	1	1	0	-
LL	Spc97p	39	Spc97p	45	7	1	1	0.002	-
LL	Spc97p	93	Spc97p	96	16	1	1	0	10
LL	Spc97p	96	Spc97p	97	7	2	2	0.001	4
LL	Spc97p	249	Spc97p	254	4	1	1	0.001	8
LL*	Spc97p	328	Spc97p	341	2	1	1	0.008	17
LL	Spc97p	341	Spc97p	346	2	1	1	0.001	10
LL	Spc97p	441	Spc97p	448	1	1	1	0.009	14
LL	Spc97p	608	Spc97p	613	4	1	1	0	8
LL	Spc97p	654	Spc97p	661	17	1	1	0	10
LL	Spc97p	729	Spc97p	734	6	1	1	0.001	-
LL	Spc97p	777	Spc97p	790	9	1	1	0	19
LL	Spc98p	103	Spc98p	107	16	1	1	0	-
LL	Spc98p	396	Spc98p	401	3	1	1	0.001	9
LL	Spc98p	414	Spc98p	417	15	2	2	0	5
LL	Spc98p	427	Spc98p	428	3	1	1	0	4
LL	Spc98p	495	Spc98p	511	5	1	1	0.001	9
LL	Spc98p	698	Spc98p	704	15	2	2	0	-
LL	Spc98p	704	Spc98p	709	20	1	1	0.001	-
LL*	Spc98p	704	Spc98p	712	1	1	1	0.006	-
LL	Spc98p	709	Spc98p	712	19	2	2	0	-
LL*	Spc98p	709	Spc98p	717	1	1	1	0.002	-
LL	Spc98p	712	Spc98p	717	1	1	1	0.002	9
LL	Spc98p	804	Spc98p	810	2	1	1	0.001	10
LL	Spc98p	810	Spc98p	817	1	1	1	0.005	9
LL	Tub4p	60	Tub4p	84	1	1	1	0.005	13
LL	Tub4p	155	Tub4p	158	9	3	3	0	5
LL	Tub4p	197	Tub4p	198	34	3	3	0	4
**XL=cross-link; LL=loop-link; *Identified as both a XL and a LL**									

Supplementary Table 3: BRCA1/BARD1 unique distance constraints found at 1% FDR. Source data for Supplementary Figure 1b.

Type	Protein 1	Pos	Protein 2	Pos	Num PSMs	Num Peptides	Num Unique Peptides	Best Peptide Q-Value	Cα-Cα distance if available
XL	BRCA1	40	BRCA1	85	1	1	1	0.01	20
XL	BRCA1	40	BARD1	85	13	2	2	0	15
XL	BRCA1	40	BARD1	99	7	1	1	0.002	-
XL	BRCA1	40	BARD1	102	2	2	1	0.004	-
XL	BRCA1	40	BARD1	105	11	4	4	0	-
XL	BRCA1	52	BRCA1	58	5	1	1	0.005	19
XL	BRCA1	52	BRCA1	70	12	1	1	0	16
XL	BRCA1	52	BRCA1	75	18	1	0	0.002	24
XL	BRCA1	52	BRCA1	76	5	1	1	0.002	26
XL	BRCA1	52	BRCA1	90	3	1	1	0.002	20
XL	BRCA1	52	BRCA1	129	4	1	1	0.002	-
XL	BRCA1	52	BARD1	1	2	1	1	0.008	42
XL	BRCA1	52	BARD1	21	3	1	0	0.003	18
XL	BRCA1	58	BRCA1	76	6	2	2	0.01	18
XL	BRCA1	58	BRCA1	85	8	2	2	0.005	12
XL	BRCA1	58	BRCA1	90	6	1	1	0.01	10
XL	BRCA1	58	BARD1	71	4	1	1	0.003	12
XL	BRCA1	65	BRCA1	90	4	1	1	0.005	11
XL	BRCA1	70	BRCA1	76	33	1	1	0.003	11
XL	BRCA1	70	BRCA1	85	6	1	1	0.005	16
XL	BRCA1	70	BRCA1	129	4	1	1	0	-
XL	BRCA1	70	BARD1	1	8	1	1	0.005	56
XL	BRCA1	70	BARD1	105	5	1	1	0.01	-
XL*	BRCA1	75	BRCA1	76	5	1	0	0.009	4
XL*	BRCA1	75	BRCA1	85	7	2	0	0.005	14
XL	BRCA1	75	BARD1	21	2	1	0	0.01	39
XL	BRCA1	76	BRCA1	85	7	1	1	0.004	15
XL	BRCA1	76	BRCA1	90	27	1	1	0.003	12
XL	BRCA1	76	BARD1	1	8	1	1	0.005	66
XL	BRCA1	76	BARD1	21	9	1	0	0.003	41

XL	BRCA1	76	BARD1	85	3	1	1	0.005	44
XL*	BRCA1	85	BRCA1	90	1	1	1	0.006	16
XL	BRCA1	85	BRCA1	129	1	1	1	0.005	-
XL	BRCA1	90	BARD1	115	1	1	1	0.005	-
XL	BARD1	1	BARD1	85	5	1	1	0.005	30
XL	BARD1	1	BARD1	99	19	1	1	0.002	-
XL	BARD1	1	BARD1	102	18	1	1	0	-
XL	BARD1	1	BARD1	105	28	2	2	0.004	-
XL	BARD1	1	BARD1	115	5	2	2	0.005	-
XL	BARD1	21	BARD1	85	4	1	0	0.005	16
XL	BARD1	21	BARD1	99	3	1	0	0.003	-
XL	BARD1	21	BARD1	105	41	2	0	0.002	-
XL	BARD1	21	BARD1	115	6	1	0	0.003	-
XL	BARD1	85	BARD1	99	5	1	1	0.005	-
XL	BARD1	85	BARD1	102	3	1	1	0.005	-
XL	BARD1	85	BARD1	105	24	2	2	0.003	-
XL	BARD1	85	BARD1	114	1	1	1	0.004	-
XL*	BARD1	99	BARD1	105	47	3	3	0	-
XL	BARD1	99	BARD1	115	1	1	1	0.003	-
XL*	BARD1	102	BARD1	105	53	4	3	0	-
XL	BARD1	102	BARD1	115	3	2	2	0	-
LL	BRCA1	40	BRCA1	52	3	1	1	0.006	14
LL	BRCA1	65	BRCA1	70	5	1	1	0.005	9
LL	BRCA1	70	BRCA1	75	5	2	2	0.005	9
LL*	BRCA1	75	BRCA1	76	16	1	1	0.003	4
LL*	BRCA1	75	BRCA1	85	3	1	1	0.005	14
LL*	BRCA1	85	BRCA1	90	9	2	2	0.005	16
LL	BRCA1	129	BRCA1	130	13	1	1	0	-
LL	BARD1	99	BARD1	102	76	3	3	0	-
LL*	BARD1	99	BARD1	105	9	1	1	0.004	-
LL*	BARD1	102	BARD1	105	5	2	2	0.005	-
LL	BARD1	114	BARD1	115	8	1	1	0.005	-

XL=cross-link; LL=loop-link; *Identified as both a XL and a LL

Supplementary Table 4: SCF^{FBXL3} unique distance constraints found at 1% FDR. Source data for Supplementary Figure 1c.

Type	Protein 1	Pos	Protein 2	Pos	Num PSMs	Num Peptides	Num Unique Peptides	Best Peptide Q-Value	Cα-Cα distance if available
XL	Skp1	51	Fbxl3	22	12	1	1	0.005	-
XL	Skp1	80	Skp1	116	3	1	0	0.008	20
XL	Skp1	114	Skp1	149	30	2	0	0.005	-
XL	Skp1	114	Fbxl3	22	7	1	0	0.005	-
XL*	Skp1	116	Skp1	123	23	2	0	0.005	10
XL	Skp1	116	Skp1	128	118	4	0	0.001	16
XL	Skp1	116	Fbxl3	22	23	2	0	0.005	-
XL	Skp1	123	Skp1	149	33	2	0	0.005	-
XL	Skp1	123	Fbxl3	22	41	3	0	0.001	-
XL	Skp1	128	Skp1	141	6	1	1	0.001	21
XL	Skp1	128	Skp1	149	5	1	1	0.003	-
XL	Skp1	128	Fbxl3	22	34	5	5	0.001	-
XL	Skp1	141	Fbxl3	94	24	1	1	0.001	18
XL	Skp1	141	Fbxl3	102	61	1	1	0.005	14
XL	Skp1	141	Fbxl3	106	21	1	1	0.005	17
XL	Skp1	149	Fbxl3	94	52	2	2	0.001	-
XL	Skp1	149	Fbxl3	102	44	2	2	0.005	-
XL	Skp1	149	Fbxl3	106	6	1	1	0.008	-
XL	Fbxl3	118	Fbxl3	180	814	2	2	0.001	11
XL	Fbxl3	142	Fbxl3	180	10	1	1	0.001	12
XL	Fbxl3	142	Fbxl3	203	5	1	1	0.001	10
XL	Fbxl3	174	Fbxl3	192	4	1	1	0.001	14
XL*	Fbxl3	180	Fbxl3	192	5	1	1	0.008	16
XL	Fbxl3	180	Fbxl3	203	50	1	1	0.001	11
XL	Fbxl3	180	Fbxl3	206	203	1	1	0.001	5
XL	Fbxl3	180	mCRY2	163	18	1	1	0.005	40
XL	Fbxl3	180	mCRY2	503	14	1	1	0.003	15
XL	Fbxl3	192	mCRY2	163	46	1	1	0.001	57
XL	Fbxl3	192	mCRY2	503	10	1	1	0.001	30
XL	Fbxl3	203	Fbxl3	250	4	1	1	0.002	15

XL	Fbxl3	203	mCRY2	503	9	1	1	0.005	21
XL	mCRY2	2	mCRY2	86	21	1	1	0.001	-
XL	mCRY2	2	mCRY2	107	25	1	1	0	-
XL	mCRY2	2	mCRY2	133	8	1	1	0.002	-
XL	mCRY2	2	mCRY2	182	1	1	1	0.005	-
XL	mCRY2	2	mCRY2	183	32	2	2	0	-
XL	mCRY2	107	mCRY2	133	27	1	1	0.003	16
XL	mCRY2	107	mCRY2	136	12	1	1	0.001	14
XL	mCRY2	107	mCRY2	183	5	1	1	0.005	37
XL	mCRY2	125	mCRY2	136	27	1	1	0.001	16
XL	mCRY2	183	mCRY2	292	315	3	3	0.001	16
XL*	mCRY2	241	mCRY2	246	468	2	2	0.001	10
XL	mCRY2	453	mCRY2	474	21	1	1	0.002	12
XL	mCRY2	453	mCRY2	477	24	2	2	0.008	8
XL	mCRY2	455	mCRY2	477	2476	3	3	0.001	9
LL	Skp1	22	Skp1	28	4	2	2	0	10
LL	Skp1	50	Skp1	51	13	1	1	0	4
LL	Skp1	107	Skp1	114	2	1	1	0.005	11
LL	Skp1	114	Skp1	116	55	2	0	0.001	6
LL*	Skp1	116	Skp1	123	1	1	1	0.006	10
LL	Skp1	123	Skp1	128	35	2	2	0.001	9
LL	Fbxl3	22	Fbxl3	24	47	2	2	0.001	-
LL	Fbxl3	22	Fbxl3	25	19	2	2	0.001	-
LL	Fbxl3	24	Fbxl3	25	9	1	1	0.001	-
LL	Fbxl3	94	Fbxl3	102	8	1	1	0.001	17
LL	Fbxl3	102	Fbxl3	106	56	1	1	0.005	6
LL	Fbxl3	174	Fbxl3	180	2	1	1	0.002	17
LL*	Fbxl3	180	Fbxl3	192	2	1	1	0.005	16
LL	Fbxl3	192	Fbxl3	203	7	1	1	0.001	16
LL	Fbxl3	203	Fbxl3	206	21	1	1	0	10
LL	Fbxl3	404	Fbxl3	416	42	1	1	0.001	17
LL	mCRY2	133	mCRY2	136	138	2	2	0	5
LL	mCRY2	163	mCRY2	169	39	1	1	0.003	15
LL	mCRY2	182	mCRY2	183	77	2	2	0.001	4
LL*	mCRY2	241	mCRY2	246	21	1	1	0.001	10
LL	mCRY2	292	mCRY2	293	7	1	1	0.007	4

LL	mCRY2	292	mCRY2	295	6	1	1	0.005	6
LL	mCRY2	293	mCRY2	295	2	1	1	0.003	6
LL	mCRY2	453	mCRY2	455	67	2	2	0.005	6
LL	mCRY2	474	mCRY2	477	78	2	2	0	5
**XL=cross-link; LL=loop-link; *Identified as both a XL and a LL**									

Supplementary Table 5: Comparison of the number of unique cross-link identifications from various published cross-linking methods applied to a range of protein complexes. Results obtained using Kojak are shaded lilac.

Complex Name	~ Size (kDa)	Num. of Subunits in complex	Number of unique cross-links	Number of unique cross-links per kDa protein	FDR	Isotope Labeling	Post cross-linking enrichment or fractionation	Reference
TRiC Bovine	1000	16	73 to 189*	0.07 to 0.19	5%	yes[1]	SEC	[2]
TRiC Yeast	1000	16	206 to 226†	0.21 to 0.23	5%	yes[1]	SEC	[2]
GST homodimer	50	2	8	0.16	5%	yes[2]	n/a	[3]
CNGP	80	4	15	0.18	5%	no[3]	n/a	[3]
yeast UTP-B	550	6	71	0.13	5%	yes[2]	n/a	[3]
RNA polymerase II	510	12	106	0.21	n/a	no[3]	SCX	[4]
As above + TFIIF complex	670	15	402	0.60	n/a	no[3]	SCX	[4]
RNA polymerase I	590	14	239	0.41	n/a	yes[1]	SEC	[5]
$\text{SCF}^{\text{FBXL3}}$	130	3	91	0.70	1%	no[4]	no	[6]
Ndc80 complex	185	4	277	1.50	5%	no[4]	no	[7]
BARD1/BRCA1	27	2	75	2.78	5%	no[4]	no	this study
$\text{SCF}^{\text{FBXL3}}$	130	3	89	0.68	5%	no[4]	no	this study
γ-TuSC	300	3	111	0.37	5%	no[4]	no	this study
Dam1 complex	200	10	618	3.09	5%	no[4]	no	this study

SEC = size exclusion chromatography; SCX = strong cation exchange chromatography; *TRiC was cross-linked in the apo, ATP, and ATP+AlFx states; †TRiC was cross-linked in the apo and ATP+AlFx states.
[1]DSS-d0/d12; [2]BS3-d0/d4; [3]BS3; [4]DSS

Supplementary Table 6: Unique distance constraints within the Dam1p complex found at 1% FDR and cross-linked in the presence (+) or absence (-) of microtubules (MTs).

Type	Protein 1	Pos	Protein 2	Pos	Num PSMs	Num Peptides	Num Unique Peptides	Best Peptide Q-Value	Identified Cross-linked +/- MTs
XL*	Ask1p	1	Ask1p	12	33	1	1	0	+/-
XL	Ask1p	1	Ask1p	260	1	1	1	0.002	-
XL	Ask1p	1	Dad2p	1	16	3	3	0	-
XL	Ask1p	1	Spc34p	274	3	1	1	0.007	+
XL	Ask1p	6	Dad2p	1	3	1	1	0.003	-
XL	Ask1p	151	Ask1p	198	9	1	1	0	+/-
XL	Ask1p	151	Ask1p	222	2	1	1	0	-
XL	Ask1p	151	Ask1p	225	15	1	1	0	+/-
XL	Ask1p	151	Dad2p	1	3	1	1	0.001	+
XL	Ask1p	151	Spc34p	2	6	1	1	0	+
XL	Ask1p	151	Spc34p	274	2	1	1	0.002	+
XL	Ask1p	198	Ask1p	225	58	1	1	0	+/-
XL	Ask1p	198	Ask1p	253	1	1	1	0.001	-
XL	Ask1p	198	Ask1p	260	2	1	1	0.002	+/-
XL	Ask1p	198	Spc34p	2	2	1	1	0	+
XL	Ask1p	198	Spc34p	249	9	1	1	0	+
XL*	Ask1p	222	Ask1p	225	47	3	3	0	+/-
XL	Ask1p	222	Ask1p	260	6	1	1	0	+/-
XL	Ask1p	225	Ask1p	225	1	1	1	0	+
XL	Ask1p	225	Ask1p	260	63	1	1	0	+/-
XL	Ask1p	225	Ask1p	277	9	1	1	0	+/-
XL	Ask1p	225	Ask1p	281	24	1	1	0	+/-
XL	Ask1p	225	Spc34p	249	2	1	1	0.001	+
XL	Ask1p	253	Ask1p	281	16	2	2	0.001	-
XL*	Ask1p	260	Ask1p	281	23	1	1	0	+/-
XL	Dad1p	1	Ask1p	198	8	1	1	0	+
XL	Dad1p	1	Ask1p	222	1	1	1	0.002	-
XL	Dad1p	1	Ask1p	225	11	1	1	0	+
XL	Dad1p	1	Ask1p	253	2	2	2	0	+/-
XL	Dad1p	1	Ask1p	260	4	1	1	0	+/-

XL	Dad1p	1	Ask1p	277	6	1	1	0.005	+
XL	Dad1p	1	Ask1p	281	3	1	1	0.001	+
XL	Dad1p	1	Dad1p	1	13	1	1	0	-
XL*	Dad1p	1	Dad1p	11	11	2	2	0	-
XL	Dad1p	1	Dad1p	18	123	1	1	0	+/-
XL	Dad1p	1	Dad1p	67	1	1	1	0	-
XL	Dad1p	1	Dad1p	92	24	2	2	0	+/-
XL	Dad1p	1	Dad1p	94	16	2	2	0	+/-
XL	Dad1p	1	Dad2p	1	3	1	1	0	-
XL	Dad1p	1	Dad2p	11	21	1	1	0	+/-
XL	Dad1p	1	Dad2p	13	10	1	1	0.001	+/-
XL	Dad1p	1	Dad2p	20	4	1	1	0	-
XL	Dad1p	1	Dad4p	1	48	2	2	0	+/-
XL	Dad1p	1	Dad4p	21	10	1	1	0.001	+/-
XL	Dad1p	1	Dam1p	7	24	2	2	0.001	+/-
XL	Dad1p	1	Dam1p	40	65	3	3	0	+/-
XL	Dad1p	1	Dam1p	66	22	1	1	0	+/-
XL	Dad1p	1	Dam1p	256	5	1	1	0	-
XL	Dad1p	1	Dam1p	307	12	1	1	0	+/-
XL	Dad1p	1	Duo1p	14	7	1	1	0.005	-
XL	Dad1p	1	Duo1p	33	46	1	1	0	+/-
XL	Dad1p	1	Duo1p	36	74	1	1	0	+/-
XL	Dad1p	1	Duo1p	66	65	3	3	0	+/-
XL	Dad1p	1	Duo1p	73	9	1	1	0	-
XL	Dad1p	1	Duo1p	86	3	1	1	0.001	-
XL	Dad1p	1	Duo1p	101	1	1	1	0.005	-
XL	Dad1p	1	Duo1p	122	4	1	1	0.001	-
XL	Dad1p	1	Duo1p	131	4	1	1	0	-
XL	Dad1p	1	Duo1p	159	4	1	1	0	-
XL	Dad1p	1	Duo1p	160	4	1	1	0.005	-
XL	Dad1p	1	Duo1p	169	11	1	1	0.001	+/-
XL	Dad1p	1	Duo1p	177	8	1	1	0.004	+/-
XL	Dad1p	1	Duo1p	215	9	1	1	0.002	-
XL	Dad1p	1	Duo1p	236	14	1	1	0	+/-
XL	Dad1p	1	Hsk3p	5	12	1	1	0	-
XL	Dad1p	1	Hsk3p	28	1	1	1	0.001	-

XL	Dad1p	1	Spc19p	1	2	1	1	0	-
XL	Dad1p	1	Spc19p	59	15	1	1	0	-
XL	Dad1p	1	Spc19p	76	17	1	1	0.001	+/-
XL	Dad1p	1	Spc19p	104	11	1	1	0	+/-
XL	Dad1p	1	Spc19p	146	2	1	1	0.004	-
XL	Dad1p	1	Spc19p	158	10	1	1	0	+
XL	Dad1p	1	Spc34p	1	2	1	1	0.001	-
XL	Dad1p	1	Spc34p	2	19	1	1	0	+/-
XL	Dad1p	1	Spc34p	46	2	1	1	0.006	+/-
XL	Dad1p	1	Spc34p	70	9	1	1	0	+/-
XL	Dad1p	1	Spc34p	79	1	1	1	0.003	-
XL	Dad1p	1	Spc34p	88	4	1	1	0	+
XL	Dad1p	1	Spc34p	112	43	4	4	0	+/-
XL	Dad1p	1	Spc34p	118	23	1	1	0	+/-
XL	Dad1p	1	Spc34p	126	7	1	1	0.002	+/-
XL	Dad1p	1	Spc34p	198	7	1	1	0.001	+/-
XL	Dad1p	1	Spc34p	243	15	1	1	0	+/-
XL	Dad1p	1	Spc34p	249	13	1	1	0	+/-
XL	Dad1p	1	Spc34p	274	8	1	1	0	+
XL	Dad1p	2	Dad4p	2	8	1	1	0	-
XL	Dad1p	2	Dam1p	1	6	1	0	0.001	+/-
XL	Dad1p	11	Dad2p	11	38	2	2	0	+/-
XL	Dad1p	11	Dad4p	1	29	2	2	0	+/-
XL	Dad1p	11	Dam1p	40	1	1	1	0	+
XL	Dad1p	11	Duo1p	33	11	2	2	0.001	+/-
XL	Dad1p	11	Duo1p	36	5	2	2	0.003	+/-
XL	Dad1p	11	Hsk3p	5	4	1	1	0.003	-
XL	Dad1p	11	Spc34p	112	1	1	1	0.004	+
XL	Dad1p	18	Dad1p	18	1	1	1	0.003	-
XL	Dad1p	18	Dad1p	92	14	1	1	0.001	+/-
XL	Dad1p	18	Dad1p	94	8	1	1	0	+/-
XL	Dad1p	18	Dad2p	11	12	1	1	0.001	-
XL	Dad1p	18	Dad4p	1	8	1	1	0	+/-
XL	Dad1p	18	Dam1p	7	2	1	1	0.004	-
XL	Dad1p	18	Dam1p	40	14	1	1	0.001	+/-
XL	Dad1p	18	Duo1p	33	28	2	2	0	+/-

XL	Dad1p	18	Duo1p	36	77	1	1	0	+/-
XL	Dad1p	18	Duo1p	169	3	1	1	0.007	-
XL	Dad1p	18	Duo1p	236	4	1	1	0.005	-
XL	Dad1p	18	Spc19p	44	2	1	1	0.002	-
XL	Dad1p	18	Spc19p	59	3	1	1	0.002	-
XL	Dad1p	18	Spc34p	88	20	1	1	0	+/-
XL	Dad1p	18	Spc34p	112	10	1	1	0	+
XL	Dad1p	18	Spc34p	249	3	1	1	0.002	+/-
XL	Dad1p	92	Ask1p	151	2	1	1	0	+
XL	Dad1p	92	Ask1p	225	2	1	1	0.005	+
XL	Dad1p	92	Dad4p	1	4	1	1	0.004	+/-
XL	Dad1p	92	Duo1p	36	10	1	1	0	+
XL	Dad1p	92	Spc34p	112	4	1	1	0	+/-
XL	Dad1p	92	Spc34p	249	3	1	1	0.002	+
XL	Dad1p	94	Ask1p	225	1	1	1	0.005	+
XL	Dad1p	94	Dad1p	94	1	1	1	0.001	-
XL	Dad1p	94	Dad4p	1	13	1	1	0	+/-
XL	Dad1p	94	Duo1p	36	1	1	1	0.001	+
XL	Dad1p	94	Spc34p	112	3	1	1	0.003	-
XL	Dad2p	1	Dad2p	13	22	2	2	0	+/-
XL	Dad2p	11	Dad2p	20	7	1	1	0.001	-
XL	Dad2p	13	Dad2p	29	6	1	1	0.001	-
XL	Dad2p	88	Spc34p	41	1	1	1	0.001	-
XL	Dad2p	106	Spc34p	41	3	1	1	0	-
XL	Dad2p	106	Spc34p	46	1	1	1	0.005	-
XL	Dad2p	106	Spc34p	249	4	1	1	0.002	+
XL	Dad3p	1	Ask1p	225	2	1	1	0.001	+
XL	Dad3p	1	Dad1p	1	135	4	4	0	+/-
XL	Dad3p	1	Dad1p	11	87	4	4	0	+/-
XL	Dad3p	1	Dad1p	18	128	3	3	0	+/-
XL	Dad3p	1	Dad1p	92	19	2	2	0	+/-
XL	Dad3p	1	Dad1p	94	29	2	2	0	+/-
XL	Dad3p	1	Dad2p	1	8	2	2	0	-
XL	Dad3p	1	Dad2p	11	10	2	2	0.001	-
XL	Dad3p	1	Dad3p	1	28	3	3	0	+/-
XL*	Dad3p	1	Dad3p	15	8	1	1	0	+/-

XL*	Dad3p	1	Dad3p	17	33	1	1	0	+/-
XL	Dad3p	1	Dad3p	72	8	1	1	0.001	-
XL	Dad3p	1	Dad3p	83	33	2	2	0	+/-
XL	Dad3p	1	Dad3p	94	32	3	3	0	+/-
XL	Dad3p	1	Dad4p	1	49	2	2	0	+/-
XL	Dad3p	1	Dad4p	21	6	1	1	0.001	+/-
XL	Dad3p	1	Dam1p	7	9	2	2	0	-
XL	Dad3p	1	Dam1p	40	30	3	3	0	+/-
XL	Dad3p	1	Duo1p	33	54	3	3	0	+/-
XL	Dad3p	1	Duo1p	36	83	2	2	0	+/-
XL	Dad3p	1	Duo1p	66	7	3	3	0	-
XL	Dad3p	1	Duo1p	73	6	2	2	0	-
XL	Dad3p	1	Duo1p	131	5	1	1	0.001	-
XL	Dad3p	1	Duo1p	161	1	1	1	0	-
XL	Dad3p	1	Duo1p	169	1	1	1	0.006	-
XL	Dad3p	1	Duo1p	177	4	1	1	0.004	-
XL	Dad3p	1	Duo1p	183	2	1	1	0.003	-
XL	Dad3p	1	Duo1p	215	6	1	1	0	-
XL	Dad3p	1	Duo1p	222	2	1	1	0.001	-
XL	Dad3p	1	Hsk3p	5	18	2	2	0	+/-
XL	Dad3p	1	Spc19p	59	3	1	1	0	+/-
XL	Dad3p	1	Spc19p	104	3	2	2	0.002	+/-
XL	Dad3p	1	Spc19p	158	6	1	1	0.001	+/-
XL	Dad3p	1	Spc34p	70	2	1	1	0.001	+
XL	Dad3p	1	Spc34p	79	36	4	4	0	+/-
XL	Dad3p	1	Spc34p	88	67	3	3	0	+/-
XL	Dad3p	1	Spc34p	112	123	5	5	0	+/-
XL	Dad3p	1	Spc34p	118	67	2	2	0	+/-
XL	Dad3p	1	Spc34p	126	24	3	3	0	+/-
XL	Dad3p	1	Spc34p	162	3	1	1	0.004	-
XL	Dad3p	1	Spc34p	169	14	2	2	0	+/-
XL	Dad3p	1	Spc34p	243	1	1	1	0.004	+
XL	Dad3p	1	Spc34p	249	1	1	1	0	+
XL	Dad3p	2	Dad1p	2	34	4	4	0	+/-
XL	Dad3p	2	Dad1p	11	2	1	1	0.004	-
XL	Dad3p	2	Dam1p	1	5	2	0	0	-

XL	Dad3p	15	Dad1p	1	28	2	2	0	+/-
XL	Dad3p	15	Dad1p	11	4	2	2	0.001	-
XL	Dad3p	15	Dad1p	92	2	1	1	0.009	-
XL	Dad3p	15	Dad1p	94	5	1	1	0.003	-
XL	Dad3p	15	Dad2p	1	5	1	1	0	-
XL	Dad3p	15	Dad3p	15	1	1	1	0.004	-
XL*	Dad3p	15	Dad3p	17	11	2	2	0	-
XL	Dad3p	15	Dad3p	72	3	1	1	0	-
XL	Dad3p	15	Dad3p	83	22	1	1	0	-
XL	Dad3p	15	Dad3p	94	18	2	2	0	-
XL	Dad3p	15	Dad4p	1	23	1	1	0	+/-
XL	Dad3p	15	Dad4p	21	18	1	1	0	+/-
XL	Dad3p	15	Spc34p	2	10	1	1	0	+
XL	Dad3p	15	Spc34p	65	3	1	1	0.001	-
XL	Dad3p	15	Spc34p	70	7	1	1	0.002	+
XL	Dad3p	15	Spc34p	88	4	1	1	0.005	+/-
XL	Dad3p	15	Spc34p	169	4	1	1	0.002	+
XL	Dad3p	17	Dad1p	1	24	2	2	0	+/-
XL	Dad3p	17	Dad1p	18	4	1	1	0.005	-
XL	Dad3p	17	Dad1p	92	1	1	1	0.006	-
XL	Dad3p	17	Dad1p	94	1	1	1	0.003	-
XL	Dad3p	17	Dad3p	83	10	1	1	0.001	-
XL	Dad3p	17	Dad4p	1	1	1	1	0.007	-
XL	Dad3p	17	Spc34p	65	6	2	2	0.001	+/-
XL	Dad3p	17	Spc34p	70	20	2	2	0.005	+/-
XL	Dad3p	17	Spc34p	88	9	1	1	0.003	+/-
XL	Dad3p	17	Spc34p	169	14	1	1	0	+/-
XL	Dad3p	24	Dad1p	1	4	1	1	0	-
XL	Dad3p	24	Dad4p	1	1	1	1	0.003	-
XL	Dad3p	24	Spc34p	70	31	2	2	0	+/-
XL	Dad3p	31	Dad1p	1	6	1	1	0	-
XL	Dad3p	31	Dad3p	83	11	1	1	0	-
XL	Dad3p	31	Dad3p	94	11	1	1	0	-
XL	Dad3p	31	Duo1p	33	7	1	1	0.001	-
XL	Dad3p	31	Duo1p	86	6	1	1	0	-
XL	Dad3p	31	Spc34p	70	21	1	1	0	+/-

XL	Dad3p	63	Dad1p	1	4	1	1	0.001	-
XL	Dad3p	63	Dad3p	83	11	1	1	0	-
XL	Dad3p	72	Dad1p	1	11	1	1	0	-
XL	Dad3p	72	Dad1p	94	1	1	1	0.003	-
XL*	Dad3p	72	Dad3p	83	67	1	1	0	+/-
XL	Dad3p	72	Dad3p	94	18	2	2	0	-
XL	Dad3p	72	Dam1p	7	3	1	1	0.006	-
XL	Dad3p	72	Duo1p	169	6	1	1	0.005	-
XL	Dad3p	72	Duo1p	236	2	1	1	0.001	-
XL	Dad3p	83	Ask1p	6	5	2	2	0.002	+/-
XL	Dad3p	83	Ask1p	86	10	1	1	0	-
XL	Dad3p	83	Ask1p	151	8	1	1	0	+/-
XL	Dad3p	83	Ask1p	198	8	1	1	0	+/-
XL	Dad3p	83	Ask1p	225	5	1	1	0	-
XL	Dad3p	83	Dad1p	1	32	1	1	0	+/-
XL	Dad3p	83	Dad1p	11	8	2	2	0	-
XL	Dad3p	83	Dad1p	18	16	1	1	0	-
XL	Dad3p	83	Dad1p	67	3	1	1	0	-
XL	Dad3p	83	Dad1p	92	8	1	1	0.002	+/-
XL	Dad3p	83	Dad1p	94	3	1	1	0.003	+/-
XL	Dad3p	83	Dad2p	1	31	2	2	0	+/-
XL	Dad3p	83	Dad2p	11	2	1	1	0.001	-
XL	Dad3p	83	Dad2p	13	1	1	1	0.006	-
XL	Dad3p	83	Dad2p	20	1	1	1	0.003	-
XL	Dad3p	83	Dad3p	83	28	1	1	0	+/-
XL	Dad3p	83	Dad3p	94	9	1	1	0	-
XL	Dad3p	83	Dad4p	1	94	1	1	0	+/-
XL	Dad3p	83	Dad4p	21	8	1	1	0.007	-
XL	Dad3p	83	Dad4p	42	24	2	2	0	+/-
XL	Dad3p	83	Dam1p	7	4	1	1	0.004	-
XL	Dad3p	83	Dam1p	40	6	1	1	0	+
XL	Dad3p	83	Dam1p	171	5	1	1	0	-
XL	Dad3p	83	Dam1p	252	2	1	1	0.003	-
XL	Dad3p	83	Dam1p	256	13	1	1	0.001	-
XL	Dad3p	83	Dam1p	307	8	1	1	0	-
XL	Dad3p	83	Duo1p	2	11	1	1	0	-

XL	Dad3p	83	Duo1p	14	31	1	1	0	+/-
XL	Dad3p	83	Duo1p	33	13	1	1	0	+/-
XL	Dad3p	83	Duo1p	36	27	1	1	0	+/-
XL	Dad3p	83	Duo1p	122	7	1	1	0.002	-
XL	Dad3p	83	Duo1p	131	9	1	1	0	-
XL	Dad3p	83	Duo1p	159	14	1	1	0	+/-
XL	Dad3p	83	Duo1p	169	9	1	1	0.003	-
XL	Dad3p	83	Duo1p	174	2	1	1	0.003	-
XL	Dad3p	83	Duo1p	215	36	1	1	0.001	-
XL	Dad3p	83	Duo1p	236	17	1	1	0.002	-
XL	Dad3p	83	Hsk3p	5	6	1	1	0.001	-
XL	Dad3p	83	Hsk3p	28	6	1	1	0	-
XL	Dad3p	83	Hsk3p	35	4	1	1	0	-
XL	Dad3p	83	Spc19p	44	11	1	1	0	-
XL	Dad3p	83	Spc19p	59	5	1	1	0	-
XL	Dad3p	83	Spc19p	76	4	1	1	0.001	-
XL	Dad3p	83	Spc19p	104	6	1	1	0	+/-
XL	Dad3p	83	Spc34p	46	3	1	1	0.002	-
XL	Dad3p	83	Spc34p	105	2	1	1	0.003	-
XL	Dad3p	83	Spc34p	249	2	1	1	0.002	-
XL	Dad3p	94	Ask1p	1	1	1	1	0	-
XL	Dad3p	94	Ask1p	86	2	1	1	0.001	-
XL	Dad3p	94	Ask1p	151	2	1	1	0	+
XL	Dad3p	94	Ask1p	198	1	1	1	0	+
XL	Dad3p	94	Ask1p	225	4	1	1	0.001	+
XL	Dad3p	94	Ask1p	260	4	1	1	0.006	-
XL	Dad3p	94	Dad1p	1	23	2	2	0	+/-
XL	Dad3p	94	Dad1p	11	5	1	1	0.001	-
XL	Dad3p	94	Dad1p	18	6	1	1	0.001	-
XL	Dad3p	94	Dad2p	1	17	2	2	0	+/-
XL	Dad3p	94	Dad2p	11	1	1	1	0.003	-
XL	Dad3p	94	Dad2p	13	13	2	2	0.001	+/-
XL	Dad3p	94	Dad2p	20	1	1	1	0.003	-
XL	Dad3p	94	Dad3p	94	28	2	2	0	-
XL	Dad3p	94	Dad4p	1	54	2	2	0	+/-
XL	Dad3p	94	Dad4p	21	11	2	2	0	-

XL	Dad3p	94	Dad4p	42	8	1	1	0	+/-
XL	Dad3p	94	Dam1p	40	3	1	1	0	+
XL	Dad3p	94	Dam1p	171	4	1	1	0	-
XL	Dad3p	94	Dam1p	256	7	1	1	0	-
XL	Dad3p	94	Duo1p	2	2	1	1	0	-
XL	Dad3p	94	Duo1p	33	17	1	1	0.001	+/-
XL	Dad3p	94	Duo1p	36	9	1	1	0.001	+/-
XL	Dad3p	94	Duo1p	122	1	1	1	0.003	-
XL	Dad3p	94	Duo1p	131	1	1	1	0.003	-
XL	Dad3p	94	Duo1p	159	6	2	2	0	-
XL	Dad3p	94	Duo1p	169	1	1	1	0.005	-
XL	Dad3p	94	Duo1p	174	1	1	1	0.004	-
XL	Dad3p	94	Duo1p	177	7	1	1	0.003	-
XL	Dad3p	94	Duo1p	183	3	1	1	0.001	-
XL	Dad3p	94	Duo1p	215	8	1	1	0.003	-
XL	Dad3p	94	Duo1p	236	15	1	1	0.001	-
XL	Dad3p	94	Hsk3p	5	8	2	2	0	-
XL	Dad3p	94	Hsk3p	28	21	2	2	0	+/-
XL	Dad3p	94	Hsk3p	35	10	1	1	0	-
XL	Dad3p	94	Spc19p	1	1	1	1	0.001	-
XL	Dad3p	94	Spc19p	2	1	1	1	0.001	-
XL	Dad3p	94	Spc19p	44	1	1	1	0.003	-
XL	Dad3p	94	Spc19p	59	3	1	1	0.001	-
XL	Dad3p	94	Spc19p	76	1	1	1	0.005	-
XL	Dad3p	94	Spc34p	70	1	1	1	0.004	-
XL	Dad3p	94	Spc34p	105	3	1	1	0.003	-
XL	Dad3p	94	Spc34p	249	2	1	1	0.003	-
XL	Dad4p	1	Ask1p	1	10	2	2	0	-
XL	Dad4p	1	Ask1p	151	4	1	1	0	+/-
XL	Dad4p	1	Ask1p	225	8	1	1	0	+/-
XL	Dad4p	1	Dad2p	1	81	2	2	0	+/-
XL	Dad4p	1	Dad2p	11	12	1	1	0	+/-
XL	Dad4p	1	Dad2p	13	9	1	1	0	-
XL	Dad4p	1	Dad2p	20	2	1	1	0.001	-
XL	Dad4p	1	Dad4p	1	21	1	1	0	+/-
XL	Dad4p	1	Dad4p	2	4	1	1	0	+

XL	Dad4p	1	Dad4p	21	16	1	1	0.001	+/-
XL	Dad4p	1	Dam1p	5	4	1	1	0	+/-
XL	Dad4p	1	Dam1p	7	15	1	1	0.002	+/-
XL	Dad4p	1	Dam1p	40	6	1	1	0	+
XL	Dad4p	1	Dam1p	252	9	1	1	0	-
XL	Dad4p	1	Dam1p	256	5	1	1	0.001	+/-
XL	Dad4p	1	Dam1p	307	13	1	1	0.001	+/-
XL	Dad4p	1	Dam1p	330	5	1	1	0	-
XL	Dad4p	1	Duo1p	33	19	1	1	0	+/-
XL	Dad4p	1	Duo1p	36	4	1	1	0.001	+
XL	Dad4p	1	Duo1p	73	2	1	1	0.004	-
XL	Dad4p	1	Duo1p	131	2	1	1	0.003	-
XL	Dad4p	1	Duo1p	169	3	1	1	0.003	-
XL	Dad4p	1	Duo1p	177	4	1	1	0.005	-
XL	Dad4p	1	Duo1p	215	13	1	1	0.001	-
XL	Dad4p	1	Hsk3p	5	28	2	2	0	+/-
XL	Dad4p	1	Spc34p	46	1	1	1	0.002	-
XL	Dad4p	1	Spc34p	94	2	1	1	0	-
XL	Dad4p	1	Spc34p	243	3	1	1	0.002	-
XL	Dad4p	1	Spc34p	249	1	1	1	0.001	-
XL	Dad4p	2	Dam1p	1	29	1	0	0	+/-
XL	Dad4p	21	Dad2p	29	3	1	1	0.003	-
XL	Dad4p	21	Dad4p	21	3	1	1	0.005	-
XL	Dad4p	21	Dam1p	7	15	2	2	0.006	+/-
XL	Dad4p	21	Duo1p	236	3	1	1	0.007	-
XL	Dad4p	21	Hsk3p	5	3	1	1	0.009	-
XL	Dad4p	42	Hsk3p	28	28	2	2	0	+/-
XL	Dad4p	42	Hsk3p	35	1	1	1	0	-
XL	Dam1p	5	Spc34p	2	25	1	1	0.001	+/-
XL	Dam1p	7	Ask1p	86	1	1	1	0.003	-
XL	Dam1p	7	Ask1p	151	6	1	1	0.002	+
XL	Dam1p	7	Ask1p	198	3	1	1	0	+
XL	Dam1p	7	Dam1p	40	37	2	2	0	+/-
XL	Dam1p	7	Spc34p	2	15	2	2	0.01	+/-
XL	Dam1p	7	Spc34p	25	1	1	1	0.003	+
XL	Dam1p	40	Ask1p	225	3	1	1	0	+

XL	Dam1p	40	Spc34p	1	24	1	1	0	+/-
XL	Dam1p	40	Spc34p	2	51	2	2	0	+/-
XL	Dam1p	132	Ask1p	1	19	3	3	0	+/-
XL	Dam1p	132	Dam1p	171	2	1	1	0.003	+/-
XL	Dam1p	138	Dam1p	155	3	1	1	0.002	-
XL	Dam1p	138	Dam1p	156	1	1	1	0.004	-
XL	Dam1p	138	Dam1p	171	16	1	1	0	+/-
XL	Dam1p	138	Dam1p	256	14	1	1	0	+/-
XL	Dam1p	138	Dam1p	307	4	1	1	0.001	+/-
XL	Dam1p	155	Dam1p	171	27	1	1	0	+/-
XL	Dam1p	155	Dam1p	241	8	1	1	0.003	-
XL	Dam1p	155	Dam1p	256	10	1	1	0.003	+/-
XL	Dam1p	155	Spc34p	249	1	1	1	0.003	+
XL	Dam1p	156	Dam1p	171	45	2	2	0	+/-
XL	Dam1p	156	Dam1p	241	9	1	1	0.005	-
XL	Dam1p	156	Dam1p	252	1	1	1	0.006	-
XL	Dam1p	156	Dam1p	256	7	2	2	0.003	+/-
XL	Dam1p	156	Dam1p	307	4	2	2	0.003	+/-
XL	Dam1p	166	Dam1p	241	7	1	1	0.002	-
XL	Dam1p	166	Dam1p	252	5	1	1	0	+/-
XL	Dam1p	166	Dam1p	256	9	1	1	0	+/-
XL	Dam1p	166	Dam1p	307	12	1	1	0.001	+/-
XL	Dam1p	166	Dam1p	320	3	1	1	0.007	-
XL	Dam1p	166	Dam1p	330	3	1	1	0	-
XL	Dam1p	171	Ask1p	1	12	2	2	0	+/-
XL	Dam1p	171	Ask1p	23	1	1	1	0.001	-
XL	Dam1p	171	Ask1p	151	1	1	1	0	-
XL	Dam1p	171	Ask1p	225	5	1	1	0	+
XL	Dam1p	171	Dad2p	13	13	1	1	0.001	+/-
XL	Dam1p	171	Dad2p	20	7	1	1	0	+
XL	Dam1p	171	Dam1p	171	9	1	1	0	+
XL	Dam1p	171	Dam1p	196	2	1	1	0	+
XL	Dam1p	171	Dam1p	199	13	2	2	0	+/-
XL	Dam1p	171	Dam1p	233	5	1	1	0	+
XL	Dam1p	171	Dam1p	241	4	1	1	0	+
XL	Dam1p	171	Dam1p	252	4	1	1	0.009	-

XL	Dam1p	171	Dam1p	256	35	1	1	0	+/-
XL	Dam1p	171	Dam1p	307	21	1	1	0	+/-
XL	Dam1p	171	Dam1p	320	7	1	1	0.005	+
XL	Dam1p	171	Dam1p	330	10	1	1	0.002	+/-
XL	Dam1p	171	Hsk3p	28	1	1	1	0.007	-
XL	Dam1p	171	Spc34p	249	9	1	1	0	+
XL	Dam1p	196	Dam1p	241	1	1	1	0.006	+
XL	Dam1p	199	Dam1p	256	4	1	1	0.007	+
XL	Dam1p	233	Dam1p	256	4	1	1	0	+
XL	Dam1p	241	Ask1p	1	8	1	1	0.003	-
XL	Dam1p	241	Dam1p	252	35	1	1	0	+/-
XL	Dam1p	241	Dam1p	256	46	1	1	0	+/-
XL	Dam1p	241	Dam1p	307	24	1	1	0	+/-
XL	Dam1p	241	Dam1p	330	10	1	1	0.005	+/-
XL	Dam1p	241	Spc34p	46	10	1	1	0.005	-
XL	Dam1p	241	Spc34p	243	8	1	1	0.005	+/-
XL	Dam1p	252	Ask1p	1	9	1	1	0.003	-
XL	Dam1p	252	Dad2p	1	1	1	1	0.003	-
XL	Dam1p	252	Dam1p	256	80	2	2	0	+/-
XL	Dam1p	252	Dam1p	307	24	1	1	0.001	+/-
XL	Dam1p	252	Dam1p	320	11	1	1	0.005	-
XL	Dam1p	252	Dam1p	330	17	1	1	0.001	+/-
XL	Dam1p	252	Spc34p	2	2	1	1	0.003	-
XL	Dam1p	252	Spc34p	46	4	1	1	0.005	-
XL	Dam1p	252	Spc34p	70	5	1	1	0.003	-
XL	Dam1p	256	Ask1p	1	5	1	1	0.001	+/-
XL	Dam1p	256	Ask1p	225	3	1	1	0	+/-
XL	Dam1p	256	Dad2p	13	4	1	1	0.003	-
XL	Dam1p	256	Dam1p	307	82	2	2	0	+/-
XL	Dam1p	256	Dam1p	320	10	1	1	0.002	+/-
XL	Dam1p	256	Dam1p	330	4	1	1	0.001	-
XL	Dam1p	256	Hsk3p	28	6	1	1	0	+/-
XL	Dam1p	256	Spc34p	46	2	1	1	0.001	-
XL	Dam1p	256	Spc34p	65	1	1	1	0.003	+
XL	Dam1p	256	Spc34p	70	3	1	1	0.004	+
XL	Dam1p	256	Spc34p	126	2	1	1	0.004	-

XL	Dam1p	256	Spc34p	169	11	1	1	0	+/-
XL	Dam1p	256	Spc34p	243	20	1	1	0	+/-
XL	Dam1p	256	Spc34p	249	12	1	1	0	+/-
XL	Dam1p	307	Ask1p	1	6	1	1	0.002	-
XL	Dam1p	307	Dam1p	320	85	1	1	0.001	+/-
XL	Dam1p	307	Dam1p	330	22	1	1	0.002	+/-
XL	Dam1p	307	Spc34p	65	9	1	1	0.001	+/-
XL	Dam1p	307	Spc34p	70	6	1	1	0.004	-
XL	Dam1p	307	Spc34p	169	7	1	1	0.001	+/-
XL	Dam1p	307	Spc34p	243	9	1	1	0.009	-
XL	Dam1p	307	Spc34p	249	15	1	1	0	+/-
XL	Dam1p	320	Dam1p	330	36	1	1	0.004	+/-
XL	Dam1p	320	Spc34p	243	3	1	1	0.004	-
XL	Dam1p	320	Spc34p	249	4	1	1	0.003	-
XL	Dam1p	330	Spc34p	2	4	1	1	0.002	+/-
XL	Dam1p	330	Spc34p	243	17	1	1	0	+/-
XL	Dam1p	330	Spc34p	249	10	1	1	0.002	-
XL	Dam1p	331	Dam1p	331	2	1	1	0.009	-
XL	Dam1p	334	Ask1p	198	1	1	1	0.002	+
XL	Duo1p	2	Duo1p	2	3	1	1	0.003	-
XL	Duo1p	14	Duo1p	36	2	1	1	0.003	-
XL	Duo1p	33	Dam1p	40	45	2	2	0	+/-
XL	Duo1p	33	Dam1p	66	6	1	1	0.001	-
XL	Duo1p	33	Duo1p	66	21	2	2	0.001	-
XL	Duo1p	33	Duo1p	73	6	1	1	0.003	-
XL	Duo1p	33	Spc34p	1	1	1	1	0.005	+
XL	Duo1p	33	Spc34p	2	17	1	1	0	+/-
XL	Duo1p	33	Spc34p	249	5	1	1	0	+
XL	Duo1p	36	Dam1p	7	6	1	1	0.001	-
XL	Duo1p	36	Dam1p	40	47	2	2	0	+/-
XL	Duo1p	36	Dam1p	66	15	1	1	0	+/-
XL	Duo1p	36	Duo1p	66	26	2	2	0	+/-
XL	Duo1p	36	Spc34p	1	8	1	1	0	-
XL	Duo1p	36	Spc34p	2	39	1	1	0	+/-
XL	Duo1p	36	Spc34p	70	1	1	1	0.008	-
XL	Duo1p	36	Spc34p	249	4	1	1	0.006	+

XL	Duo1p	66	Ask1p	151	1	1	1	0	+
XL	Duo1p	66	Dam1p	7	4	1	1	0.006	-
XL	Duo1p	66	Dam1p	40	5	1	1	0	+/-
XL	Duo1p	66	Hsk3p	5	7	1	1	0.001	-
XL	Duo1p	66	Spc34p	1	38	2	2	0	+/-
XL	Duo1p	66	Spc34p	2	78	2	2	0	+/-
XL	Duo1p	66	Spc34p	249	1	1	1	0.001	+
XL	Duo1p	73	Spc34p	1	4	1	1	0.001	-
XL	Duo1p	73	Spc34p	2	12	1	1	0.002	+/-
XL	Duo1p	79	Dad2p	88	3	2	2	0.001	-
XL	Duo1p	79	Spc34p	169	1	1	1	0.004	-
XL	Duo1p	86	Dad2p	88	6	1	1	0	-
XL	Duo1p	86	Dad2p	106	1	1	1	0.002	-
XL	Duo1p	86	Duo1p	131	1	1	1	0.006	-
XL	Duo1p	122	Ask1p	1	5	1	1	0	+/-
XL	Duo1p	122	Ask1p	6	1	1	1	0.001	-
XL	Duo1p	122	Dam1p	171	4	1	1	0.001	-
XL	Duo1p	122	Duo1p	159	2	1	1	0.003	-
XL	Duo1p	122	Duo1p	215	8	1	1	0.004	-
XL	Duo1p	131	Dam1p	132	11	1	1	0.001	+/-
XL	Duo1p	131	Dam1p	138	15	1	1	0	+/-
XL	Duo1p	131	Duo1p	131	1	1	1	0.001	-
XL*	Duo1p	131	Duo1p	159	91	1	1	0	+/-
XL	Duo1p	131	Duo1p	161	6	1	1	0.003	-
XL	Duo1p	131	Duo1p	236	1	1	1	0.001	-
XL	Duo1p	131	Spc34p	249	4	1	1	0.001	-
XL	Duo1p	159	Dad2p	13	1	1	1	0	-
XL	Duo1p	159	Dam1p	132	44	1	1	0	+/-
XL	Duo1p	159	Dam1p	138	50	1	1	0	+/-
XL	Duo1p	159	Dam1p	156	1	1	1	0.005	-
XL	Duo1p	159	Dam1p	166	2	1	1	0.002	-
XL	Duo1p	159	Dam1p	171	10	1	1	0	+/-
XL	Duo1p	159	Dam1p	241	7	1	1	0	-
XL	Duo1p	159	Dam1p	252	12	1	1	0	+/-
XL	Duo1p	159	Dam1p	256	20	1	1	0	+/-
XL	Duo1p	159	Dam1p	307	10	1	1	0.003	+/-

XL*	Duo1p	159	Duo1p	161	12	1	1	0	+/-
XL	Duo1p	159	Duo1p	169	22	1	1	0.001	+/-
XL	Duo1p	159	Duo1p	215	12	1	1	0.001	-
XL	Duo1p	159	Duo1p	236	19	1	1	0	+/-
XL	Duo1p	159	Spc34p	46	15	1	1	0	+/-
XL	Duo1p	159	Spc34p	249	1	1	1	0.004	+
XL	Duo1p	160	Dam1p	256	2	1	1	0.006	+/-
XL	Duo1p	160	Duo1p	177	3	1	1	0.002	+/-
XL	Duo1p	160	Spc34p	249	2	1	1	0.002	+
XL	Duo1p	161	Dam1p	171	4	1	1	0.003	-
XL	Duo1p	161	Dam1p	252	17	2	2	0.002	+/-
XL	Duo1p	161	Dam1p	256	11	2	2	0	+/-
XL	Duo1p	161	Dam1p	307	11	2	2	0.002	+/-
XL	Duo1p	161	Duo1p	169	13	2	2	0.004	+/-
XL	Duo1p	161	Duo1p	174	1	1	1	0.009	-
XL	Duo1p	161	Duo1p	183	23	1	1	0.002	+/-
XL	Duo1p	161	Duo1p	215	13	2	2	0.007	-
XL	Duo1p	161	Duo1p	236	20	2	2	0.006	+/-
XL	Duo1p	169	Dad2p	1	5	1	1	0.002	-
XL	Duo1p	169	Dam1p	138	78	2	2	0	+/-
XL	Duo1p	169	Dam1p	155	28	1	1	0.006	+/-
XL	Duo1p	169	Dam1p	156	37	2	2	0.004	+/-
XL	Duo1p	169	Dam1p	166	47	3	3	0	+/-
XL	Duo1p	169	Dam1p	171	38	1	1	0	+/-
XL	Duo1p	169	Dam1p	252	7	1	1	0.01	+/-
XL	Duo1p	169	Dam1p	256	5	1	1	0.003	-
XL	Duo1p	169	Dam1p	307	8	1	1	0.009	+/-
XL	Duo1p	169	Duo1p	174	20	1	1	0.001	+/-
XL	Duo1p	169	Duo1p	177	60	1	1	0.001	+/-
XL	Duo1p	169	Duo1p	183	97	1	1	0	+/-
XL	Duo1p	169	Duo1p	215	21	1	1	0.006	-
XL	Duo1p	169	Duo1p	236	29	1	1	0.005	-
XL	Duo1p	169	Spc34p	243	5	1	1	0.006	+
XL	Duo1p	169	Spc34p	249	7	1	1	0.002	+
XL	Duo1p	174	Dam1p	155	35	2	2	0	+/-
XL	Duo1p	174	Dam1p	156	17	3	3	0	+/-

XL	Duo1p	174	Dam1p	171	10	1	1	0	+/-
XL	Duo1p	174	Dam1p	233	1	1	1	0.002	+
XL	Duo1p	174	Dam1p	256	4	1	1	0.002	+/-
XL	Duo1p	174	Dam1p	307	7	1	1	0.006	+
XL	Duo1p	174	Duo1p	215	24	1	1	0	+/-
XL	Duo1p	174	Duo1p	236	3	1	1	0.007	+/-
XL	Duo1p	177	Ask1p	225	1	1	1	0.004	+
XL	Duo1p	177	Dam1p	155	50	3	3	0	+/-
XL	Duo1p	177	Dam1p	156	40	4	4	0	+/-
XL	Duo1p	177	Dam1p	166	9	1	1	0	+/-
XL	Duo1p	177	Dam1p	171	34	2	2	0	+/-
XL	Duo1p	177	Dam1p	241	5	1	1	0.002	+/-
XL	Duo1p	177	Dam1p	252	5	1	1	0.005	+/-
XL	Duo1p	177	Dam1p	256	7	1	1	0.002	+/-
XL	Duo1p	177	Dam1p	307	4	1	1	0.005	+
XL	Duo1p	177	Duo1p	177	1	1	1	0.006	+
XL	Duo1p	177	Duo1p	215	29	1	1	0	+/-
XL	Duo1p	177	Duo1p	236	13	1	1	0	+/-
XL	Duo1p	177	Spc34p	243	1	1	1	0.001	+
XL	Duo1p	177	Spc34p	249	1	1	1	0.002	+
XL	Duo1p	183	Dam1p	138	20	1	1	0.001	+/-
XL	Duo1p	183	Dam1p	155	79	1	1	0.001	+/-
XL	Duo1p	183	Dam1p	156	13	2	2	0.002	+/-
XL	Duo1p	183	Dam1p	166	23	1	1	0.001	+/-
XL	Duo1p	183	Dam1p	171	59	1	1	0	+/-
XL	Duo1p	183	Dam1p	256	31	1	1	0	+/-
XL	Duo1p	183	Duo1p	215	103	1	1	0	+/-
XL	Duo1p	183	Duo1p	222	15	1	1	0.004	+/-
XL	Duo1p	183	Duo1p	236	75	1	1	0.002	+/-
XL	Duo1p	183	Spc34p	249	1	1	1	0.004	+
XL	Duo1p	215	Ask1p	1	6	1	1	0.005	-
XL	Duo1p	215	Dam1p	155	24	1	1	0.005	-
XL	Duo1p	215	Dam1p	156	19	1	1	0.003	-
XL	Duo1p	215	Dam1p	166	26	1	1	0	-
XL	Duo1p	215	Dam1p	171	38	1	1	0	+/-
XL	Duo1p	215	Dam1p	196	2	1	1	0.006	+

XL	Duo1p	215	Dam1p	199	1	1	1	0.009	+
XL	Duo1p	215	Dam1p	233	4	1	1	0.004	+
XL	Duo1p	215	Dam1p	241	4	1	1	0.006	-
XL	Duo1p	215	Dam1p	252	10	1	1	0.003	-
XL	Duo1p	215	Dam1p	307	12	1	1	0.002	+/-
XL	Duo1p	215	Duo1p	236	103	1	1	0.002	+/-
XL	Duo1p	215	Hsk3p	28	9	1	1	0.002	-
XL	Duo1p	215	Hsk3p	67	4	1	1	0.004	-
XL	Duo1p	215	Spc34p	46	7	1	1	0.003	-
XL	Duo1p	215	Spc34p	243	2	1	1	0.006	+
XL	Duo1p	222	Duo1p	236	17	2	2	0.01	+/-
XL	Duo1p	236	Ask1p	198	1	1	1	0.001	-
XL	Duo1p	236	Ask1p	225	8	1	1	0.003	-
XL	Duo1p	236	Dad2p	1	1	1	1	0.002	-
XL	Duo1p	236	Dad2p	11	1	1	1	0.006	-
XL	Duo1p	236	Dad2p	20	5	1	1	0.005	-
XL	Duo1p	236	Dad2p	29	11	1	1	0.002	-
XL	Duo1p	236	Dam1p	138	7	1	1	0.001	-
XL	Duo1p	236	Dam1p	156	8	2	2	0.003	-
XL	Duo1p	236	Dam1p	166	10	1	1	0.003	-
XL	Duo1p	236	Dam1p	171	31	1	1	0	+/-
XL	Duo1p	236	Dam1p	233	2	1	1	0	+
XL	Duo1p	236	Dam1p	252	3	1	1	0.001	-
XL	Duo1p	236	Dam1p	256	22	1	1	0.002	+/-
XL	Duo1p	236	Dam1p	307	4	1	1	0.002	+
XL	Duo1p	236	Dam1p	335	1	1	1	0.002	+
XL	Duo1p	236	Duo1p	236	3	2	2	0.002	+
XL	Duo1p	236	Hsk3p	28	5	1	1	0	-
XL	Duo1p	236	Spc34p	65	8	2	2	0.001	-
XL	Duo1p	236	Spc34p	70	24	1	1	0.001	-
XL	Duo1p	236	Spc34p	169	9	1	1	0.001	-
XL	Duo1p	236	Spc34p	243	5	1	1	0.003	-
XL	Hsk3p	5	Dad2p	1	5	1	1	0.001	-
XL	Hsk3p	5	Dad2p	13	6	1	1	0.002	-
XL	Hsk3p	5	Hsk3p	5	24	1	1	0	-
XL	Hsk3p	5	Hsk3p	35	5	1	1	0.005	-

XL	Hsk3p	35	Ask1p	1	1	1	1	0.003	-
XL	Hsk3p	67	Spc34p	46	4	1	1	0	-
XL	Spc19p	1	Ask1p	225	3	1	1	0	+
XL	Spc19p	1	Dad2p	1	2	1	1	0	-
XL	Spc19p	1	Dad2p	13	59	4	4	0	+/-
XL	Spc19p	1	Dad4p	1	1	1	1	0.001	-
XL	Spc19p	1	Dam1p	5	2	1	1	0.001	+
XL	Spc19p	1	Dam1p	7	6	1	1	0	+/-
XL	Spc19p	1	Dam1p	307	3	1	1	0.001	-
XL	Spc19p	1	Dam1p	330	1	1	1	0.005	-
XL	Spc19p	1	Duo1p	236	2	1	1	0.002	-
XL	Spc19p	1	Hsk3p	1	3	1	1	0	+/-
XL	Spc19p	1	Hsk3p	2	5	1	1	0.003	+/-
XL	Spc19p	1	Hsk3p	5	9	1	1	0	-
XL	Spc19p	2	Dad2p	13	61	3	3	0	+/-
XL	Spc19p	2	Dam1p	1	3	1	1	0	+
XL	Spc19p	2	Dam1p	7	8	1	1	0	+/-
XL	Spc19p	2	Dam1p	252	1	1	1	0	-
XL	Spc19p	2	Dam1p	330	1	1	1	0.001	-
XL	Spc19p	2	Duo1p	236	2	1	1	0.001	-
XL	Spc19p	2	Hsk3p	5	11	1	1	0.001	-
XL	Spc19p	20	Dad2p	20	2	1	1	0	+/-
XL	Spc19p	20	Dam1p	171	1	1	1	0.001	-
XL	Spc19p	20	Dam1p	241	1	1	1	0.005	-
XL	Spc19p	20	Duo1p	215	11	2	2	0	+/-
XL	Spc19p	44	Dad2p	20	3	1	1	0.003	-
XL	Spc19p	44	Dam1p	241	1	1	1	0.005	+
XL	Spc19p	44	Dam1p	256	9	1	1	0	+
XL	Spc19p	44	Duo1p	131	3	1	1	0.001	-
XL	Spc19p	44	Duo1p	215	16	1	1	0	+/-
XL	Spc19p	44	Duo1p	236	12	1	1	0	+/-
XL	Spc19p	44	Spc19p	59	21	1	1	0	+/-
XL	Spc19p	44	Spc19p	104	1	1	1	0.002	-
XL	Spc19p	44	Spc34p	79	4	1	1	0.001	-
XL	Spc19p	44	Spc34p	112	1	1	1	0.007	-
XL	Spc19p	44	Spc34p	169	3	1	1	0.002	-

XL	Spc19p	59	Dad4p	1	4	1	1	0	-
XL	Spc19p	59	Dam1p	256	10	1	1	0.002	+/-
XL	Spc19p	59	Dam1p	307	4	1	1	0.003	+/-
XL	Spc19p	59	Spc19p	76	5	1	1	0	-
XL	Spc19p	59	Spc19p	104	4	1	1	0	-
XL	Spc19p	59	Spc19p	146	1	1	1	0.002	+
XL	Spc19p	59	Spc34p	46	8	1	1	0.002	-
XL	Spc19p	59	Spc34p	53	35	1	1	0	+/-
XL	Spc19p	59	Spc34p	70	6	1	1	0.001	+/-
XL	Spc19p	59	Spc34p	79	2	1	1	0.007	-
XL	Spc19p	59	Spc34p	118	11	1	1	0	+/-
XL	Spc19p	59	Spc34p	126	17	1	1	0	+/-
XL	Spc19p	59	Spc34p	169	18	1	1	0	-
XL	Spc19p	59	Spc34p	249	4	1	1	0.001	-
XL	Spc19p	76	Dad4p	1	2	1	1	0.004	-
XL	Spc19p	76	Spc34p	169	5	1	1	0.006	+
XL	Spc19p	76	Spc34p	198	69	2	2	0	+/-
XL	Spc19p	104	Ask1p	1	3	1	1	0.001	+
XL	Spc19p	104	Ask1p	151	3	1	1	0	+
XL	Spc19p	104	Ask1p	198	2	1	1	0.001	+
XL	Spc19p	104	Ask1p	225	11	1	1	0	+
XL	Spc19p	104	Dad2p	13	3	1	1	0	+
XL	Spc19p	104	Dad2p	20	4	1	1	0	+
XL	Spc19p	104	Dad4p	1	4	1	1	0	+
XL	Spc19p	104	Dam1p	7	4	1	1	0	+
XL	Spc19p	104	Dam1p	171	5	1	1	0	+
XL	Spc19p	104	Dam1p	256	10	1	1	0	+/-
XL	Spc19p	104	Dam1p	307	3	1	1	0.002	-
XL	Spc19p	104	Duo1p	33	3	2	2	0.002	+
XL	Spc19p	104	Duo1p	36	6	1	1	0	+
XL	Spc19p	104	Duo1p	122	1	1	1	0.002	+
XL	Spc19p	104	Duo1p	169	5	1	1	0	+
XL	Spc19p	104	Duo1p	177	7	1	1	0	+
XL	Spc19p	104	Duo1p	183	3	1	1	0	+
XL	Spc19p	104	Spc19p	146	52	1	1	0	+/-
XL	Spc19p	104	Spc19p	152	5	1	1	0.003	+/-

XL	Spc19p	104	Spc19p	153	25	2	2	0	+/-
XL	Spc19p	104	Spc19p	158	12	1	1	0.002	+/-
XL	Spc19p	104	Spc19p	163	4	1	1	0.004	-
XL	Spc19p	104	Spc34p	2	6	1	1	0	+
XL	Spc19p	104	Spc34p	46	1	1	1	0.001	-
XL	Spc19p	104	Spc34p	112	1	1	1	0.002	+
XL	Spc19p	104	Spc34p	198	4	1	1	0	+
XL	Spc19p	104	Spc34p	243	8	1	1	0	-
XL	Spc19p	104	Spc34p	249	14	1	1	0	+/-
XL	Spc19p	104	Spc34p	259	20	1	1	0	+/-
XL	Spc19p	104	Spc34p	274	75	2	2	0	+/-
XL	Spc19p	104	Spc34p	278	49	2	2	0	+/-
XL	Spc19p	104	Spc34p	280	26	2	2	0	+/-
XL	Spc19p	146	Spc19p	152	2	1	1	0.01	+
XL	Spc19p	146	Spc19p	153	42	2	2	0	+/-
XL	Spc19p	146	Spc34p	249	15	1	1	0	+/-
XL	Spc19p	149	Spc19p	153	10	2	2	0.007	+/-
XL	Spc19p	151	Spc19p	158	64	4	4	0	+/-
XL*	Spc19p	152	Spc19p	158	4	1	1	0.005	-
XL	Spc19p	152	Spc34p	249	1	1	1	0.01	-
XL	Spc19p	153	Spc34p	243	1	1	1	0.007	-
XL	Spc19p	153	Spc34p	249	3	1	1	0.006	-
XL	Spc19p	158	Dad4p	1	2	1	1	0	+
XL	Spc19p	158	Duo1p	183	4	1	1	0.005	+
XL	Spc19p	158	Duo1p	236	4	1	1	0.005	+
XL	Spc19p	158	Spc34p	2	1	1	1	0.003	+
XL	Spc19p	158	Spc34p	65	2	1	1	0.003	+
XL	Spc19p	158	Spc34p	198	25	1	1	0	+/-
XL	Spc19p	158	Spc34p	249	5	1	1	0.004	+
XL	Spc19p	163	Spc19p	163	1	1	1	0.004	-
XL	Spc19p	163	Spc34p	249	2	1	1	0.005	+
XL	Spc34p	2	Spc34p	249	9	1	1	0.001	+
XL	Spc34p	2	Spc34p	274	2	1	1	0.002	+
XL	Spc34p	25	Spc34p	46	3	1	1	0.005	-
XL*	Spc34p	41	Spc34p	46	51	1	1	0	+/-
XL	Spc34p	46	Spc34p	53	39	1	1	0.001	+/-

XL	Spc34p	46	Spc34p	65	1	1	1	0.005	+
XL	Spc34p	46	Spc34p	70	5	1	1	0.007	+/-
XL	Spc34p	46	Spc34p	88	4	1	1	0.003	-
XL	Spc34p	46	Spc34p	105	3	1	1	0.006	-
XL	Spc34p	53	Spc34p	70	6	1	1	0.003	+/-
XL	Spc34p	53	Spc34p	79	1	1	1	0.003	-
XL	Spc34p	53	Spc34p	126	6	1	1	0.003	+/-
XL	Spc34p	65	Spc34p	79	1	1	1	0.002	-
XL	Spc34p	65	Spc34p	88	23	1	1	0	+/-
XL	Spc34p	65	Spc34p	105	2	1	1	0.005	+
XL	Spc34p	65	Spc34p	126	7	1	1	0	-
XL	Spc34p	65	Spc34p	169	25	2	2	0	+/-
XL	Spc34p	70	Spc34p	70	2	1	1	0.001	-
XL	Spc34p	70	Spc34p	79	9	2	2	0.002	+/-
XL	Spc34p	70	Spc34p	88	26	2	2	0	+/-
XL	Spc34p	70	Spc34p	105	11	1	1	0.002	+/-
XL	Spc34p	70	Spc34p	169	8	1	1	0.002	+/-
XL	Spc34p	79	Spc34p	118	3	1	1	0.004	-
XL	Spc34p	79	Spc34p	126	3	1	1	0.003	-
XL	Spc34p	79	Spc34p	169	7	1	1	0.001	-
XL	Spc34p	88	Spc34p	105	1	1	1	0.005	-
XL	Spc34p	88	Spc34p	118	15	1	1	0.001	+/-
XL	Spc34p	94	Spc34p	105	14	1	1	0.003	+/-
XL	Spc34p	105	Spc34p	118	13	1	1	0.003	+/-
XL	Spc34p	105	Spc34p	169	8	1	1	0.004	+/-
XL	Spc34p	112	Spc34p	126	63	4	4	0	+/-
XL	Spc34p	112	Spc34p	243	1	1	1	0.002	-
XL*	Spc34p	118	Spc34p	126	30	1	1	0.004	+/-
XL	Spc34p	169	Spc34p	198	8	1	1	0.001	-
XL	Spc34p	169	Spc34p	249	1	1	1	0.001	-
XL	Spc34p	198	Spc34p	249	10	1	1	0.001	-
XL	Spc34p	243	Spc34p	249	31	1	1	0	+/-
XL	Spc34p	243	Spc34p	274	1	1	1	0.006	+
XL	Spc34p	249	Spc34p	259	29	1	1	0	+/-
XL	Spc34p	249	Spc34p	274	27	1	1	0	+/-
XL	Spc34p	249	Spc34p	278	5	1	1	0.001	-

XL	Spc34p	259	Spc34p	278	3	1	1	0.009	-
XL	Spc34p	274	Spc34p	278	133	2	2	0	+/-
XL	Spc34p	274	Spc34p	280	14	2	2	0.004	+/-
LL	Ask1p	1	Ask1p	6	32	2	2	0	+/-
LL*	Ask1p	1	Ask1p	12	3	1	1	0.006	-
LL	Ask1p	6	Ask1p	12	8	1	1	0	-
LL	Ask1p	12	Ask1p	23	26	2	2	0	-
LL	Ask1p	196	Ask1p	198	14	1	1	0	+/-
LL*	Ask1p	222	Ask1p	225	1	1	1	0.001	+
LL	Ask1p	225	Ask1p	253	80	2	2	0.002	+/-
LL	Ask1p	253	Ask1p	260	472	4	4	0	+/-
LL	Ask1p	253	Ask1p	277	2	2	2	0.003	+/-
LL	Ask1p	260	Ask1p	277	72	2	2	0	+/-
LL*	Ask1p	260	Ask1p	281	1	1	1	0.01	+
LL	Ask1p	277	Ask1p	281	198	2	2	0	+/-
LL*	Dad1p	1	Dad1p	11	90	2	2	0	+/-
LL	Dad1p	11	Dad1p	18	101	1	1	0	+/-
LL	Dad1p	92	Dad1p	94	100	1	1	0	+/-
LL	Dad2p	13	Dad2p	20	36	2	2	0	+/-
LL	Dad2p	87	Dad2p	88	12	1	1	0	+/-
LL	Dad2p	88	Dad2p	106	13	1	1	0	-
LL*	Dad3p	1	Dad3p	15	12	1	1	0.001	+/-
LL*	Dad3p	1	Dad3p	17	4	2	2	0.004	-
LL*	Dad3p	15	Dad3p	17	63	3	3	0	+/-
LL	Dad3p	15	Dad3p	24	1	1	1	0.004	-
LL	Dad3p	17	Dad3p	24	29	4	4	0	+/-
LL	Dad3p	24	Dad3p	31	24	1	1	0	+/-
LL*	Dad3p	72	Dad3p	83	30	1	1	0	-
LL	Dam1p	2	Dam1p	5	6	2	2	0.004	+/-
LL	Dam1p	2	Dam1p	7	4	1	1	0.002	+/-
LL	Dam1p	5	Dam1p	7	8	1	1	0	+/-
LL	Dam1p	132	Dam1p	138	22	1	1	0	+/-
LL	Dam1p	153	Dam1p	155	5	1	1	0.001	-
LL	Dam1p	155	Dam1p	156	30	4	4	0	+/-
LL	Dam1p	155	Dam1p	166	1	1	1	0.005	-
LL	Dam1p	156	Dam1p	166	1	1	1	0.005	-

LL	Dam1p	166	Dam1p	171	112	4	4	0	+/-
LL	Dam1p	196	Dam1p	199	106	2	2	0	+/-
LL	Dam1p	233	Dam1p	241	46	1	1	0	+/-
LL	Dam1p	330	Dam1p	331	5	1	1	0	+/-
LL	Dam1p	331	Dam1p	334	3	2	2	0	+/-
LL	Dam1p	334	Dam1p	335	55	2	2	0	+/-
LL	Duo1p	2	Duo1p	14	10	1	1	0.002	-
LL	Duo1p	33	Duo1p	36	212	1	1	0	+/-
LL	Duo1p	66	Duo1p	73	22	1	1	0	+/-
LL	Duo1p	73	Duo1p	79	9	1	1	0.001	+/-
LL	Duo1p	79	Duo1p	86	46	2	2	0	-
LL*	Duo1p	131	Duo1p	159	1	1	1	0.01	-
LL	Duo1p	159	Duo1p	160	14	1	1	0	+/-
LL*	Duo1p	159	Duo1p	161	7	1	1	0	-
LL	Duo1p	160	Duo1p	161	8	1	1	0	+/-
LL	Duo1p	174	Duo1p	177	196	1	1	0	+/-
LL	Duo1p	177	Duo1p	183	47	1	1	0	+/-
LL	Hsk3p	1	Hsk3p	5	2	1	1	0.009	+
LL	Hsk3p	28	Hsk3p	35	37	1	1	0	+/-
LL	Spc19p	76	Spc19p	79	29	1	1	0.001	+/-
LL	Spc19p	103	Spc19p	104	69	3	3	0	+/-
LL	Spc19p	146	Spc19p	147	37	3	3	0	+/-
LL	Spc19p	146	Spc19p	149	95	2	2	0	+/-
LL	Spc19p	146	Spc19p	151	2	1	1	0.004	-
LL	Spc19p	147	Spc19p	149	20	1	1	0	+/-
LL	Spc19p	152	Spc19p	153	7	3	3	0.001	-
LL*	Spc19p	152	Spc19p	158	1	1	1	0	+
LL	Spc19p	158	Spc19p	163	3	2	2	0.003	-
LL	Spc19p	158	Spc19p	165	1	1	1	0.005	+
LL	Spc19p	163	Spc19p	165	3	1	1	0.002	+/-
LL	Spc34p	25	Spc34p	41	22	1	1	0	+/-
LL*	Spc34p	41	Spc34p	46	1	1	1	0	+
LL	Spc34p	53	Spc34p	65	9	1	1	0.002	+/-
LL	Spc34p	65	Spc34p	70	96	2	2	0	+/-
LL	Spc34p	105	Spc34p	112	16	1	1	0	+/-
LL	Spc34p	112	Spc34p	118	118	5	5	0	+/-

LL	Spc34p	118	Spc34p	126	2	1	1	0.006	-
LL	Spc34p	162	Spc34p	169	2	1	1	0	-
LL	Spc34p	243	Spc34p	248	6	1	1	0.001	+/-
LL	Spc34p	248	Spc34p	249	50	3	3	0	+/-
LL	Spc34p	278	Spc34p	280	5	2	2	0.005	-
**XL=cross-link; LL=loop-link; *Identified as both a XL and a LL**									

Supplementary Table 7: Relationships within the Dam1 complex that do not change in the presence versus the absence of microtubules.

Interaction Pair	Multiple cross-links	Few/no cross-links
Ask1p/Dam1p	X	
Dad1p/Dad3p	X	
Dad1p/Duo1p	X	
Dad1p/Spc34p	X	
Dad3p/Spc34p	X	
Dad3p/Dad4p	X	
Dad4p/Spc34p	X	
Dam1p/Duo1p	X	
Dam1p/Spc19p	X	
Dam1p/Spc34p	X	
Duo1p/Spc34p	X	
Spc19p/Spc34p	X	
Ask1p/Dad2p		X
Ask1p/Dad4p		X
Ask1p/Hsk3p		X
Dad1p/Hsk3p		X
Dad2p/Hsk3p		X
Dad4p/Spc34p		X
Hsk3p/Spc34p		X

Supplementary Table 8: Relationships within the Dam1 complex that change in the presence versus the absence of microtubules.

Interaction Pair	Multiple cross-links	Few/no cross-links	Multiple cross-links	Few/no cross-links
	In absence of microtubules		In presence of microtubules	
Ask1p/Spc19p		X	X	
Ask1p/Spc34p		X	X	
Dad2p/Duo1p	X			X
Dad3p/Duo1p	X			X
Dad2p-C/N-Spc34p	X			X
Dad3p-C/Spc34p	X			X
N-Duo1p/Spc19p		X	X	
Duo1p-C/N-Spc19p		X	X	

Supplementary Table 9: Dam1 complex to tubulin cross-links found at 1% FDR.

Peptide 1 (position)	Peptide 2 (position)	Protein 1 (position)	Protein 2 (position)	Peptide Q-Value	Num PSMs
WTKPTASSSR(3)	TLKLTTPTYGDLNHLVSATMSGVTTCLR(3)	Duo1p (236)	β-tubulin* (216)	0	7
WTKPTASSSR(3)	GDVVPKDVNAAIATIK(6)	Duo1p (236)	α-tubulin (326)	0	14
WTKPTASSSR(3)	MSMKEVDEQMLNVQNK(4)	Duo1p (236)	β-tubulin* (324)	0	7
LNNNTNSKLR(8)	FDLMYAKR(7)	Dam1p(252)	α-tubulin (401)	0.004	4
KTHVPASK(1)	VGINYQPPTVVPGGDLAKVQR (18)	Duo1p (215)	α-tubulin (370)	0.001	5
KTHVPASK(1)	FDLMYAKR(7)	Duo1p (215)	α-tubulin (401)	0.001	25
HSVAKK(5)	FDLMYAKR(7)	Dam1p (330)	α-tubulin (401)	0.006	4
KSILHTIR(1)	FDLMYAKR(7)	Dam1p (256)	α-tubulin (401)	0	10
WTKPTASSSR(3)	FDLMYAKR(7)	Duo1p (236)	α-tubulin (401)	0.001	8
VVNGPVTKNR(8)	FDLMYAKR(7)	Dam1p (307)	α-tubulin (401)	0.006	11
DAAPAKPPNQGLNPR(6)	FDLMYAKR(7)	Duo1p (183)	α-tubulin (401)	0.008	15
ASQNKQFNQPLFPSSQVR(5)	FDLMYAKR(7)	Dam1p (171)	α-tubulin (401)	0.005	6
VVNGPVTKNR(8)	GDVVPKDVNAAIATIK(6)	Dam1p (307)	α-tubulin (326)	0.005	4
KPTESR(1)	FDLMYAKR(7)	Dam1p (320)	α-tubulin (401)	0.006	2
WTKPTASSSR(3)	ALTVPELTQQMFDAKNMMAACDPR(15)	Duo1p (236)	β-tubulin† (297)	0	3
DAAPAKPPNQGLNPR(6)	GHYTIGKELIDLVLDR(7)	Duo1p (183)	α-tubulin (112)	0	7
KPTESR(1)	GHYTIGKEIIDLVLDR(7)	Dam1p (320)	α-tubulin (112)	0	4
WTKPTASSSR(3)	GHYTIGKEIIDLVLDR(7)	Duo1p (236)	α-tubulin (112)	0.005	1

KSILHTIR(1)	VGINYQPPTVVPGGDLAKVQR (18)	Dam1p (256)	α-tubulin (370)	0.005	1
KINTRPPFR(1)	ALTVPELTQQMFDSKNMMA ACDPR(15)	Dam1p (335)	β-tubulin† (297)	0.001	1
KTHVPASK(1)	GHYTIGKEIIDLVLDR(7)	Duo1p (215)	α-tubulin (112)	0.004	5

Tubulin accession numbers: α-tubulin = gi|87578350; β-tubulin* = gi|75773583; β-tubulin† = gi|116004471.

Supplementary Table 10: Tubulin to tubulin cross-links found at 1% FDR.

Peptide 1 (position)	Peptide 2 (position)	Protein 1 (position)	Protein 2 (position)	Peptide Q-Value	Num PSMs	Cα-Cα distance
GDVVPKDVNAAIATIK(6)	TLKLTTPTYGDLNHLVSATMSGVTTCLR(3)	α-tubulin(326)	β-tubulin*(216)	0.001	12	13.3
QLFHPEQLITGKEDAANNYAR(12)	MSMKEVDEQMLNVQNK(4)	α-tubulin(96)	β-tubulin*(324)	0	13	20.4
AYHEQLSVAEITNACFEPANQMVKCDPR(24)	MSMKEVDEQMLNVQNK(4)	α-tubulin(304)	β-tubulin†(324)	0	9	15.1
QLFHPEQLITGKEDAANNYAR(12)	GDVVPKDVNAAIATIK(6)	α-tubulin(96)	α-tubulin(326)	0	10	19.5
QLFHPEQLITGKEDAANNYAR(12)	EVDEQMLNVQNKNSSYFVEWIPNNVK(12)	α-tubulin(96)	β-tubulin*(336)	0	3	21.8
GLKMSATFIGNSTAIQELFK(3)	INVYYNEAAGNKYVPR(12)	β-tubulin†(362)	β-tubulin†(58)	0.001	3	15.0
QLFHPEQLITGKEDAANNYAR(12)	TLKLATPTYGDLNHLVSATMSGVTTSLR(3)	α-tubulin(96)	β-tubulin†(216)	0.001	1	14.9

Tubulin accession numbers: α-tubulin = gi|87578350; β-tubulin* = gi|75773583; β-tubulin† = gi|116004471.

Supplementary Table 11: Fit of Dam1 complex to microtubule cross-links on the Dam1 complex models made from cross-linking data gathered in the presence or absence of microtubules.

Protein 1 (position)	Protein 2 (position)	distance between beads (Å) minus MT model	distance between beads (Å) plus MT model
Dam1p (171)	α-tubulin (401)	17.7	8.4
Duo1p (183)	α-tubulin (112)	10.4	4.0
Duo1p (183)	α-tubulin (401)	23.1	-4.0
Duo1p (215)	α-tubulin (112)	3.3	-3.8
Duo1p (215)	α-tubulin (370)	20.1	9.3
Duo1p (215)	α-tubulin (401)	8.9	5.4
Duo1p (236)	α-tubulin (112)	9.7	11.6
Duo1p (236)	β-tubulin (216)	27.5	17.1
Duo1p (236)	β-tubulin (297)	20.0	6.2
Duo1p (236)	β-tubulin (324)	12.0	6.8
Duo1p (236)	α-tubulin (326)	16.6	24.7
Duo1p (236)	α-tubulin (401)	2.96	3.0
Dam1p (252)	α-tubulin (401)	12.3	0.9
Dam1p (256)	α-tubulin (370)	27.2	20.1
Dam1p (256)	α-tubulin (401)	6.6	6.2
Dam1p (307)	α-tubulin (326)	19.4	12.1
Dam1p (307)	α-tubulin (401)	8.5	0.6
Dam1p (320)	α-tubulin (112)	27.3	7.5
Dam1p (320)	α-tubulin (401)	25.2	6.4
Dam1p (330)	α-tubulin (401)	22.2	4.7
Dam1p (335)	β-tubulin (297)	26.7	0.8
**Overall fit to data (%)**		**28.6**	**76.2**

Minus values indicate a slight overlap in bead positions. Values 10 Å and under are regarded as a fit.

Supplementary Table 12: Macro-regions of the Dam1 complex.

Protein name	Macro-region ID	Starting Residue	Ending Residue
Ask1p	1 Ask1N	1	97
Ask1p	2 Ask1M	98	194
Ask1p	3 Ask1C	195	292
Dad1p	1 Dad1	1	94
Dad2p	1 Dad2	1	133
Dad3p	1 Dad3	1	94
Dad4p	1 Dad4	1	72
Dam1p	1 Dam1N	1	114
Dam1p	2 Dam1M	115	228
Dam1p	3 Dam1C	229	343
Duo1p	1 Duo1N	1	123
Duo1p	2 Duo1C	124	247
Hsk3p	1 Hsk3	1	69
Spc19p	1 Spc19N	1	82
Spc19p	2 Spc19C	83	165
Spc34p	1 Spc34N	1	98
Spc34p	2 Spc34M	99	196
Spc34p	3 Spc34C	197	295

Supplemental Table 13: A comparison of Dam1 complex interactions in the presence and absence of microtubules. Data are presented as macro-regions as defined in Supplementary Table 11. Regions referred to in main text are highlighted in blue, lilac, green and grey. Each pair of beads, in a pair of macro-regions, with modeled bead surfaces within 8 Å counts as an interaction.

macro-region A	macro-region B	Dam1 modeled minus MTs (v19)				Dam1 modeled plus MTs (v21)		
		# cross-links	# mono inter-actions	# dimer inter-actions		# cross-links	# mono inter-actions	# dimer inter-actions
Ask1N	Ask1N	4	16	0		2	8	0
Ask1N	Ask1M	0	26	0		0	14	0
Ask1N	Ask1C	1	7	1		0	6	0
Ask1N	Dad1	0	3	0		0	0	0
Ask1N	Dad2	2	4	0		0	1	6
Ask1N	Dad3	4	5	0		1	0	1
Ask1N	Dad4	1	7	0		0	0	0
Ask1N	Dam1N	1	0	0		0	1	4
Ask1N	Dam1M	3	0	20		2	0	4
Ask1N	Dam1C	3	3	4		1	0	0
Ask1N	Duo1N	2	0	0		1	0	7
Ask1N	Duo1C	1	4	4		0	0	5
Ask1N	Hsk3	1	10	0		0	0	0
Ask1N	Spc19N	0	16	0		0	1	0
Ask1N	Spc19C	0	13	0		1	16	0
Ask1N	Spc34N	0	0	6		0	0	7
Ask1N	Spc34M	0	0	1		0	0	0
Ask1N	Spc34C	0	0	4		1	0	7
Ask1M	Ask1M	0	20	0		0	22	0
Ask1M	Ask1C	3	14	0		2	13	7
Ask1M	Dad1	0	0	0		1	0	9
Ask1M	Dad2	0	4	0		1	1	15
Ask1M	Dad3	1	1	0		2	0	2
Ask1M	Dad4	1	5	0		1	0	0
Ask1M	Dam1N	0	0	0		1	0	8
Ask1M	Dam1M	1	0	3		0	0	1

Ask1M	Dam1C	0	0	1		0	0	3
Ask1M	Duo1N	0	0	0		1	0	2
Ask1M	Duo1C	0	8	0		0	0	8
Ask1M	Hsk3	0	4	0		0	0	0
Ask1M	Spc19N	0	17	0		0	5	0
Ask1M	Spc19C	0	2	0		1	5	0
Ask1M	Spc34N	0	0	1		1	0	3
Ask1M	Spc34M	0	0	0		0	0	0
Ask1M	Spc34C	0	0	0		1	0	8
Ask1C	Ask1C	16	26	0		14	25	0
Ask1C	Dad1	3	0	0		8	12	2
Ask1C	Dad2	0	23	0		0	12	1
Ask1C	Dad3	3	4	0		4	2	0
Ask1C	Dad4	1	2	0		1	2	0
Ask1C	Dam1N	0	0	0		2	10	1
Ask1C	Dam1M	0	1	0		1	0	0
Ask1C	Dam1C	1	0	0		1	1	3
Ask1C	Duo1N	0	10	0		0	0	1
Ask1C	Duo1C	2	33	0		1	0	11
Ask1C	Hsk3	0	0	0		0	0	0
Ask1C	Spc19N	0	14	0		1	22	0
Ask1C	Spc19C	0	0	0		2	10	0
Ask1C	Spc34N	0	0	5		1	0	7
Ask1C	Spc34M	0	0	10		0	0	0
Ask1C	Spc34C	0	0	0		2	0	10
Dad1	Dad1	12	17	0		8	14	0
Dad1	Dad2	6	2	0		3	7	0
Dad1	Dad3	29	28	0		11	9	0
Dad1	Dad4	7	5	0		6	6	0
Dad1	Dam1N	6	3	0		6	6	0
Dad1	Dam1M	0	0	0		0	3	0
Dad1	Dam1C	2	12	0		1	15	0
Dad1	Duo1N	12	12	0		9	5	0
Dad1	Duo1C	9	7	0		3	6	0
Dad1	Hsk3	3	8	0		0	2	0
Dad1	Spc19N	5	3	0		1	11	0

Dad1	Spc19C	2	20	0		2	0	0
Dad1	Spc34N	6	0	17		4	1	3
Dad1	Spc34M	5	0	6		6	0	1
Dad1	Spc34C	4	0	13		6	2	0
Dad2	Dad2	6	25	0		3	17	0
Dad2	Dad3	11	11	0		2	11	0
Dad2	Dad4	5	4	0		2	2	0
Dad2	Dam1N	0	0	0		0	23	0
Dad2	Dam1M	1	2	0		2	0	0
Dad2	Dam1C	2	8	0		0	0	0
Dad2	Duo1N	3	13	0		0	5	0
Dad2	Duo1C	6	18	0		0	1	0
Dad2	Hsk3	2	2	0		0	0	0
Dad2	Spc19N	5	15	0		3	3	0
Dad2	Spc19C	0	0	0		2	4	3
Dad2	Spc34N	3	0	8		0	2	9
Dad2	Spc34M	0	0	30		0	0	5
Dad2	Spc34C	0	0	0		1	1	1
Dad3	Dad3	23	21	0		10	13	0
Dad3	Dad4	12	8	0		8	8	0
Dad3	Dam1N	5	8	0		3	9	0
Dad3	Dam1M	2	2	0		0	14	0
Dad3	Dam1C	4	31	0		0	7	0
Dad3	Duo1N	15	24	0		6	22	0
Dad3	Duo1C	23	21	0		0	6	0
Dad3	Hsk3	7	8	0		2	2	0
Dad3	Spc19N	9	11	0		1	3	0
Dad3	Spc19C	2	12	0		3	0	0
Dad3	Spc34N	10	0	33		11	1	17
Dad3	Spc34M	8	0	14		6	0	16
Dad3	Spc34C	2	0	4		2	6	0
Dad4	Dad4	3	9	0		3	8	0
Dad4	Dam1N	4	2	0		4	1	0
Dad4	Dam1M	0	5	0		0	11	0
Dad4	Dam1C	4	11	0		2	5	0
Dad4	Duo1N	2	6	0		2	1	0

Dad4	Duo1C	5	11	0		0	0	0
Dad4	Hsk3	4	24	0		2	7	0
Dad4	Spc19N	3	15	0		0	6	0
Dad4	Spc19C	0	2	0		2	1	0
Dad4	Spc34N	2	0	3		0	0	8
Dad4	Spc34M	0	0	1		0	0	11
Dad4	Spc34C	2	0	2		0	1	0
Dam1N	Dam1N	4	17	0		4	21	0
Dam1N	Dam1M	0	3	0		0	1	0
Dam1N	Dam1C	0	32	0		0	0	0
Dam1N	Duo1N	7	33	0		4	31	0
Dam1N	Duo1C	0	12	0		0	2	0
Dam1N	Hsk3	0	3	0		0	0	0
Dam1N	Spc19N	2	1	0		4	1	0
Dam1N	Spc19C	0	0	7		1	0	1
Dam1N	Spc34N	3	5	2		5	2	0
Dam1N	Spc34M	0	0	1		0	0	3
Dam1N	Spc34C	0	13	0		0	1	0
Dam1M	Dam1M	14	24	0		11	27	0
Dam1M	Dam1C	17	24	0		13	27	0
Dam1M	Duo1N	1	4	0		0	13	0
Dam1M	Duo1C	33	40	0		26	21	0
Dam1M	Hsk3	1	11	0		0	7	0
Dam1M	Spc19N	1	8	1		0	0	1
Dam1M	Spc19C	0	0	3		1	0	8
Dam1M	Spc34N	0	5	0		0	1	2
Dam1M	Spc34M	0	0	0		0	4	0
Dam1M	Spc34C	0	1	0		2	25	0
Dam1C	Dam1C	18	25	0		16	26	0
Dam1C	Duo1N	0	36	0		0	2	0
Dam1C	Duo1C	18	44	0		19	55	0
Dam1C	Hsk3	1	14	1		1	16	0
Dam1C	Spc19N	7	6	0		4	1	12
Dam1C	Spc19C	2	1	5		1	0	5
Dam1C	Spc34N	8	4	12		3	0	0
Dam1C	Spc34M	3	0	8		2	3	0

Dam1C	Spc34C	9	1	0		5	10	0
Duo1N	Duo1N	10	29	0		4	28	0
Duo1N	Duo1C	3	31	0		0	11	0
Duo1N	Hsk3	1	6	0		0	0	0
Duo1N	Spc19N	0	1	0		0	0	0
Duo1N	Spc19C	0	1	1		3	0	0
Duo1N	Spc34N	8	1	6		6	6	2
Duo1N	Spc34M	1	0	5		0	1	3
Duo1N	Spc34C	0	0	0		3	6	0
Duo1C	Duo1C	29	54	0		24	30	0
Duo1C	Hsk3	3	9	0		0	4	0
Duo1C	Spc19N	6	18	0		3	0	19
Duo1C	Spc19C	0	0	0		5	0	19
Duo1C	Spc34N	4	6	4		1	10	0
Duo1C	Spc34M	1	0	6		0	3	0
Duo1C	Spc34C	2	0	0		8	25	0
Hsk3	Hsk3	3	12	0		2	7	0
Hsk3	Spc19N	4	11	0		2	4	0
Hsk3	Spc19C	0	9	0		0	0	0
Hsk3	Spc34N	1	0	0		0	0	0
Hsk3	Spc34M	0	0	0		0	0	0
Hsk3	Spc34C	0	0	1		0	0	0
Spc19N	Spc19N	3	11	0		2	19	0
Spc19N	Spc19C	2	11	0		1	26	0
Spc19N	Spc34N	5	0	20		2	0	6
Spc19N	Spc34M	5	0	17		2	0	7
Spc19N	Spc34C	2	0	9		1	0	11
Spc19C	Spc19C	18	22	0		15	22	0
Spc19C	Spc34N	1	0	29		3	0	29
Spc19C	Spc34M	0	0	8		1	0	8
Spc19C	Spc34C	11	0	34		10	0	36
Spc34N	Spc34N	15	18	0		11	16	0
Spc34N	Spc34M	12	31	0		5	29	0
Spc34N	Spc34C	0	23	0		2	19	0
Spc34M	Spc34M	7	28	0		6	23	0
Spc34M	Spc34C	3	9	0		0	8	0

Spc34C	Spc34C	11	19	0		7	12	0
**totals**		**678**	**1475**	**371**		**458**	**1083**	**389**
**% of total**			**79.9**	**20.1**			**73.6**	**26.4**

Supplementary Table 14: Amino acid sequences of the proteins used in this paper. All distance constraint location numbering is based on these sequences.

**BRCA1** (132 amino acids)<br>MGSSHHHLHHSSGXVPRGSHMDLSALRVEEVQNVINAMQKILECPICLELIKEPVSTKCDHIFCKFCMLKLLNQKKGPS<br>QCPLCKNDITKRSLQESTRFSQLVEELLKIICAFQLDTGLEYANSYNFAKKGK
**BARD1** (115 amino acids)<br>MEPDGRGAWAHSRAALDRLEKLLRCSRCTNILREPVCLGGCEHIFCSNCVSDCIGTGCPVCYTPAWIQDLKINRQLDSM<br>IQLCSKLRNLLHDNELSDLKEDKPRKSLFNDAGNKK
**Fbxl3** (430 amino acids)<br>GSMKRGGRDSDRNSSEEGTAEKSKKLRTTNEHSQTCDWGNLLQDIILQVFKYLPLLDRAHASQVCRNWNQVFHMPD<br>LWRCFEFELNQPATSYLKATHPELIKQIIKRHSNHLQYVSFKVDSSKESAEAACDILSQLVNCSLKTLGLISTARPSFMDLPK<br>SHFISALTVVFVNSKSLSSLKIDDTPVDDPSLKVLVANNSDTLKLLKMSSCPHVSPAGILCVADQCHGLRELALNYHLLSDE<br>LLLALSSEKHVRLEHLRIDVVSENPGQTHFHTIQKSSWDAFIRHSPKVNLVMYFFLYEEEFDPFFRYEIPATHLYFGRSVSK<br>DVLGRVGMTCPRLVELVVCANGLRPLDEELIRIAERCKNLSAIGLGECEVSCSAFVEFVKMCGGRLSQLSIMEEVLIPDQK<br>YSLEQIHWEVSKHLGRVWFPDMMPTW
**mCRY2 1-544** (544 amino acids)<br>MAAAAVVAATVPAQSMGADGASSVHWFRKGLRLHDNPALLAAVRGARCVRCVYILDPWFAASSSVGINRWRFLLQS<br>LEDLDTSLRKLNSRLFVVRGQPADVFPRLFKEWGVTRLTFEYDSEPFGKERDAAIMKMAKEAGVEVVTENSHTLYDLDR<br>IIELNGQKPPLTYKRFQALISRMELPKKPAVAVSSQQMESCRAEIQENHDDTYGVPSLEELGFPTEGLGPAVWQGGETE<br>ALARLDKHLERKAWVANYERPRMNANSLLASPTGLSPYLRFGCLSCRLFYYRLWDLYKKVKRNSTPPLSLFGQLLWREFF<br>YTAATNNPRFDRMEGNPICIQIPWDRNPEALAKWAEGKTGFPWIDAIMTQLRQEGWIHHLARHAVACFLTRGDLWV<br>SWESGVRVFDELLLDADFSVNAGSWMWLSCSAFFQQFFHCYCPVGFGRRTDPSGDYIRRYLPKLKGFPSRYIYEPWNA<br>PESVQKAAKCIIGVDYPRPIVNHAETSRLNIERMKQIYQQLSRYRGLCLLASVPSCVEDLSHPVAEPGSSQAGSIS
**Skp1d** (149 amino acids)<br>MPSIKLQSSDGEIFEVDVEIAKQSVTIKTMLEDLGMDPVPLPNVNAAILKKVIQWCTHHKDDPGGSGTDDIPVWDQEF<br>LKVDQGTLFELILAANYLDIKGLLDVTCKTVANMIKGKTPEEIRKTFNIKNDFTEEEEAQVRKENQWCEEK
**Spc97p** (823 amino acids)<br>MEIKEVDDRAELLRYTNNIPLLGKLVNHQPLWSTNPKLKSFSLEKISAPDQRRVQEALVVKDLLNVLIGLEGTYIRYFNDY<br>EPSDPETPIEFKIAKKMDPSFKTFSRRIVRYGKQYMILTRAYEKWSDTSFGMVLQRFAYEIRRFLEDVYLKTLVERLERDFN<br>KVPNFSIRELEQIINETEVNKQMELLYNIYEEIFREIEERRTNQSSQEDFNNFMDSMKNESSLHLRLMVAFDTTVYPVPKG<br>GAILKIFQQKILENLGDRSSVMFLKKLLNNISQDYCTMLYEWLTQGILNDPYQEFMTYDDLEGKTDNIFDTRDRAWDTQ<br>YFIRKDVLLRDCDSEEDKNLLFKMLRTGILLKVVRASLQIPTIPSNSSDITIQEINDFADLMEGSNLELYVDKCYSRANEIFLK<br>LFFQGYDLINVLKHLQQIFLGYQSGHNVLKFLTKNMGELTKHYRNDNNANYDKLLQNFELERQSENPNNLMRQLLMIQ<br>FDTETLPQVLSHYLQIYPEVPENNSANDDSDPLMHANNFKNMNAILFDELSKERTGAYHGSNLELYTPKSAIYHLKFDINI<br>PYPLNIIISRTCMIKYQIILRYQLVLQYHSRLLDETWMDLNKTPSWKYRGYSHTVKRRIVRATRVLHAKMNHFIKTIMEYF<br>NQNVIDKEVYSLEKCYRNPTLAVAIQNELEGGLTNIMTNRCLSDLIPLQLQIFDIVYKFCKFIKSMRAKLCQLDPVLYEKHK<br>SGMMKTLNEGYRTNNGGQEDVGYQEDAALELIQKLIEYISNASSIFRKCLINFTQELSTEKFDFYDSSSVDAAGIERVLYSI<br>VPPRSASASSQR
**Spc98p** (846 amino acids)<br>MELEPTLFGIIEALAPQLLSQSHLQTFVSDVVNLLRSSTKSATQLGPLIDFYKLQSLDSPETTIMWHKIEKFLDALFGIQNT<br>DDMVKYLSVFQSLLPSNYRAKIVQKSSGLNMENLANHEHLLSPVRAPSIYTEASFENMDRFSERRSMVSSPNRYVPSST<br>YSSVTLRQLSNPYYVNTIPEEDILKYVSYTLLATTSALFPFDHEQIQIPSKIPNFESGLLHLIFEAGLLYQSLGYKVEKFRMLNI<br>SPMKKALIIEISEELQNYTAFVNNLVSSGTVVSLKSLYREIYENIIRLRIYCRFTEHLEELSGDTFLIELNIFKSHGDLTIRKIATN

LFNSMISLYYEYLMNWLTKGLLRATYGEFFIAENTDTNGTDDDFIYHIPIEFNQERVPAFIPKELAYKIFMIGKSYIFLEKYCKEVQWTNEFSKKYHVLYQSNSYRGISTNFFEIINDQYSEIVNHTNQILNQKFHYRDVVFALKNILLMGKSDFMDALIEKANDILATPSDSLPNYKLTRVLQEAVQLSSLRHLMNSPRNSSVINGLDARVLDLGHGSVGWDVFTLDYILYPPLSLVLNVNRPFGRKEYLRIFNFLWRFKKNNYFYQKEMLKSNDIIRSFKKIRGYNPLIRDIINKLSRISILRTQFQQFNSKMESYYLNCIIEENFKEMTRKLQRTENKSQNQFDLIRLNNGTIELNGILTPKAEVLTKSSSSKPQKHAIEKTLNIDELESVHNTFLTNILSHKLFATNTSEISVGDYSGQPYPTSLVLLLNSVYEFVKVYCNLNDIGYEIFIKMNLNDHEASNGLLGKFNTNLKEIVSQYKNFKDRLYIFRADLKNDGDEELFLLSKSLR
**His-TEV-Tub4** (506 amino acids)<br>MHHHHHHGKPIPNPLLGLDSTENLYFQGIDPFTMGGEIITLQAGQCGNHVGKFLWSQLAKEHAIGTDGLSQLPDSSTERDDDTKPFFRENSRNKFTPRAIMMDSEPSVIADVENTFRGFFDPRNTWVASDGASAGNSWANGYDIGTRNQDDILNKIDKEIDSTDNFEGFQLLHSVAGGTGSGLGSNLLEALCDRYPKKILTTYSVFPARSSEVVVQSYNTILALRRLIEDSDATVVFDNASLLNISGKVFRNPNIDLQHTNQLISTIISSVTNSIRFPSYMYSSMSSIYSTLIPSPELHFLSPSFTPFTSDYIHDDIAHKGHSSYDVMLDLLDPSNSLVSTAMNNPTYFNVYNTIIGNVEPRQISRAMTKLQQRIKFPSWSSSAMHVNIGRRSPYLPLQPNENEVSGMMLSNMSTVVNVFENACNTFDKVFAKGAFLNNYNVGDLFQSMQNVQDEFAESREVVQSLMEDYVAAEQDSYLDDVLVDDENMVGELEEDLDADGDHKLV
**Ask1p** (292 amino acids)<br>MDSASKEETLEKLDQEITVNLQKIDSNLSFCFHKITQDIIPHVATYSEICERIMDSTEWLGTMFQETGLVNLQANAAAPVGNAPVKSLVSNNVGIFPTSAEEASRQSQTDNGPNEADSAVHVNRDVHSMFNNDSIDDFHTANITSTGQILKLPDSSDEDTGSEAVPSREQTDLTGEGHGGADDEQDESTIQRQSRKRKISLLLQQQYGSSSSMVPSPIVPNKMRKQLAHEEHINNDGDNDDENSNNIESSPLKQGHHHPKGQADDNNEGPDEEESTKEVPKPGTIIHFSTNR
**Dad1p** (94 amino acids)<br>MMASTSNDEEKLISTTDKYFIEQRNIVLQEINETMNSILNGLNGLNISLESSIAVGREFQSVSDLWKTLYDGLESLSDEAPIDEQPTLSQSKTK
**Dad2p** (133 amino acids)<br>MDSIDEQIAIKRKELQSLQKITSLTDGLKIQLIELNEQIKEMGMNADSVAQLMNNWDSIINNISQASLGLLQYAEGDYEIGPWKDSKKNESEQSNETGLEAQENDKNDEDNDEDEDLVPLPETMVRIRVDGNE
**Dad3p** (94 amino acids)<br>MEHNLSPLQQEVLDKYKQLSLDLKALDETIKELNYSQHRQQHSQQETVSPDEILQEMRDIEVKIGLVGTLLKGSVYSLILQRKQEQESLGSNSK
**Dad4p** (72 amino acids)<br>MENPHEQVQANILSRIIGNVKRLNESVAILNQELVTINNRNKNLEIMGAICDNYHSSVQFNLEATNNKKPPL
**Dam1p** (343 amino acids)<br>MSEDKAKLGTTRSATEYRLSIGSAPTSRRSSMGESSSLMKFADQEGLTSSVGEYNENTIQQLLLPKIRELSDSIITLDSNFTRLNFIHESLADLNESLGSLLYGIMSNSWCVEFSQAPHDIQDDLIAIKQLKSLEDEKNNLVMELSNMERGIKRKKDEQGENDLAKASQNKQFNQPLFPSSQVRKYRSYDNRDKRKPSKIGNNLQVENEEDYEDDTSSEASFVLNPTNIGMSKSSQGHVTKTTRLNNNTNSKLRRKSILHTIRNSIASGADLPIENDNVVNLGDLHPNNRISLGSGAARVVNGPVTKNRNSMFSGRAERKPTESRHSVAKKTEKKINTRPPFR
**Duo1p** (247 amino acids)<br>MSEQSQLDDSTIDKLIPQIFNEMRSNLNNTTNKFPKSTGGGASDNISANSNSIRSFNSITTQSLLKESESLDKITAMIKNVTAALKNNLPVYVNQVHEVCKSTNSILDSWINIHSQAGYIHKLMSDQTYLKLINDRLHNENVNTNDEDGSTLHNVIALKKKEILDLRQKLENRKGEKDAAPAKPPNQGLNPRYGVQSGRRPVPSAGISNNGRVRKTHVPASKRPSGIPRVTNRWTKPTASSSRKMFR

**Hsk3p** (69 amino acids)<br>MNANKQRQYNQLAHELRELQTNLQETTKQLDIMSKQCNENLVGQLGKVHGSWLIGSYIYYMEQMLGKTQ
**Spc19p** (165 amino acids)<br>MTDALEQSVLALEGTVSVLKDSVESLKCANEPSTNLASTMLQTKRVFRLVPEYDVERSKLDLIEEVEPLVRTLGDKLRKSM<br>GRMQRELDTLQQTYELNDLRLKKNISMDDDDALNSPDMGQEYEGRDADDVVMMASSTNEELEELKKLKEKKKQLEN<br>KLEILKQK
**Spc34(-His)** (301 amino acids)<br>MGESLDRCIDDINRAVDSMSTLYFKPPGIFHNAILQGASNKASIRKDITRLIKDCNHDEAYLLFKVNPEKQSVSRRDGKEG<br>VFDYVIKRDTDMKRNRRLGRPGEKPIIHVPKEVYLNKDRLDLNNKRRRTATTSGGGLNGFIFDTDLIGSSVISNSSSGTFK<br>ALSAVFKDDPQIQRLLYALENGSVLMEEESNNQRRKTIFVEDFPTDLILKVMAEVTDLWPLTEFKQDYDQLYHNYEQLSS<br>KLRFIKKEVLLQDDRLKTMSQYHPSSSHDVAKIIRKEKDEIRRLEMEIANLQEHHHHHH
**gi|87578350 (Tubulin, alpha 3d [Bos taurus])** (452 amino acids)<br>MRECISVHVGQAGVQIGNACWELYCLEHGIQPDGQMPSDKTIGGGDDSFNTFFSETGAGKHVPRAVFVDLEPTVIDE<br>VRTGTYRQLFHPEQLITGKEDAANNYARGHYTIGKELIDLVLDRIRKLADQCTGLQGFLIFHSFGGGTGSGFTSLLMERLS<br>VDYGKKSKLEFSIYPAPQVSTAVVEPYNSILTTHTTLEHSDCAFMVDNEAIYDICRRNLDIERPTYTNLNRLIGQIVSSITASL<br>RFDGALNVDLTEFQTNLVPYPRIHFPLATYAPVISAEKAYHEQLSVAEITNACFEPANQMVKCDPRHGKYMACCLLYRG<br>DVVPKDVNAAIATIKTKRTIQFVDWCPTGFKVGINYQPPTVVPGGDLAKVQRAVCMLSNTTAIAEAWARLDHKFDLM<br>YAKRAFVHWYVGEGMEEGEFSEAREDMAALEKDYEEVGMDSVEGEGEEEEGDEY
**gi|312597141 (tubulin beta-chain [Bos taurus])** (427 amino acids)<br>MREIVHIQAGQCGNQIGAKFWEVISDEHGIDPTGSYHGDSDLQLERINVYYNEAAGNKYVPRAILVDLEPGTMDSVRS<br>GPFGQIFRPDNFVFGQSGAGNNWAKGHYTEGAELVDSVLDVVRKESESCDCLQGFQLTHSLGGGTGSGMGTLLISKIR<br>EEYPDRIMNTFSVVPSPKVSDTVVEPYNATLSVHQLVENTDETYCIDNEALYDICFRTLKLTTPTYGDLNHLVSATMSGVT<br>TCLRFPGQLNADLRKLAVNMVPFPRLHFFMPGFAPLTSRGSQQYRALTVPELTQQMFDAKNMMAACDPRHGRYLTV<br>AAVFRGRMSMKEVDEQMLNVQNKNSSYFVEWIPNNVKTAVCDIPPRGLKMSATFIGNSTAIQELFKRISEQFTAMFR<br>RKAFLHWYTGEGMDEMEFTEAESNMNDLVSEYQQYQD
**gi|116004471 (tubulin beta-3 chain [Bos taurus])** (450 amino acids)<br>MREIVHIQAGQCGNQIGAKFWEVISDEHGIDPSGNYVGDSDLQLERISVYYNEASSHKYVPRAILVDLEP<br>GTMDSVRSGAFGHLFRPDNFIFGQSGAGNNWAKGHYTEGAELVDSVLDVVRKECENCDCLQGFQLTHSLG<br>GGTGSGMGTLLISKVREEYPDRIMNTFSVVPSPKVSDTVVEPYNATLSIHQLVENTDETYCIDNEALYDI<br>CFRTLKLATPTYGDLNHLVSATMSGVTTSLRFPGQLNADLRKLAVNMVPFPRLHFFMPGFAPLTARGSQQ<br>YRALTVPELTQQMFDAKNMMAACDPRHGRYLTVATVFRGRMSMKEVDEQMLAIQSKNSSYFVEWIPNNVKVAVCD<br>IPPRGLKMSSTFIGNSTAIQELFKRISEQFTAMFRRKAFLHWYTGEGMDEMEFTEAESNMNDLVS<br>EYQQYQDATAEEEGEMYEDDEEESEAQGPK
**gi|75773583 (tubulin, beta 2C [Bos taurus])** (450 amino acids)<br>MREIVHIQAGQCGNQIGAKFWEVISDEHGIDPSGNYVGDSDLQLERISVYYNEASSHKYVPRAILVDLEP<br>GTMDSVRSGAFGHLFRPDNFIFGQSGAGNNWAKGHYTEGAELVDSVLDVVRKECENCDCLQGFQLTHSLG<br>GGTGSGMGTLLISKVREEYPDRIMNTFSVVPSPKVSDTVVEPYNATLSIHQLVENTDETYCIDNEALYDI<br>CFRTLKLATPTYGDLNHLVSATMSGVTTSLRFPGQLNADLRKLAVNMVPFPRLHFFMPGFAPLTARGSQQ<br>YRALTVPELTQQMFDAKNMMAACDPRHGRYLTVATVFRGRMSMKEVDEQMLAIQSKNSSYFVEWIPNNVKVAVCD<br>IPPRGLKMSSTFIGNSTAIQELFKRISEQFTAMFRRKAFLHWYTGEGMDEMEFTEAESNMNDLVS

EYQQYQDATAEEEGEMYEDDEEESEAQGPK

Supplementary Table 15: The percentage of SCF^{FBXL3} cross-linking data that fits equivalent distance cutoffs at course-grained and atomic resolutions. Course-grained cutoffs represent the distance between the surfaces of the two beads containing the cross-linked residues. Atomic cutoffs refer to the Cα-Cα distance of the cross-linked lysines.

Distance restraints within cutoff	86%		91%		95%	
Structural representation	atomic	course-grained	atomic	course-grained	atomic	course-grained
Cutoff (Å)	21	5	30	8	31	10

Supplementary Notes:

Supplementary Note 1: Cooperative binding models by Hill, McGhee and von Hippel are unable to distinguish changes in cooperativity from direct binding of the Dam1 complex to microtubules in bulk binding assays.

Previous work from our lab used bulk binding assays[8, 9] to define how Aurora B phosphorylation of the Dam1 complex inhibits its ability to bind microtubules. The Dam1 complex binds microtubules in a highly cooperative manner and thus the apparent affinity represents a combination of two factors: direct interaction between an individual Dam1 complex and the microtubule, and the interaction between two or more adjacent Dam1 complexes (oligomerization). In an attempt to separate the contributions of these two factors to the apparent affinity, we fit our data to cooperative binding models[10, 11]. However, our new data presented here indicate that these models were unable to distinguish changes in cooperativity from direct binding in this case. We attribute this to the fact that neither model accurately describes the microtubule as a substrate, in which the ligand binding sites are present in linear repeating units (protofilaments) that form a closed tube. This arrangement introduces an extra dimension that is not accounted for in the closest approximation, as described in the McGhee & von Hippel model, for protein binding to a linear DNA substrate.

Supplementary Methods:

Electron Microscopy. Dam1 complex on microtubules was produced and cross-linked exactly as described above in the cross-linking section except that after quenching, the reaction was diluted 21-fold with warm BRB80 containing 10 µM taxol. Carbon-coated copper grids were positively charged in a glow discharge device (Electron Microscopy Sciences) for 2 min. A 2 µl drop of sample was applied onto a freshly discharged grid and incubated for 20 s. Excess solution was blotted off, and the grid washed twice with BRB80 before staining with 2% uranyl acetate. Stain was blotted off, and the grid was air dried. Samples were viewed on a transmission electron microscope (Morgagni; FEI) operating at 100 kV. Images were recorded on a 1,000 x 1,000 bottom-mount slow-scan charge-coupled device camera (Gatan) at a nominal magnification 22,000x at the specimen level.

Kojak Search Parameters. Cross-linked peptides were identified using the Kojak version 1.0 cross-link identification software[6] following the author's instructions (http://www.kojak-ms.org) and using the search parameters listed below. Parameters marked [varies] were specific to each MS file searched. The exact Kojak search parameters used for each raw MS data file are available to download, along with the raw MS data at: http://proxl.yeastrc.org/dam1-zelter-2015.

```
# Kojak version 1.0 Search Parameters
# Data input files: include full path if not in current working directory
database                =       searchDatabase.fasta [varies]
MS_data_file            =       MS_data.mzML [varies]
output_file             =       KojakOutPutFileName.txt [varies]
percolator_file         =       KojakOutputExportedForPercolatorInput.txt [varies]
# Parameters used to described the data being input to Kojak
instrument              =       0       #0=Orbitrap, 1=FTICR
MS1_centroid            =       0       #0=no, 1=yes
MS2_centroid            =       0       #0=no, 1=yes [varies]
MS1_resolution          =       50000   #Resolution at 400 m/z [varies]
MS2_resolution          =       25000   #Resolution at 400 m/z [varies]
enrichment              =       0       #Values between 0 and 1 to describe 18O APE [varies]
# Cross-link and mono-link masses allowed. May have more than one of each parameter.
cross_link_mass =       138.0680742
mono_link_mass          =       155.0946
mono_link_mass          =       156.0786
# Fixed modifications
fixed_modification      =       C 57.02146
# Differential modifications. @ = N-terminus
max_mods_per_peptide    =       2
modification            =       K       14.015894
modification            =       K       28.031788
modification            =       K       42.047682
modification            =       M       15.9949
modification            =       @       42.01055
# Parameters used in Kojak analysis
```

```
prefer_precursor_pred   =       1       # 1=on, 0=off. [varies]
decoy_filter            =       random  #identifier for all decoys in the database
max_miscleavages        =       6       #number of missed trypsin cleavages allowed
max_peptide_mass        =       10000.0 #largest allowed peptide mass in Daltons
min_peptide_mass        =       600.0   #lowest allowed peptide mass in Daltons
max_spectrum_peaks      =       500     #top N peaks to use during analysis. 0 uses all peaks
ppm_tolerance_pre       =       25.0    #mass tolerance on precursor when searching
ppm_tolerance_frag      =       10.0    #mass tolerance when scoring fragment ions
search_dimers           =       1       #0=no, 1=yes
relaxed_analysis        =       1       #0=no, 1=yes
top_count               =       500     #number top scoring peptides combined in relaxed analysis
spectrum_processing     =       1       #0=no, 1=yes
use_comet_xcorr =       1       #0=no, 1=yes
```

Statistical validation of Kojak results. PSMs automatically exported from Kojak[6] in tab-delimited text format were input into Percolator[1] (version 2.08) for statistical validation. The filename of this automatically exported Percolator input file is defined by the Kojak parameter, percolator_file, listed in the Kojak search parameters above. Percolator is a semi-supervised algorithm that assigns a statistically meaningful *q* value to each PSM through analysis of the target and decoy PSM distributions. Decoy PSMs derive from peptide sequences known to be false. In the current work the target databases consisted of all proteins identified in the sample analyzed (see Materials and Methods), while the decoy databases consisted of the corresponding set of reversed protein sequences. Cross-link PSMs were considered to be false if at least one of the peptides was from a decoy protein sequence. The top scoring PSM for each spectrum was used as input to Percolator. The following scoring metrics were used as parameters for Percolator.

Score: PSM score reported by Kojak
dScore: difference in score between the PSM and the next best match for that spectrum
NormRank: summed rank of the two peptides in the top scoring list
Charge: charge state of the precursor ion
Mass: mass (in Daltons) of the precursor ion
PPM: mass difference between observed precursor ion mass and PSM, in parts per million
LenShort: length of the short peptide sequence
LenLong: length of the long peptide sequence
Dimer: Is the PSM a non-covalent dimer of two peptides? (1=yes, 0=no)
Mono: Is the PSM a mono-linked peptide? (1=yes, 0=no)
Loop: Is the PSM a loop-linked peptide? (1=yes, 0=no)
Cross: Is the PSM two cross-linked peptides? (1=yes, 0=no)

Percolator was run on the Kojak produced file for input into percolator using the command:

percolator -X output.xml -j KojakOutputExportedForPercolatorInput.txt >./percout.txt

The data presented in this paper was filtered to show only hits to the target proteins that had a Percolator assigned peptide level *q* value <= 0.01. The complete, unfiltered list of all PSMs and their Percolator assigned *q* values is available at: http://proxl.yeastrc.org/dam1-zelter-2015

Supplementary References:

1. Kall L, Canterbury JD, Weston J, Noble WS, MacCoss MJ. Semi-supervised learning for peptide identification from shotgun proteomics datasets. *Nat Methods* **4**, 923-925 (2007).

2. Leitner A*, et al.* The molecular architecture of the eukaryotic chaperonin TRiC/CCT. *Structure* **20**, 814-825 (2012).

3. Yang B*, et al.* Identification of cross-linked peptides from complex samples. *Nat Methods* **9**, 904-906 (2012).

4. Chen ZA*, et al.* Architecture of the RNA polymerase II-TFIIF complex revealed by cross-linking and mass spectrometry. *The EMBO journal* **29**, 717-726 (2010).

5. Jennebach S, Herzog F, Aebersold R, Cramer P. Crosslinking-MS analysis reveals RNA polymerase I domain architecture and basis of rRNA cleavage. *Nucleic acids research* **40**, 5591-5601 (2012).

6. Hoopmann MR*, et al.* Kojak: Efficient analysis of chemically cross-linked protein complexes. *J Proteome Res* **15**, 2190-2198 (2015).

7. Tien JF*, et al.* Kinetochore biorientation in Saccharomyces cerevisiae requires a tightly folded conformation of the Ndc80 complex. *Genetics* **198**, 1483-1493 (2014).

8. Gestaut DR*, et al.* Phosphoregulation and depolymerization-driven movement of the Dam1 complex do not require ring formation. *Nature cell biology* **10**, 407-414 (2008).

9. Graczyk B, Davis TN. An assay to measure the affinity of proteins for microtubules by quantitative fluorescent microscopy. *Analytical biochemistry* **410**, 313-315 (2011).

10. Hill AV. The possible effects of the aggregation of the molecules of haemoglobin on its dissociation curves. *J Physiol* **40**, iv–vii (1910).

11. McGhee JD, von Hippel PH. Theoretical aspects of DNA-protein interactions: co-operative and non-co-operative binding of large ligands to a one-dimensional homogeneous lattice. *Journal of molecular biology* **86**, 469-489 (1974).
